# Molecularly Built Ligands Degrade Membrane Receptors via Enhancing Their Accumulation in Lysosomes

**DOI:** 10.1021/acscentsci.5c01647

**Published:** 2025-11-28

**Authors:** Dongchen Zhang, Xinyi Zhou, Jiamin Cai, Weihong Tan, Yanlan Liu, Zilong Zhao

**Affiliations:** † Molecular Science and Biomedicine Laboratory (MBL), State Key Laboratory of Chemo and Biosensing, College of Chemistry and Chemical Engineering, College of Biology, Aptamer Engineering Center of Hunan Province, 214171 Hunan University, Changsha, Hunan 410082, People’s Republic of China; ‡ The Key Laboratory of Zhejiang Province for Aptamers and Theranostics, Zhejiang Cancer Hospital, Hangzhou Institute of Medicine (HIM), 12381 Chinese Academy of Sciences, Hangzhou, Zhejiang 310022, China

## Abstract

This study presents molecularly built ligand-based lysosome-targeting chimeras (MBL-LYTACs) as a versatile platform for membrane receptor degradation. MBL-LYTACs are engineered by conjugating targeting ligands (e.g., small molecules, oligopeptides, aptamers, nanobodies, and antibodies) with lysosome-localized molecules. Functional screening and mechanistic studies reveal that MBL-LYTACs significantly enhance internalization and lysosomal accumulation of membrane receptors, including folate receptor α, programmed death-ligand 1 (PD-L1), epidermal growth factor receptor (EGFR), and protein tyrosine kinase 7 (PTK7), by leveraging morpholine, dimethylethanamine, or low-polymerized mPEGs as lysosome-localized moieties, leading to receptor degradation. This approach eliminates reliance on cell-surface lysosome-shuttling receptors, broadening its applicability. The efficacy of MBL-LYTACs in cancer therapy has been validated in two tumor xenograft models, with PD-L1 and EGFR as targets. Overall, this study establishes a robust and adaptable framework for targeted receptor degradation, expanding therapeutic opportunities in cancer management.

## Graphical abstract

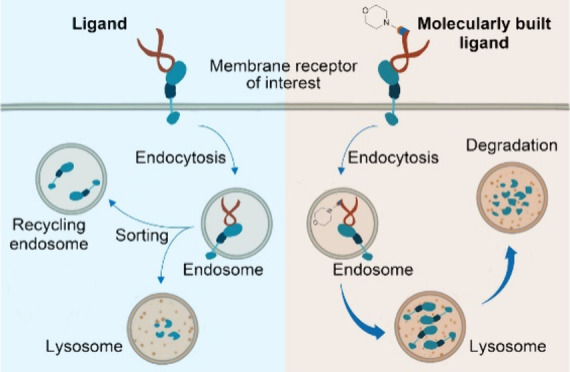

## Introduction

Plasma membrane receptors play critical roles in essential biological processes. Their dysfunction is closely linked to the initiation and progression of diseases such as cancer, cardiovascular disorders, and neurodegenerative conditions.^1–3^ As a result, membrane receptors have become major therapeutic targets, accounting for over 60% of clinical drug interventions.^4^ Conventional small-molecule and antibody-based inhibitors function via occupancy-driven pharmacology, competing with endogenous ligands for receptor binding.^5,6^ However, these inhibitors often fail against receptor variants that remain constitutively active due to gene mutation, amplification, or alternative splicing.^7,8^

Targeted protein degradation (TPD) has emerged as a promising therapeutic strategy, employing an event-driven mechanism of action to degrade proteins of interest (POIs) in proteasomes.^9,10^ In 2020, Bertozzi et al. introduced lysosome-targeting chimeras (LYTACs) to degrade extracellular proteins.^11^ In this pioneering work, LYTACs were engineered by conjugating small molecules or antibodies targeting pathogenic POIs with ligands recognizing cell-surface lysosome-shuttling receptors, e.g., cation-independent mannose-6-phosphate receptor (CI-M6PR). The approach successfully degraded membrane proteins like epidermal growth factor receptor (EGFR) and programmed death-ligand 1 (PD-L1). Since then, various LYTACs have been developed to tackle membrane receptors using different cell-surface lysosome-shuttling receptors, including CI-M6PR,^12^ asialoglycoprotein receptors,^13,14^ cytokine receptor,^15^ integrin receptor,^16,17^ and scavenger receptor.^18^ Compared to traditional inhibitors, TPD offers a broader target scope and higher therapeutic efficacy. However, its reliance on cell surface lysosome-shuttling receptors makes LYTACs cell-type-dependent, limiting their applications in cancers with high intertumor heterogeneity.^19,20^ Additionally, some lysosome-shuttling receptors, such as integrin αvβ3, are implicated in tumor progression.^21^ Their hyperactivation might mitigate the therapeutic efficacy and cause unintended side effects. Consequently, developing a lysosome-shuttling receptor-independent TPD strategy is an attractive avenue for further research.^22–24^

Membrane receptor internalization is a key process following ligand bindings. Once internalized, receptor–ligand complexes are directed through the endosomal-lysosomal pathway, where they are sorted for either recycling back to the plasma membrane or degradation in the lysosome.^25,26^ Lysosomal membranes, like plasma membranes, restrict hydrophilic molecules but permit the passive diffusion of weakly basic lipophilic molecules through pH partitioning.^27,28^ Once entering the acidic lysosomal environment, these molecules undergo protonation, becoming hydrophilic, and effectively trapped within lysosomes. Based on this principle, we propose a strategy in which membrane receptor-targeting ligands (e.g., small molecules, peptides, aptamers, nanobodies, and antibodies) are conjugated with lysosome-localized molecules to enhance receptor degradation (Scheme 1a). Upon internalization, these lysosome-localized molecules undergo a pH-dependent shift to the hydrophilic form, reducing their ability to diffuse across the lysosomal membrane. This shift is expected to enhance the lysosomal retention of receptor–ligand complexes, preventing recycling and favoring degradation of receptors (Scheme 1b). This novel strategy offers a receptor-targeted degradation approach independent of cell surface lysosome-shuttling receptors, potentially overcoming limitations associated with cell-type dependence and tumor heterogeneity.

**Scheme 1:**
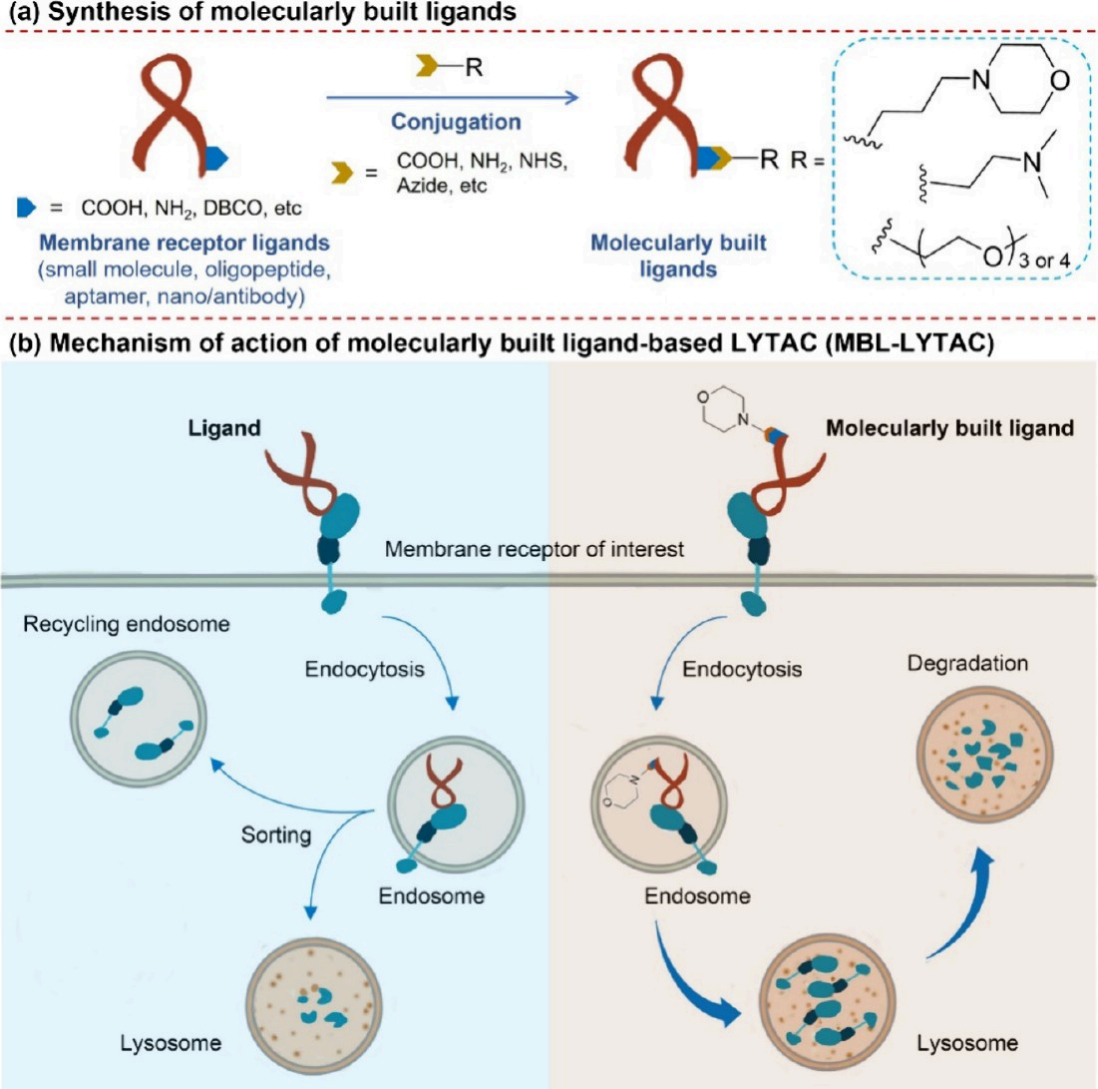
Schematic Diagram of Construction of Molecularly Built Ligands (a) and Mechanism of Action of Molecularly Built Ligand-Based LYTAC (MBL-LYTAC) (b)

## Results and Discussion

### Preparation and Functional Screening of Lysosome-Localized Molecules

Morpholine (MOR) is a lipophilic molecule with a p*K*_a_ range of 7.4–8.5, enabling its lysosomal localization via protonation in acidic environments.^29^ Due to its favorable properties and simple structure, morpholine was selected as a candidate lysosome-localized molecule for developing molecularly built ligands. The protonation of the amine group is influenced by the electron density on the nitrogen atom, making structural variations an important factor in optimizing lysosomal retention. To explore alternative structures with varying protonation properties, pyridine (PYD), imidazole (IMZ), dimethylethanamine (DEA), and piperidine (PID) were also considered as lysosome-localized candidates. These molecules exhibited distinct p*K*_a_ values due to the subtle structural differences (Figure 1a), influencing their behavior under acidic lysosomal conditions. Additionally, methoxy polyethylene glycols (mPEGs) were included in the candidate pool. mPEGs form hydrogen bonds with H_3_O^+^ in an acidic environment,^30,31^ potentially enhancing lysosomal entrapment. To assess the effect of polymer size on lysosomal localization, four mPEGs with polymerization degrees of 3, 4, 14, and 24 (mPEG_3_, mPEG_4_, mPEG_14_ and mPEG_24_) were selected.

**Figure 1:**
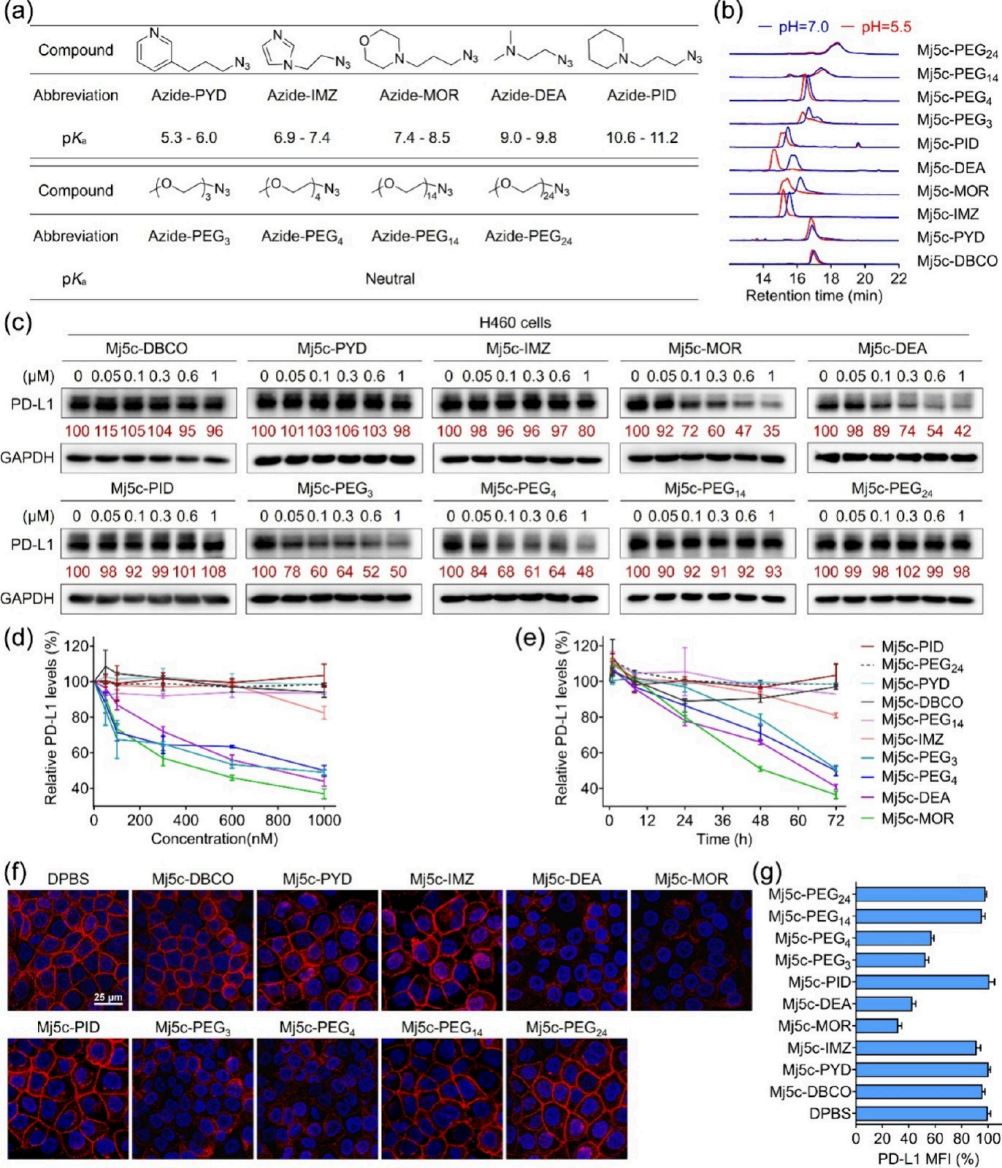
Preparation and functional screening of a molecularly built aptamer. (a) The structures, abbreviations, and p*K*_a_ values of the nine compound candidates. (b) The retention times of molecularly built aptamer candidates in reverse phase-HPLC at pH = 5.5 or 7.0. (c) WB analysis of PD-L1 levels in H460 cells treated with different molecularly built aptamer candidates for 72 h at different concentrations. Relative PD-L1 expression was determined via densitometry and normalized to the loading control. (d) Statistical analysis of PD-L1 levels in H460 cells receiving different treatments in (c). (e) The statistical analysis of PD-L1 levels in H460 cells treated with 1 μM molecularly built aptamer candidates for different incubation times, corresponding to the data shown in Figure S25. All data in (d) and (e) were presented as the mean ± SD, *n* = 2. (f) Immunofluorescence imaging analysis of PD-L1 levels on the cell surface after H460 cells were treated with 1 μM different molecularly built aptamer candidates for 72 h. Nuclei were stained by Hoechst 33258 (blue), and PD-L1 was indicated by Alexa Fluor 647-labeled antirabbit IgG (red). Scale bar = 25 μm. (g) Statistical analysis of the mean fluorescence intensity (MFI) indicating PD-L1 levels in (f). Data were presented as mean ± SD, *n* = 3.

To prepare molecularly built ligands, the nine candidate molecules were functionalized with azide groups (Figure 1a). Among them, azide-PYD, azide-IMZ, azide-MOR and azide-PID were synthesized (Figures S1–S4), while 2-azido-*N*,*N*-dimethylethanamine (azide-DEA), azide-PEG_3_, azide-PEG_4_, azide-PEG_14_, and azide-PEG_24_, were commercially available. The polymerization degrees of azide-PEG_14_ and azide-PEG_24_ were verified using MALDI-TOF-MS (Figures S5 and S6). Cytotoxicity analysis showed that all azide-functionalized compounds at 1 μM were biocompatible, with the exception of azide-PID and azide-IMZ (Figure S7). To further assess lysosomal integrity, fluorescence imaging using acridine orange was also performed. As shown in Figure S8, cells treated with all azide-functionalized compounds, except azide-PID, displayed a bright green fluorescence in the nuclei and a red fluorescence in lysosomes, indicating normal cellular function. Quantitative flow cytometric analysis confirmed that all azide-functionalized compounds caused slight or negligible lysosome cytotoxicity with the exception of azide-PID (Figure S9). Specifically, flow cytometry showed that only 4.82% cells exhibited lysosomal damage following azide-MOR treatment. However, 52.1% cells in the azide-PID-treated group showed significant lysosomal damage, as evidenced by a reduction in red fluorescence signals of acridine orange. Additionally, the slight increase in the expression levels of ALG2-interacting protein X (ALIX), a marker for lysosomal membrane damage repair, further supported the biocompatibility of azide-MOR (Figure S10).

To evaluate their lysosomal entrapment and protein degradation potential, PD-L1, an immune checkpoint protein that tumor cells exploit to evade immune surveillance,^32,33^ was chosen as a model protein. Nine PD-L1-targeted aptameric ligands were synthesized by conjugating the DBCO-labeled PD-L1-targeted aptamer Mj5c^34^ (Mj5c-DBCO, Table S1) with azide-functionalized compounds via a click reaction. Successful conjugation was confirmed by reverse-phase HPLC, mass spectrometry, or gel permeation chromatography (Figures S11–S24 and Table S2). Notably, conjugation with the aptamer markedly enhanced the biocompatibility of the azide-functionalized compounds, as demonstrated by reduced lysosomal damage and ALIX expression levels (Figures S9 and S10). The transition of these aptameric ligands toward hydrophilicity under acidic conditions was assessed using reverse-phase HPLC by analyzing retention times. As depicted in Figure 1b, Mj5c-PYD, Mj5c-PEG_14_, and Mj5c-PEG_24_ exhibited similar retention times at both pH 5.5 and pH 7.0, indicating minimal pH-dependent hydrophilicity shifts. In contrast, Mj5c-IMZ, Mj5c-MOR, Mj5c-DEA, Mj5c-PID, Mj5c-PEG_3_, and Mj5c-PEG_4_ displayed a shorter retention time at pH 5.5, suggesting increased hydrophilicity in acidic conditions.

The effect of the nine aptameric ligand candidates on PD-L1 levels in nonsmall cell lung cancer H460 cells was analyzed using Western blot (WB). A distinct pattern emerged among the candidates. Aptameric ligands prepared with azide-MOR, azide-DEA, azide-PEG_3_, or azide-PEG_4_ significantly reduced PD-L1 levels in a concentration- and time-dependent manner (Figures 1c–e and S25). For example, 1 μM Mj5c-MOR reduced ≈65% PD-L1 levels in H460 cells after 72 h, while Mj5c-IMZ displayed only a moderate effect, reducing PD-L1 expression by ≈20% under the same conditions. Conversely, Mj5c, Mj5c-DBCO, and the remaining four ligands (Mj5c-PID, Mj5c-PYD, Mj5c-PEG_14_, and Mj5c-PEG_24_) had negligible effects on PD-L1 levels, even at 1 μM (Figures 1c–e and S26). Immunofluorescence (IF) imaging further confirmed surface PD-L1 level decreases (Figure 1f,g). Overall, these results highlight the importance of lysosome-localized moieties in mediating PD-L1 degradation. The functional screening confirmed that the aptameric ligands could degrade PD-L1 by using morpholine, dimethylethanamine, or low-polymerized mPEG_3/4_ as a lysosome-localized moiety.

### The Lysosome-Localized Molecules Enhance the Internalization and Lysosomal Accumulation of the Aptamer-PD-L1 Complexes

To further explore the roles of lysosome-localized moieties in PD-L1 degradation, we assessed the binding affinity and internalization efficacy of the nine aptameric ligands. Flow cytometry analysis of H460 cells confirmed that all conjugated aptamers maintained equilibrium dissociation constants (*K*_d_’s) comparable to those of Mj5c-DBCO, indicating that lysosome-localized moieties did not compromise aptamer binding affinity (Figure S27). The internalization efficacy of these aptameric ligands was then evaluated by incubating them with H460 cells at 37 °C for 1, 8, and 24 h, followed by trypsin treatment to remove surface-bound ligands. Flow cytometric analysis revealed distinct internalization patterns among the candidates (Figure 2a,b). Compared to Mj5c-DBCO, Mj5c-PYD and Mj5c-IMZ displayed lower or comparable internalization levels, whereas Mj5c-MOR, Mj5c-DEA, Mj5c-PEG_3_, and Mj5c-PEG_4_ demonstrated significantly enhanced internalization across all time points, as evidenced by a marked increased geometric mean fluorescence intensity (GMFI) (Figure 2a,b). Notably, Mj5c-PID displayed only a modest increase in the level of internalization across all time points. Mj5c-PEG_14_ and Mj5c-PEG_24_ showed significant internalization enhancement only after 8-h incubation compared to Mj5c-DBCO.

**Figure 2:**
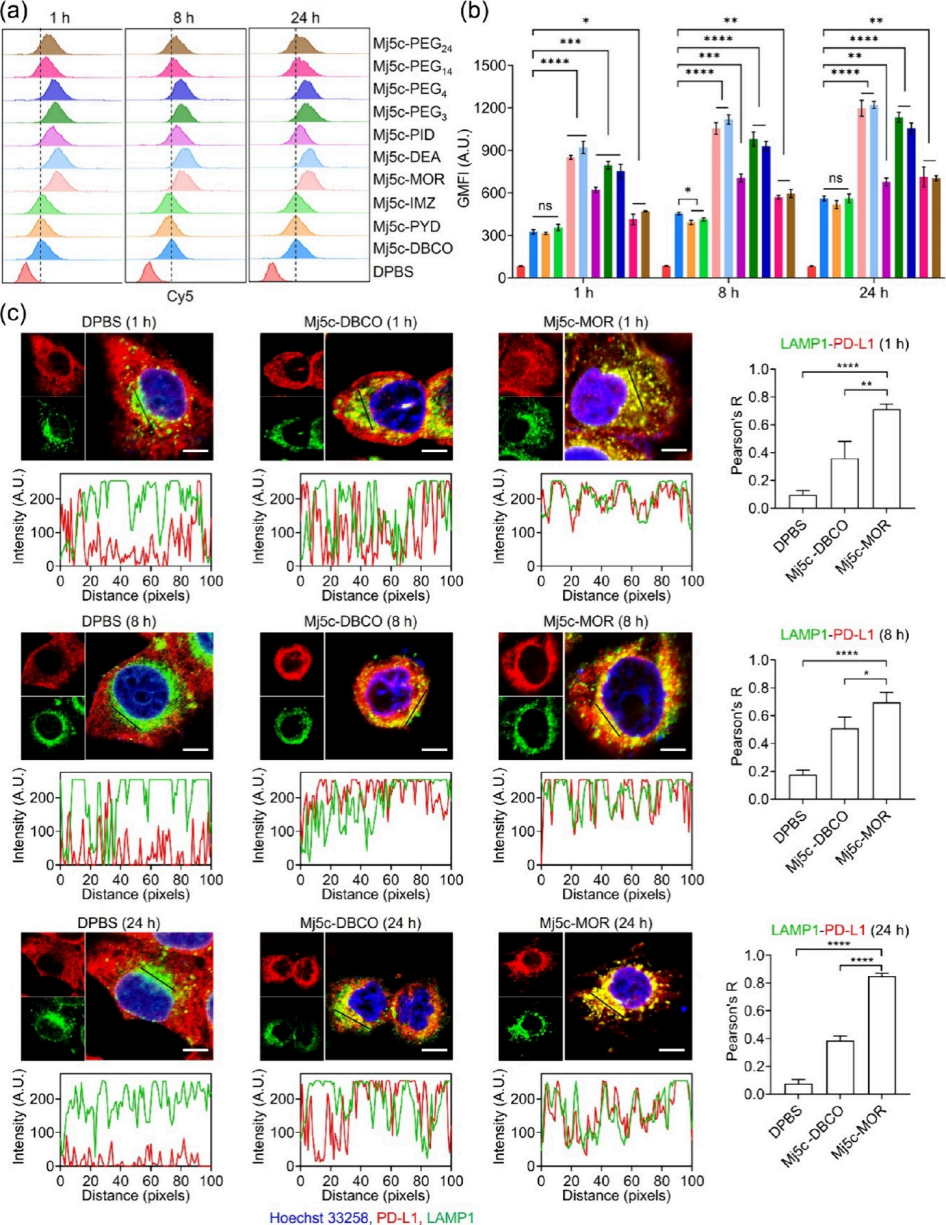
The effects of the nine compounds on the endocytosis and the distribution of aptamer-PD-L1 complexes. (a) Flow cytometric analysis of ligand internalization in H460 cells after cells were treated with 1 μM of different aptameric ligands at 37 °C for 1, 8, and 24 h, followed by trypsin treatment. (b) Statistical analysis of ligand internalization. The GMFI from panel (a) was used to quantify ligand internalization in H460 cells. Data were presented as mean ± SD, *n* = 3. (c) Colocalization analysis of PD-L1 and LAMP1 in H460 cells after cells were treated with 1 μM of Mj5c-DBCO or Mj5c-MOR for 1, 8, and 24 h, with DPBS as the control. Nuclei were stained by Hoechst 33258 (blue), PD-L1 was detected by Alexa Fluor594-labeled secondary antibody (red), and LAMP1 was detected by Alexa Fluor 488-labeled secondary antibody (green). The intensity profiles below the images displayed the fluorescence intensity of PD-L1 and LAMP1 along the black line. The overlay of red and green fluorescence indicated the colocalization of PD-L1 and LAMP1. The right panels showed the statistical analysis of the colocalization factor, represented by Pearson’s correlation coefficient (*R*). Data were presented as mean ± SD, based on three independent experimental replicates. Statistical significance was determined by one-way ANOVA with a Tukey post hoc test. ns: no significance, *P < 0.05, **P < 0.01, ***P < 0.001, and ****P < 0.0001. Scale bar = 10 μm.

To further characterize the intracellular fate of the internalized aptameric ligands, we conducted a colocalization analysis using Lysotracker. As shown in Figure S28, the internalization of Mj5c-MOR, Mj5c-DEA, Mj5c-PID, Mj5c-PEG_3_ and Mj5c-PEG_4_, was notably higher compared to Mj5c-DBCO, Mj5c-PYD, Mj5c-IMZ, PEG_14_, and Mj5c-PEG_24_, as evidenced by the intracellular red fluorescence. Co-localization analysis further revealed that Mj5c-MOR, Mj5c-DEA, Mj5c-PEG_3_, and Mj5c-PEG_4_ predominantly accumulated in lysosomes, as indicated by strong overlap with Lysotracker. In contrast, although Mj5c-PID displayed high internalization, it caused lysosomal disruption, leading to a reduction in its retention within the lysosomes. These findings not only confirm the internalization pattern but also provide insights into the differential lysosomal distribution of aptameric ligands.

These results indicate that the physicochemical properties of the conjugated moieties critically shape aptamer internalization and lysosomal trafficking. Conjugates containing pyridine or imidazole, with lower p*K*_a_ values, showed reduced or unchanged internalization compared with Mj5c-DBCO. In contrast, conjugates containing morpholine, dimethylethanamine or piperidine, with higher p*K*_a_ values, enhanced uptake. This difference is likely due to the positive charge on these moieties, which strengthens the electrostatic attraction between the ligands and the negatively charged cell membrane. Once internalized, Mj5c-MOR and Mj5c-DEA accumulated effectively in lysosomes, likely due to the protonation of morpholine and dimethylethanamine, which promotes their retention. By contrast, Mj5c-PID, containing the more alkaline piperidine group, appeared to disrupt the lysosomal function, thereby reducing lysosomal accumulation.

Conjugation with mPEG markedly affected ligand trafficking. Conjugates containing shorter chains, such as Mj5c-PEG_3_ and Mj5c-PEG_4_, exhibited effective internalization with partial plasma membrane retention. While conjugates containing long chains, such as Mj5c-PEG_14_ and Mj5c-PEG_24_, remained predominantly on the cell surface, highlighting the importance of the PEG chain length. Among the aptamer-PEG conjugates, Mj5c-PEG_3/4_ displayed enhanced lysosomal accumulation and retention, likely due to increased hydrophilicity by formation of hydrogen bonds with hydronium ions in the lysosomes. Collectively, these findings identify morpholine, dimethylethanamine, and low-molecular-weight mPEG_3/4_ as the most effective modifications for promoting both internalization and lysosomal localization, thereby supporting receptor degradation.

To further link lysosomal localization with PD-L1 degradation, we examined colocalization with lysosomal-associated membrane protein 1 (LAMP1).^35^ In DPBS-treated H460 cells, PD-L1 exhibited a diffuse intracellular distribution with minimal overlap with LAMP1 (Figure 2c). Treatment with Mj5c-DBCO induced PD-L1 internalization and moderate colocalization with LAMP1. In contrast, Mj5c-PYD, Mj5c-IMZ, Mj5c-PEG_14_, Mj5c-PEG_24_, and Mj5c-PID did not significantly increase in PD-L1-LAMP1 colocalization relative to Mj5c-DBCO over a 24-h period, as evidenced by fluorescence imaging and Pearson’s correlation analyses (Figures 2c and S29 and 30). Notably, Mj5c-MOR, Mj5c-DEA, Mj5c-PEG_3_, and Mj5c-PEG_4_ induced clear colocalization at 8 h, which became pronounced by 24 h, evident from the stronger yellow fluorescence in merged images and higher Pearson’s coefficients. These findings suggest that PD-L1 progressively accumulates in lysosomes following treatment with these conjugates, likely reflecting their enhanced capacity to promote internalization and lysosomal trafficking.

### Mechanism of Action of Molecularly Built Ligand-LYTACs (MBL-LYTACs)

Given its robust ability to induce PD-L1 degradation, Mj5c-MOR was selected as a representative MBL-LYTAC for the mechanistic studies. Proteomic analysis confirmed that Mj5c-MOR selectively reduced PD-L1 levels (Figure 3a). As a control, Ctrl-DNA1-MOR, a nontargeted aptamer conjugate,^34^ had no effect (Tables S1 and S2 and Figures S21, S31), confirming that degradation strictly depends on ligand-PD-L1 recognition. Similarly, a 1:1 mixture of Mj5c and azide-MOR, Azide-MOR, or Mj5c-DBCO failed to induce PD-L1 degradation (Figures S32 and S33a), excluding nonspecific effects. To assess whether the triazole linkage from DBCO-azide conjugation contributed to activity, Mj5c­(Amide)-MOR was synthesized using an amide bond (Tables S1, S2). This variant retained comparable degradation activity (Figure 3b), confirming that the Mj5c-MOR function is independent of linker chemistry. Further enhancement of serum stability was achieved by incorporating two inverted deoxythymidines at the 3′-terminus (Table S1 and Figure S33b), which improved degradation efficiency in both H460 and 4T1 cells without altering Mj5c’s intrinsic activity (Figures S33a and S34).

**Figure 3:**
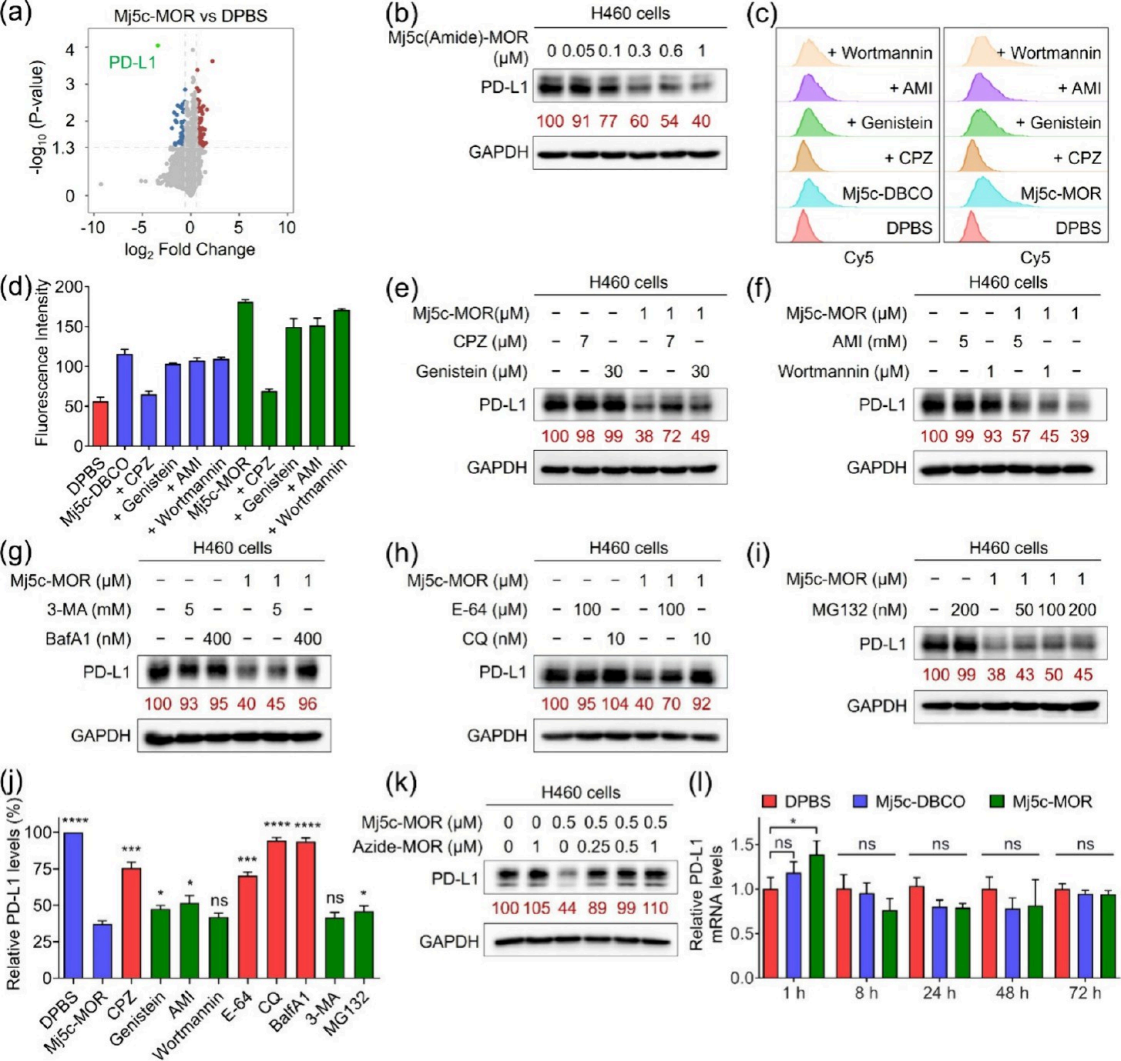
Mechanism of action of molecularly built aptamer Mj5c-MOR. (a) Differential proteomic analysis of H460 cells treated with Mj5c-MOR compared to those treated with DPBS. Each condition was tested in triplicates, and statistical significance was determined using an unpaired *t*-test. Proteins with *P*-values ≤ 0.05 and |log_2_ Fold Change| ≥ 0.58 were considered significantly altered. (b) WB analysis of PD-L1 levels in H460 cells treated with Mj5c­(Amide)-MOR at different concentrations for 72 h. (c) Flow cytometry analysis of the effects of different endocytosis inhibitors on the endocytosis of 1 μM Cy5-labeled Mj5c-DBCO or Mj5c-MOR in H460 cells at 37 °C. Incubation time: 8 h. Chlorpromazine (CPZ): 7 μM, genistein: 30 μM, amiloride (AMI): 5 mM, and wortmannin: 1 μM. (d) Statistical data of GMFI in panel (c). Data are presented as mean ± SD, *n* = 3. (e–i) WB analysis of PD-L1 levels in H460 cells after cells were incubated with 1 μM Mj5c-MOR for 72 h in the absence or presence of various inhibitors, including bafilomycin A1 (BafA1, 400 nM), chloroquine (CQ, 10 nM), E-64 (100 μM), 3-methyladenine (3-MA, 5 mM), MG132 (50, 100, and 200 nM). (j) Statistic data of PD-L1 levels in H460 cells treated with 1 μM Mj5c-MOR in the presence or the absence of various inhibitors. Data were from panels (e–i). All data were presented as mean ± SD, *n* = 2. (k) WB analysis of PD-L1 levels in H460 cells incubated with 0.5 μM Mj5c-MOR in the absence or presence of varying concentrations of azide-MOR (0.25 μM, 0.5 μM, or 1 μM). In panels (b, e–i, and k), relative PD-L1 expression was determined via densitometry and normalized to the loading control. (l) RT-qPCR analysis of PD-L1 gene levels in H460 cells at 1 h, 8 h, 24 h, 48 h, and 72 h postincubation with DPBS, 1 μM Mj5c-DBCO or Mj5c-MOR. All data were presented as mean ± SD, *n* = 4. Statistical significance was determined by one-way ANOVA with a Tukey post hoc test, ns: no significance, *P < 0.05, **P < 0.01, ***P < 0.001, ****P < 0.0001.

To dissect the mechanism of action of Mj5c-MOR-induced PD-L1 degradation, we evaluated its sensitivity to various small-molecule regulators. Endocytosis inhibitors included chlorpromazine (CPZ, inhibiting clathrin-mediated endocytosis),^36^ genistein (inhibiting caveolae-mediated endocytosis),^37^ amiloride (AMI, inhibiting macropinocytosis by lowering the submembranous pH and preventing the activation of Rac1 and Cdc42 signaling),^38^ and wortmannin (blocking macropinocytosis by inhibiting phosphatidylinositol-3 kinase).^39^ Lysosomal inhibitors included bafilomycin A1 (BafA1, a V-ATPase inhibitor blocking lysosomal proton transport),^40^ chloroquine (CQ, raising intralysosomal pH),^41^ and E64 (inhibiting cathepsin B).^24^ Additionally, the autophagy inhibitor 3-methyladenine (3-MA)^42^ and proteasomal inhibitor MG132 ^43^ were tested. PD-L1 endocytosis induced by Mj5c-MOR followed the clathrin-mediated pathway,^44^ as CPZ, but not genistein, AMI, or wortmannin, significantly blocked internalization (Figure 3c,d) and degradation (Figure 3e,f). For example, in H460 cells treated with 1 μM Mj5c-MOR for 72 h, PD-L1 levels decreased to ≈39%. Inhibitor cotreatment increased PD-L1 levels to ≈72% (CPZ), ≈49% (genistein), ≈57% (AMI), and ≈45% (wortmannin), confirming the predominant role of clathrin-mediated endocytosis. Autophagy inhibition with 3-MA produced only a marginal effect (Figure 3g), indicating that Mj5c-MOR-induced PD-L1 degradation is not autophagy-dependent.

Next, we explored the degradation route. Lysosomal inhibitors substantially inhibited Mj5c-MOR-induced PD-L1 degradation, whereas MG132 had little effect (Figure 3g–i), demonstrating that degradation is lysosome-dependent rather than proteasome-mediated. Among lysosomal inhibitors, BafA1 and CQ, both disrupting lysosomal acidification, were more effective than cathepsin B-specific inhibitor E64, highlighting the importance of lysosomal pH in degradation efficiency (Figure 3j). Thus, both clathrin-mediated endocytosis and lysosomal acidification are critical for Mj5c-MOR function.

The role of lysosome-localized moieties was further examined by coincubating cells with azide-MOR or azide-PEG_3_. Both reagents abolished PD-L1 degradation induced by their respective molecularly built ligands (Figures 3k and S35), likely because these lysosome-localized molecules diffuse readily into cells and sequester H_3_O^+^, thereby reducing ligand retention. These findings emphasize that lysosome-localized molecules are indispensable for sustaining the degradation activity of the MBL-LYTACs.

Finally, RT-qPCR analysis showed that neither Mj5c-MOR nor Mj5c-DBCO affected PD-L1 mRNA levels over 72 h compared to DPBS (Figure 3l). This confirms that Mj5c-MOR-induced PD-L1 degradation post-transcriptionally rather than by transcriptional repression. Collectively, these results collectively demonstrate that Mj5c-MOR promotes PD-L1 degradation via a lysosomal pathway.

### Universality Analysis of MBL-LYTACs

To evaluate the universality of MBL-LYTACs, Sgc8-MOR, a molecularly built ligand targeting protein tyrosine kinase 7 (PTK7), was first prepared by conjugating DBCO-labeled aptamer Sgc8 ^45^ (Sgc8-DBCO) with azide-MOR (Tables S1, S2 and Figure S22). A nontargeted control ligand, Ctrl-DNA2-MOR, was prepared using a PTK7-nontargeted control DNA 2 ^46^ (Ctrl-DNA2) (Tables S1, S2 and Figure S23). Both Sgc8-MOR and Sgc8-DBCO bound HCT116 colon cancer cells with a comparable affinity (Figure S36a); however, Sgc8-MOR induced PTK7 degradation in a dose- and time-dependent manner with significantly greater efficiency (Figures 4a,b and S36b). For example, at 100 nM, Sgc8-MOR reduced PTK7 expression by ≈70%, compared with ≈20% by Sgc8-DBCO. Ctrl-DNA2-MOR had no effect, confirming the specificity of Sgc8-MOR (Figure S37).

**Figure 4:**
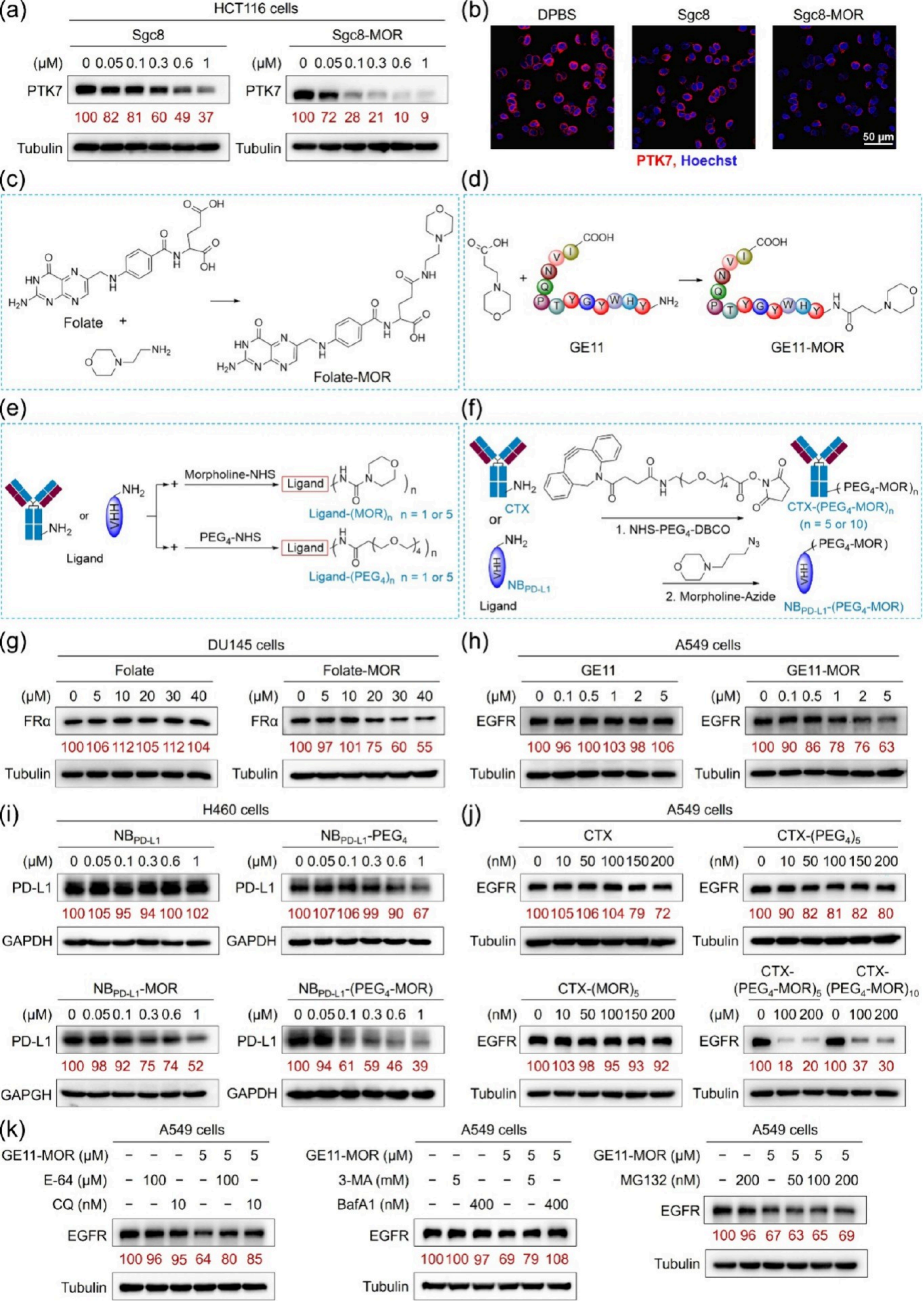
Universality analysis of molecularly built ligands. (a) WB analysis of PTK7 levels in HCT116 cells treated with Sgc8-DBCO or Sgc8-MOR at different concentrations for 72 h. (b) Immunofluorescence imaging analysis of PTK7 levels on the surface after HCT116 cells were treated with 100 nM Sgc8-DBCO or Sgc8-MOR for 72 h. Nuclei were stained by Hoechst 33258 (blue), and PTK7 was stained by Alexa Fluor 647-labeled antirabbit IgG (red). Scale bar = 50 μm. (c–f) Synthesis diagram of FA-MOR (c), GE11-MOR (d), nanobody/antibody-(MOR)_*n*_ and nanobody/antibody-(PEG_4_)_n_, *n* = 1 or 5 (e), and nanobody/antibody-(PEG_4_-MOR)_n_, *n* = 1, 5, or 10 (f). (g) WB analysis of FRα levels in DU145 cells treated with FA or FA-MOR at different concentrations for 72 h. (h) WB analysis of EGFR levels in A549 cells treated with GE11 or GE11-MOR at different concentrations for 72 h. (i) WB analysis of PD-L1 levels in H460 cells treated with NB_PD‑L1_, NB_PD‑L1_-PEG_4_, NB_PD‑L1_-MOR, or NB_PD‑L1_-(PEG_4_-MOR) at different concentrations for 72 h. (j) WB analysis of EGFR levels in A549 cells treated with CTX, CTX-(PEG_4_)_5_, CTX-(MOR)_5_, CTX-(PEG_4_-MOR)_5_, or CTX-(PEG_4_-MOR)_10_ at different concentrations for 48 h. (k) WB analysis of EGFR levels in A549 cells after cells were incubated with 5 μM GE11-MOR for 72 h in the absence or presence of various inhibitors, including BafA1 (400 nM), CQ (10 nM), E-64 (100 μM), 3-MA (5 mM), and MG132 (50, 100, or 200 nM). In panels (a) and (g–k), relative target protein expression was determined via densitometry and normalized to the loading control.

To test whether lysosome-localized moieties such as morpholine can endow other ligands with degradative capacity, we extended this strategy to a range of receptor-targeted ligands, including folate (FA), a PD-L1 nanobody^47^ (NB_PD‑L1_, Table S3), an EGFR-targeted oligopeptide GE11 ^48^ (Table S3), and antibodies such as Cetuximab^6^ (CTX) and Atezolizumab (Atz) (Table S4). These ligands varied in composition, structure, and molecular weight from ≈440 Da to ≈145 kDa.

Molecularly built constructs (FA-MOR and GE11-MOR, and control HW12-MOR) were synthesized by a condensation reaction (Figures 4c,d and S38, S39, and Table S3). Nanobody- and antibody-based MBL-LYTACs were generated by conjugating MOR-NHS, NHS-PEG_4_, or NHS-PEG_4_-DBCO with azide-MOR, yielding NB_PD‑L1_-MOR, NB_PD‑L1_-PEG_4_, NB_PD‑L1_-(PEG_4_-MOR), CTX-(MOR)_5_, CTX-(PEG_4_)_5_, CTX-(PEG_4_-MOR)_5_, and CTX-(PEG_4_-MOR)_10_ (Figure 4e,f). The number of lysosome-localized molecules in these ligands was tuned by varying nanobody/antibody-to-MOR ratios (Figure S40). Importantly, competitive binding analysis confirmed that chemical modifications did not compromise the ligand binding specificity (Figure S41).

Functional analysis revealed that MBL-LYTACs consistently enhanced the degradation of their target receptors. For instance, FA-MOR (40 μM) reduced folate receptor α (FRα) levels by ≈45% after 72 h, whereas FA alone had no effect (Figure 4g). The activity was blocked by free FA, confirming specificity (Figure S42). Similar, GE11-MOR, but not GE11, induced EGFR degradation in A549 cells, despite a comparable binding affinity (Figures 4h and S43). Control ligands HW12 and HW12-MOR showed no effect (Figure S44). Likewise, NB_PD‑L1_ required MOR conjugation to achieve degradation: after 72 h, 1 μM NB_PD‑L1_-PEG_4_, NB_PD‑L1_-MOR, and NB_PD‑L1_-(PEG_4_-MOR) reduced PD-L1 levels by ≈33%, ≈48%, and ≈61%, respectively, in H460 cells (Figure 4i).

Molecularly built antibodies CTX-(PEG_4_-MOR)_*n*_ (*n* = 5 or 10) also demonstrated strong activity. At 100 nM, CTX-(PEG_4_-MOR)_5_ and CTX-(PEG_4_-MOR)_10_ reduced EGFR levels by ≈82% and ≈63%, respectively, whereas unmodified CTX alone had no effect in A549 cells (Figure 4j). In contrast, CTX-(PEG_4_)_5_ and CTX-(MOR)_5_, lacking a flexible linker, exhibited reduced activity, likely due to steric hindrance masking lysosome-localized groups (Figure 4j). Atezolizumab-based constructs (Atz-(PEG_4_-MOR)_5_ similarly induced effective PD-L1 degradation in H460 (Figure S45). Across multiple ligands, aptamers, small molecules, peptides, nanobodies, and antibodies, MBL-LYTACs successfully degraded their corresponding membrane receptors in diverse cell types (Figure S46), underscoring the broad applicability of this platform.

Mechanistic studies of GE11-MOR further validated this universality. GE11-MOR followed the same clathrin-mediated internalization pathway as GE11, but with enhanced uptake and lysosomal accumulation of EGFR (Figures S47 and S48a,b). EGFR degradation was abolished by CPZ and wortmannin, but not by genistein, AMI, and 3-MA (Figure S48c,d), confirming dependence on clathrin-mediated endocytosis^49^ and PI3K-dependent vesicle formation.^50^ Lysosomal inhibitors (CQ and BafA1) blocked degradation more effectively than E64, while MG132 had minimal effect (Figure 4k), demonstrating lysosomal, but not proteasomal, dependence. EGFR mRNA levels in A549 cells treated with GE11-MOR remained unchanged compared with DPBS-treated cells (Figure S49), confirming a post-transcriptional degradation mechanism. Notably, GE11-MOR, but not GE11, blocked the EGF-induced phosphorylation of EGFR and downstream AKT (Figure S50), indicating suppression of receptor signaling. Together, these results establish that MBL-LYTACs are a versatile and programmable strategy, capable of adapting diverse ligands to promote selective, lysosome-dependent degradation of membrane proteins, thereby offering a broadly applicable platform for therapeutic protein regulation.

### Mj5cT_inv_-MOR Inhibits 4T1 Syngeneic Tumor Growth via Degrading PD-L1

The interaction between PD-L1 on tumor cells and PD-1 on T cells suppresses T cell-mediated cancer cell killing.^33^ To assess whether Mj5cT_inv_-MOR, designed by flanking two inverted deoxythymidines at the 3′-terminus of Mj5c-MOR to enhance its serum stability (Table S1), could enhance T cell cytotoxicity, H460 cells were incubated with 1 μM Mj5cT_inv_-MOR or control agents for 72 h, followed by a 2 h incubation with T cells. The cells were then analyzed using propidium iodide (PI) staining and flow cytometric analysis. Figure S51a revealed a higher percentage of PI-stained dead H460 cells (6.88%) in the Mj5cT_inv_-MOR plus T cell group compared to Mj5cT_inv_-DBCO and other controls. Fluorescence micrographs also confirmed these findings, showing a greater number of red-fluorescence dead H460 cells in the Mj5cT_inv_-MOR plus T cell group (Figure S51b,c). Extending T cell incubation to 24 h increased interferon-γ (IFN-γ) levels to ≈143 pg/mL in Mj5cT_inv_-MOR-treated cells compared to controls (Figure S52d). These findings suggest that Mj5cT_inv_-MOR effectively enhances T cell-mediated cancer cell killing through PD-L1 degradation.

The immunogenicity of Mj5cT_inv_-MOR was evaluated in immunocompetent, healthy BALB/c female mice, comparing it to anti-PD-L1 antibody (a-PD-L1, Table S4). Systemic administration of a-PD-L1 increased serum tumor necrosis factor-α (TNF-α), interleukin-6 (IL-6), and interferon-β (IFN-β) levels in a dose-dependent manner (Figure 5a–c), with 140 μg causing a >2-fold increase compared to DPBS-treated mice. Blood tests showed that a-PD-L1 altered several parameters, including white blood cells, lymphocytes, neutrophils, monocytes, alanine aminotransferase, and aspartate aminotransferase (Figure S52). In contrast, Mj5cT_inv_-MOR induced only marginal cytokine changes (Figure 5a–c), and blood tests confirmed its favorable biosafety (Figure S52), suggesting immunogenicity that is lower than that of a-PD-L1.

**Figure 5:**
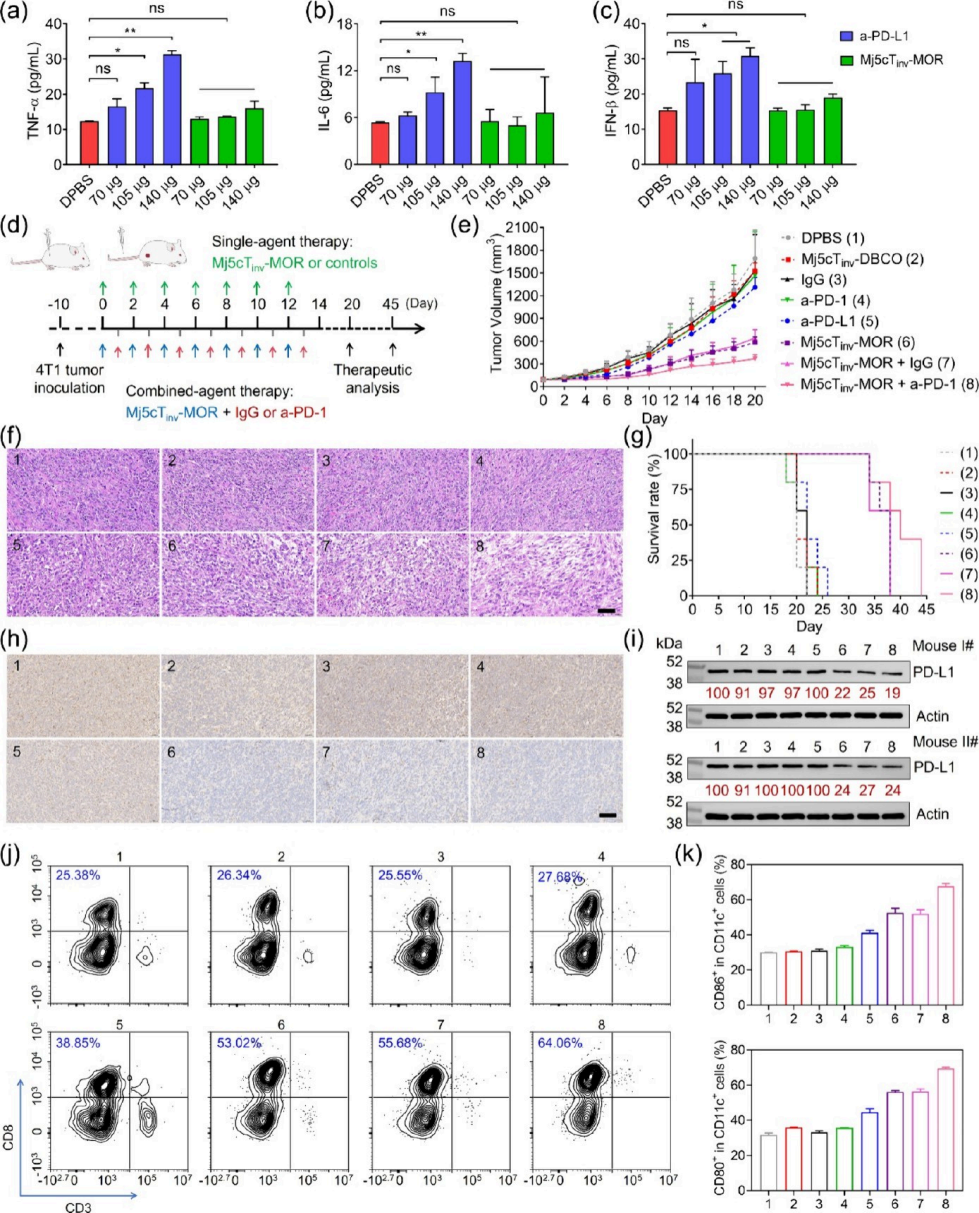
Therapeutic efficacy analysis of Mj5cT_inv_-MOR in 4T1 syngeneic breast tumor model. (a–c) The levels of TNF-α (a), IL-6 (b), IFN-β (c) in the serum of healthy BALB/c female mice at 24 h post injection of a-PD-L1 or Mj5cT_inv_-MOR at different doses via the tail vein, as determined by ELISA. (d) Schematic illustration of 4T1 xenograft tumor model establishment and treatment regimen. (e) Growth curves of 4T1 tumors in mice receiving different treatments. All data were presented as mean ± SD, *n* = 5. (f) H&E staining analysis of 4T1 tumors receiving different treatments after treatment. Scale bar = 50 μm. (g) Survival rate analysis of mice receiving different treatments (*n* = 5). (h-i) Immunohistochemical staining analysis (h) and WB analysis (i) of PD-L1 levels in tumors from mice receiving different treatments after treatment. Scale bar = 100 μm. Relative PD-L1 expression was determined via densitometry and normalized to the loading control. (j) Flow cytometric assay of CD8^+^ T cells from the tumors. Cells were stained with APC-labeled anti-CD3 antibody and PE-labeled anti-CD8 antibody. (k) Statistical analysis of the number of CD86^+^CD11c^+^ and CD80^+^CD11c^+^ cell populations in the draining lymph nodes, corresponding to the data in Figure S54. Statistical significance was determined by one-way ANOVA with a Tukey post hoc test, ns: no significance, *P < 0.05, **P < 0.01, ***P < 0.001, ****P < 0.0001.

We then investigated the pharmacokinetics and tumor-targeting ability of the Mj5cT_inv_-MOR. Figures S53, S54 demonstrated that Mj5cT_inv_-MOR exhibited a comparable half-life in healthy BALB/c female mice to Mj5cT_inv_-DBCO but accumulated more effectively in 4T1 tumors. These results suggest that MOR conjugation does not accelerate clearance from circulation—a common issue with positively charged molecules—but instead enhanced tumor accumulation, likely due to increased lysosomal retention.^51^

Next, the therapeutic efficacy of Mj5cT_inv_-MOR was evaluated in a 4T1 syngeneic breast tumor mouse model. The tumor-bearing mice were divided into 8 groups (*n* = 5) and intravenously injected with DPBS, Mj5cT_inv_-DBCO, immunoglobulin (IgG, Table S4), anti-PD-1 antibody (a-PD-1, Table S4), a-PD-L1, Mj5cT_inv_-MOR, Mj5cT_inv_-MOR plus IgG, or Mj5cT_inv_-MOR plus a-PD-1. The treatment regimen began on day 0, with single-agent treatments (105 μg per mouse) administered every 2 days for 7 doses, while combination treatments alternated Mj5cT_inv_-MOR with antibody/IgG (Figure 5d). In the DPBS-treated groups, tumors grew rapidly from ≈100 to ≈1700 mm^3^ by day 20. Compared to IgG, both a-PD-L1 and a-PD-1 had limited effects on 4T1 tumor growth, likely due to 4T1 tumor resistance^52,53^ (Figures 5e and S55a,b). Mj5cT_inv_-DBCO was ineffective, whereas Mj5cT_inv_-MOR significantly reduced tumor size (600 mm^3^ vs 1500 mm^3^). Combining Mj5cT_inv_-MOR with a-PD-1, but not IgG, further improved the therapeutic efficacy, reducing the tumor size to ≈370 mm^3^ by day 20 (Figure 5e). H&E staining showed greater tumor apoptosis in groups involving Mj5cT_inv_-MOR (Figure 5f). Mice treated with Mj5cT_inv_-MOR alone or in combination had median survival times of 38–40 days, compared to 20–22 days in the control groups, further confirming its therapeutic potential (Figure 5g).

At the molecular level, WB analysis and immunohistochemical staining revealed that Mj5cT_inv_-DBCO, IgG, a-PD-L1, or a-PD-1 had minimal effect on PD-L1 expression, while Mj5cT_inv_-MOR significantly reduced PD-L1 levels in tumors (Figure 5h,i). The impact of the PD-L1 degradation on the tumor immune microenvironment was assessed by analyzing CD8^+^ cytotoxic T cells (CTLs) in tumors and mature dendritic cells (DCs) in draining lymph nodes. As shown in Figure 5j, mice treated with Mj5cT_inv_-MOR, Mj5cT_inv_-MOR plus IgG, and Mj5cT_inv_-MOR plus a-PD-1 had significantly higher CD8^+^ CTLs (≈53.02%, ≈55.68% and ≈64.06%, respectively) than DPBS (≈25.38%), Mj5cT_inv_-DBCO (≈26.34%), IgG (≈25.55%), a-PD-1 (≈27.68%) or a-PD-L1 (≈38.85%) groups. A similar trend was observed in the CD80^+^CD11c^+^ and CD86^+^CD11c^+^ DC populations in the draining lymph nodes (Figures 5k and S56). These results suggest that Mj5cT_inv_-MOR enhances the infiltration of CTLs and promotes the maturation of the DCs. After 21-day treatment, Mj5cT_inv_-MOR plus a-PD-1 resulted in higher serum TNF-α, IFN-β and IL-6 levels compared to Mj5cT_inv_-MOR alone or in combination with IgG (Figure S57).

No significant weight changes were observed across treatment groups (Figure S55c). H&E staining of normal organs, blood routine test, and biochemical analysis confirmed that the treatment involving Mj5cT_inv_-MOR caused negligible effect on mice (Figures S58, S59). However, a-PD-L1 caused damage to the heart and liver (Figure S59).^54^ In conclusion, Mj5cT_inv_-MOR, a molecularly built aptameric ligand, effectively degrades PD-L1, enhances T cell-mediated cancer cell killing, and improves survival in immunosuppressive 4T1 tumor model with minimal side effects. Additionally, it synergizes with a-PD-1 to boost antitumor immune responses, positioning it as a promising candidate for cancer immunotherapy.

### Molecularly Built Cetuximab CTX-(PEG_4_-MOR)_5_ more Efficiently Inhibits A549 Xenograft Tumor Growth than CTX by Degrading EGFR

In contrast to CTX, which inhibits the EGF-EGFR interaction,^6^ CTX-(PEG_4_-MOR)_5_ efficiently reduced EGFR levels, resulting in a significant decrease in phosphorylated EGFR (p-EGFR) and AKT (p-AKT) in response to EGF stimulation (Figure 6a). The effect was dose-dependent. For example, at 100 nM, CTX-(PEG_4_-MOR)_5_ degraded ≈49% of EGFR and reduced p-EGFR by ≈62% and p-AKT by ≈22%, following stimulation with 50 ng of EGF. The findings highlight the strong potential of CTX-(PEG_4_-MOR)_5_ as a tumor-inhibiting agent through EGFR degradation.

**Figure 6:**
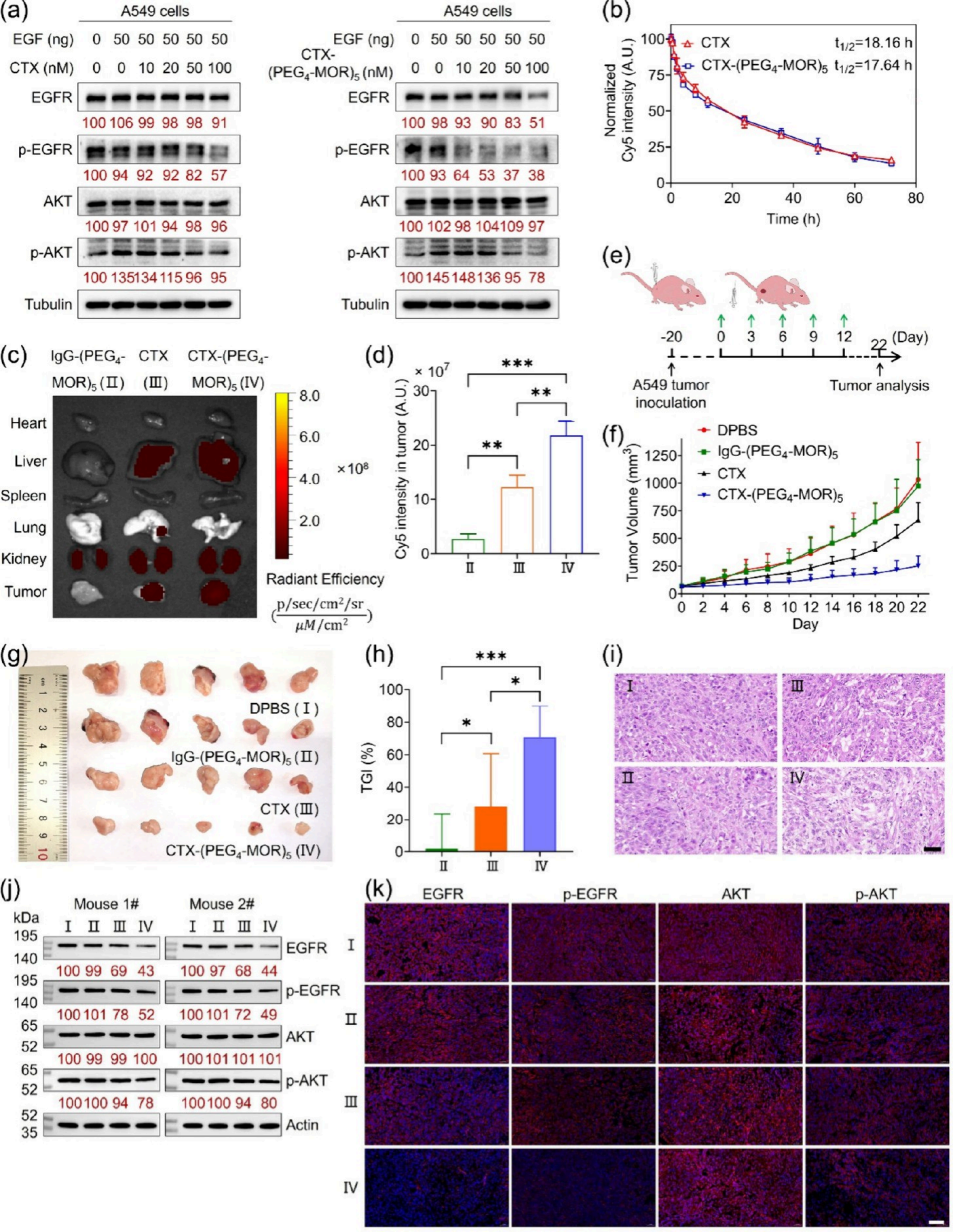
Therapeutic efficacy analysis of CTX-(PEG_**4**_-MOR)_**5**_in A549 xenograft tumor model. (a) WB analysis of the levels of EGFR, p-EGFR, AKT, and p-AKT in A549 cells, which were pretreated with or without 50 ng of EGFR for 2 h, followed by incubation with CTX or CTX-(PEG_4_-MOR)_5_ at different concentrations for 48 h. (b) In vivo circulation half-life of CTX and CTX-(PEG_4_-MOR)_5_ after 80 μg of Cy5-labled antibodies were injected into healthy BALB/c female nude mice via tail vein. All data were presented as mean ± SD, *n* = 3. (c) Fluorescence imaging of main organs and tumors excised from A549 tumor-bearing mice at 48 h points post injection of 80 μg Cy5-labeled IgG-(PEG_4_-MOR)_5_, CTX, and CTX-(PEG_4_-MOR)_5_, respectively. (d) Quantitative statistics of fluorescence intensity in tumors in panel (c). All data were presented as mean ± SD, *n* = 3. (e) Schematic illustration of the A549 xenograft tumor model establishment and treatment regimen. (f) Tumor growth curves in mice receiving different treatments. All data were presented as mean ± SD, *n* = 5. (g) Tumor photograph from different groups after treatment. (h) Tumor growth inhibition rate of CTX, IgG-(PEG_4_-MOR)_5_ and CTX-(PEG_4_-MOR)_5_ on A549-tumor bearing mice. (i) H&E staining of A549 tumors from different groups after treatment. Scale bar: 50 μm. (j, k) WB analysis (j) and immunofluorescence imaging analysis (k) of the levels of EGFR, p-EGFR, AKT and p-AKT in tumors after treatment. Scale bar = 100 μm. Nuclei were stained by DAPI (blue), and the proteins were indicated by Alexa Fluor 647-labeled antirabbit secondary antibody (red). In panels (a, j), relative target protein expression was determined via densitometry and normalized to the loading control. Statistical significance was determined by one-way ANOVA with a Tukey post hoc test. ns: no significance, *P < 0.05, **P < 0.01, ***P < 0.001, and ****P < 0.0001.

To evaluate therapeutic efficacy, we first examined the impact of molecular engineering on the targeting ability and pharmacokinetics of CTX. In vivo fluorescence imaging demonstrated that both CTX and CTX-(PEG_4_-Cy5)_5_ successfully targeted EGFR-expressing A549 tumors (Figure S60). As shown in Figure 6b, conjugation with MOR did not alter the circulation half-life of CTX in healthy BALB/c nude mice, indicating that the pharmacokinetic profile was preserved. Notably, ≈80% CTX-(PEG_4_-Cy5)_5_ (with Cy5 replacing MOR) remained intact after 72 h (Figure S61). Furthermore, Figure 6c,d revealed that CTX-(PEG_4_-MOR)_5_ accumulated more efficiently in tumor compared to unmodified CTX, likely due to enhanced lysosomal retention.^51^ These results confirm the compatibility and stability of the molecular engineering.

Then, the therapeutic efficacy of CTX-(PEG_4_-MOR)_5_ was evaluated by intravenously injecting 80 μg of CTX-(PEG_4_-MOR)_5_, CTX, or IgG-(PEG_4_-MOR)_5_ into A549 tumor-bearing mice every 3 days for 5 doses (Figure 6e), with DPBS-treated mice as the control. Treatment began on day 0. By day 22, tumors in the DPBS-treated groups grew to ≈1000 mm^3^, while IgG-(PEG_4_-MOR)_5_ displayed negligible tumor inhibition (≈980 mm^3^) (Figure 6e,f). CTX treatment led to a tumor growth inhibition (TGI) rate of ≈35%, whereas CTX-(PEG_4_-MOR)_5_ significantly suppressed A549 tumor growth with a TGI rate of ≈75% (Figure 6g). H&E staining confirmed that CTX-(PEG_4_-MOR)_5_ caused greater tumor apoptosis compared to CTX (Figure 6h).

To investigate the underlying mechanism, the levels of EGFR, p-EGFR, AKT and p-AKT in the EGFR signaling pathway were studied. WB analysis revealed that CTX-(PEG_4_-MOR)_5_ significantly reduced EGFR, p-EGFR and p-AKT levels, while total AKT remained unchanged (Figure 6i). IF imaging further confirmed these results (Figure 6j). Additionally, body weight monitoring, organ histology, blood routine test, and blood biochemical analysis indicated that CTX-(PEG_4_-MOR)_5_ did not cause adverse effect compared to CTX, confirming its favorable biosafety profile (Figures S62 and S63). Overall, these findings suggest that CTX-(PEG_4_-MOR)_5_, a molecularly built antibody, is more effective than CTX in inhibiting tumor growth by targeting EGFR degradation.

## Conclusions

The targeted degradation strategy of membrane receptors has significantly advanced drug development and disease management. In this study, we present a novel class of molecularly built ligands (MBL-LYTACs), synthesized by conjugating membrane-receptor-targeted ligands with lysosome-localized molecules to facilitate lysosomal degradation of membrane receptors. These ligands leverage the unique physicochemical properties of lysosome-localized molecules, such as morpholine, dimethylethanamine, and low-polymerized mPEG_3/4_, to enhance receptor internalization and lysosomal accumulation, ultimately promoting efficient degradation. The methodology offers several important advantages. First, MBL-LYTACs operate independently of cell surface lysosome-shuttling receptors by modulating endocytic trafficking through lysosome-localized molecules. This avoids the limitations of lysosome-shuttling-receptor-dependent strategies, including cell-type specificity and potential toxicities from the hyperactivation of cell surface lysosome-shuttling receptors. Second, MBL-LYTACs are inherently simple, modular, and versatile. Their design can be readily adapted to target a wide range of membrane receptors by altering the targeting ligands. Finally, the strategy has the potential to expand the scope of ligand-drug conjugates, including antibody-drug conjugates, beyond conventional cytotoxic payloads, such as monomethyl auristatin E (MMAE). Importantly, many lysosome-localized groups used in our design (e.g., morpholine, low-polymerized PEGs) already exist in approved or experimental drugs,^55–57^ underscoring the clinical translatability of molecularly built ligands, especially molecularly built antibodies.

In parallel, recent elegant approaches have sought to bypass lysosomal transport receptors through alternative mechanisms. Shao et al. developed Ab-CMAs, antibody-based chimeras bearing chaperone-mediated autophagy peptides to trigger autophagy-driven degradation.^23^ Cheng et al. introduced AUTAB, which links antibodies to polyethyleneimine, enhancing internalization and induce autophagy-based receptor degradation.^58^ Liu et al. proposed TPD-NP, a nanoparticle-based system combining antibodies and PLGA nanoparticles to deliver membrane proteins into lysosomes for degradation, independent of intrinsic internalization capacity.^59^ Compared to these strategies, MBL-LYTACs displayed comparable or slightly lower degradation efficacy, but they may offer superior biosafety by avoiding the potential cytotoxicity of endosomal disruption (as in autophagy) or associated with polyethylenimine or nanoparticles. In conclusion, our study establishes MBL-LYTACs as a simple, universal, and clinically relevant strategy for the selective modulation of membrane receptor levels and the development of next-generation therapeutics.

## Supplementary Material



## Notes

Reagents and materials, experimental methods, additional experimental data, including DNA and peptide sequences, NMR and MS of azide-functionalized molecules and molecularly built ligands, and biosafety analysis of molecularly built aptamer and antibody, supplemental tables (Tables S1 and S5), and supplemental figures (Figures S1–S63) (PDF)
