# Supplementary material for: Molecularly Built Ligands Degrade Membrane Receptors via Enhancing Their Accumulation in Lysosomes
Source: ACS Cent Sci. 2025 Nov 28;11(12):2460–73. doi: 10.1021/acscentsci.5c01647 (PMC12746154; doi:10.1021/acscentsci.5c01647)
Supplement: Supplementary file 1 [file oc5c01647_si_001.pdf]

Supporting information

Molecularly Built Ligands Degrade Membrane Receptors via Enhancing Their Accumulation in Lysosomes

Dongchen Zhang[1], Xinyi Zhou[1], Jiamin Cai[1], Weihong Tan[1,2], Yanlan Liu,[1,], Zilong Zhao[1,*]*

[1]Molecular Science and Biomedicine Laboratory (MBL), State Key Laboratory of Chemo and Biosensing, College of Chemistry and Chemical Engineering, College of Biology, Aptamer Engineering Center of Hunan Province, Hunan University, Changsha, Hunan 410082, People's Republic of China

[2]The Key Laboratory of Zhejiang Province for Aptamers and Theranostics, Zhejiang Cancer Hospital, Hangzhou Institute of Medicine (HIM), Chinese Academy of Sciences, Hangzhou, Zhejiang 310022, China

Corresponding author:

*Yanlan Liu: ylliu@hnu.edu.cn,

*Zilong Zhao: zlzhao@hnu.edu.cn.

Table of Contents

1. Reagents and experimental methods 4

1.1 Reagents and cell culture 4

1.1.1 Materials 4

1.1.2 Cell lines and cell culture 5

1.2 In vitro experiments 6

1.2.1 Azide modification of molecules 6

1.2.2 Preparation of aptamer-MOR 6

1.2.3 MALDI-TOF-MS Analysis of $mPEG_{14}$ and $mPEG_{24}$ 8

1.2.4 Preparation of peptide-MOR 8

1.2.5 Preparation of folate-MOR 8

1.2.6 Preparation of nanobody-MOR and antibody-MOR 9

1.2.7 Western blot (WB) analysis 10

1.2.8 Stability analysis of MBL-LYTAC 11

1.2.9 Fluorescence imaging analysis 11

1.2.10 Flow cytometry analysis 12

1.2.11 The effects of various inhibitors on molecularly built ligands 13

1.2.12 Cell viability assay 14

1.2.13 T cell-mediated cancer cell killing 14

1.2.14 Quantitative Proteomics Analysis 15

1.2.15 RT-qPCR Analysis 15

1.3 In vivo experiments 15

1.3.1 In vivo anti-tumor study 15

1.3.2 In vivo biosafety analysis 16

1.3.3 In vivo circulation half-life 16

1.3.4 In vivo fluorescence imaging of tumor 17

1.3.5 Therapeutic efficacy of Mj5cTinv-MOR in 4T1 syngeneic breast cancer 17

1.3.6 Therapeutic efficacy of CTX-(PEG4-MOR)5 in A549 xenograft tumor 18

1.3.7 Staining analysis of tissue slides 18

1.3.8 Flow cytometric analysis of immune cells 19

1.4 Statistical analysis 20

2. Supplementary Tables 21

3. Supplementary Figures 24

4. Reference 66

1. Reagents and experimental methods

1.1 Reagents and cell culture

1.1.1 Materials

All HPLC-purified DNA sequences listed in Table S1 were purchased from Sangon Biotech Co., Ltd. (Shanghai, China). Peptides listed in Table S3 were purchased from APeptide (Shanghai, China). PD-L1-targeted nanobody (NB_{PD-L1}) listed in Table S3 was synthesized by AtaGenix (Wuhan, China). Antibodies used in the study were listed in Table S4. Epidermal growth factor (EGF, Cat. No.: AF-100-15) were obtained from PeproTech (NJ, USA). Chlorpromazine (Cat. No.: 50-53-3), Genistein (Cat. No.: 446-72-0), Amiloride (Cat. No.: 2609-46-3), Wortmannin (Cat. No.: 19545-26-7), E-64 (Cat. No.: 66701-25-5), Chloroquine (Cat. No.: 54-05-7), Bafilomycin A1 (Cat. No.: 88899-55-2), 3-Methyladenine (Cat. No.: 5142-23-4), MG132 (Cat. No.: 133407-82-6), and Cy5-azide (Cat. No.: 1621101-43-6) were purchased from MedChemExpress (NJ, USA). Lysotracker Red (C1046), Hoechst 33258 (C1018), BeyoECL Star (P0018AS) and other Western blot related regents were purchased from Beyotime (Shanghai, China). 2-Morpholinoethanamine (MOR-NH_2, Cat. No.: 2038-03-1), 3-Morpholinopropanoic acid (MOR-COOH, Cat. No.: 4497-04-5), 1-(3-Chloropropyl) piperidine hydrochloride (Cat. No.: 5472-49-1), Cy5-NHS (Cat. No.: 146368-14-1), Folate (Cat. No.: 59-30-3), DBCO-PEG_4-NHS ester (Cat. No.: 1427004-19-0), azide-mPEG_{14} (Cat. No.: 89485-61-0), azide-mPEG_{24} (Cat. No.: 89485-61-0), 2,5-dioxopyrrolidin-1-yl morpholine-4-carboxylate (Cat. No.: 107960-10-1) and 1-(2-Chloroethyl)-1H-imidazole hydrochloride (Cat. No.: 18994-78-0) were purchased from Bidepharm (Shanghai, China). 3-Morpholinopropyl bromide (Cat. No.: 125422-83-5), azide-mPEG_3 (Cat. No.: 74654-06-1), azide-mPEG_4 (Cat. No.: 606130-90-9), 3-(3-Bromopropyl) pyridine hydrobromide (Cat.

No.: 41038-63-5), Acridine Orange (Cat. No.: 494-38-2) and 2-Azido-N, N-dimethylethanamine (Cat. No.: 86147-04-8) were purchased from Leyan (Shanghai, China).

1.1.2 Cell lines and cell culture

The selection of cell lines for membrane protein degradation analysis was based on solely on the expression of the target protein. Therefore, PD-L1-expressing human non-small cell lung cancer H460 cells and mouse breast cancer 4T1 cells were used for the functional analysis of PD-L1-targeted molecularly built ligands. EGFR-expressing human non-small cell lung cancer A549 cells and cervical cancer HeLa cells were used for the functional analysis of EGFR-targeted molecularly built ligands. PTK7-expressing human colon cancer HCT116 cells and liver cancer HepG2 cells were used for the functional analysis of PTK7-targeted molecularly built ligand. Folate receptor-expressing human prostate cancer DU145 cells and cervical cancer HeLa cells were used for the functional analysis of folate receptor-targeted molecularly built ligands.

Human (H460, A549), human colon cancer HCT116 cells, human prostate cancer DU145, human embryonic kidney HEK293 cells, human umbilical vein endothelial cells (HUVECs), human acute T lymphoblastic leukemia TALL104 cells, human cervical cancer HeLa cells, human liver cancer HepG2 cells, mouse breast cancer 4T1 cells, and mouse embryonic fibroblast (MEF) cells were obtained from ATCC. A549, DU145, HeLa, HepG2, HUVECs, MEF, and TALL104 cells were cultured in Dulbecco's Modified Eagle Medium’s (DMEM, Gibco), while H460, HCT116, 4T1, and HEK293 cells were cultured in RPMI 1640 medium (Gibco) at 37 °C in a 5% CO_2 atmosphere. All cell media were supplemented with 10% fetal bovine serum (FBS, Gibco) and 100 U/mL penicillin-streptomycin (Cellgro) Trypsin (Gibco) and Mg^{2+}- and Ca^{2+}-free Dulbecco's phosphate buffered saline (DPBS, Gibco) were used for cell detachment and washing.

1.2 In vitro experiments

1.2.1 Azide modification of molecules

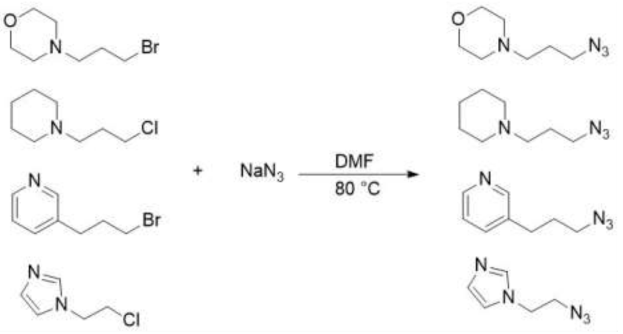

Azide functionalization was performed as previously described.[1] 208.5 mg (1.0 mmol) 3-Morpholinopropyl bromide (MOR), 198.2 mg (1.0 mmol) 1-(3-Chloropropyl) piperidine hydrochloride (PID), 281.3 mg (1.0 mmol) 3-(3-Bromopropyl)pyridine hydrobromide (PYD) or 167.3 mg (1.0 mmol) 1-(2-Chloroethyl)-1H-imidazole hydrochloride (IMZ) was mixed with 98.0 mg (1.5 mmol) sodium azide (NaN_3) in 10 mL dimethylformamide (DMF), with triethylamine (TEA) added to maintain alkalinity. The reaction solution was refluxed at 80 °C for 12 hours, followed by the addition of 2 mL ddH_2O and further refluxing for 12 hours. The reaction solution was extracted with a 1:1 mixture of ethyl acetate and ddH_2O and ddH_2O, evaporated at 45 °C, yielding azide-functionalized molecules: 219.3 mg 3-(3-azidopropyl) pyridine (Azide-PYD, yield: 90.2%), 172.0 mg 1-(2-azidoethyl) imidazole (Azide-IMZ, yield: 99.1%), 164.5 mg 4-(3-azidopropyl) morpholine (Azide-MOR, yield: 96.7%), and 189.2 mg 1-(3-azidopropyl) piperidine (Azide-PID, yield: 92.5%). The products were identified by nuclear magnetic resonance (NMR).

1.2.2 Preparation of aptamer-MOR

20 nmol azide-functionalized molecules and 2 nmol DBCO-modified DNA were mixed in 40 μL ddH_2O and 20 μL dimethyl sulfoxide (DMSO), shaken at 50 °C overnight, purified by reverse-

phase high-performance liquid chromatography (HPLC, Agilent 1260 Infinity, USA) equipped with a UV detector.

To synthesize Mj5c(Amide)-MOR, 2 nmol Mj5c-NH_2, 20 nmol MOR-COOH, 50 nmol 1-(3-dimethylaminopropyl)-3-ethylcarbodiimide (EDC) and 50 nmol N-hydroxysuccinimide (NHS) were dissolved in 40 μL ddH_2O and 40 μL DMSO, stirred for 24 hours at room temperature, and purified by reverse-phase HPLC.

The mobile phase consisted of acetonitrile and 0.1 M triethylamine acetate (TEAA, pH = 7.0) with gradient elution: 5% acetonitrile and 95% 0.1 M TEAA at 1.0 mL/min for 4 min, and then 5% to 70% ACN at 1.0 mL/min within 30 min, followed by 95% ACN at 1.0 mL/min for 15 min. Absorbance was monitored at 260 nm. Purified ligands were analyzed by electrospray ionization mass spectrometry (ESI-MS) or gel permeation chromatography (GPC). In GPC analysis, the size of the molecule was calculated by Equation S1.

$$M_w = \frac{\sum((dw/d\log M)_i \times M_{wi})}{\sum(dw/d\log M)_i} \qquad (Equation\ S1)$$

In Equation S1, w was mass, M was number-average molecular weight, $(dw/d\log M)_i$ was the relative amount of each molecular weight, M_{wi} was the corresponding molecular weight, and M_w was the final calculated weight-average molecular weight.

To assess the hydrophilicity of the molecularly built aptamers under at pH 5.5 and pH 7.0, the pH value of TEAA in the mobile phase was adjusted to 5.5 with acetic acid, and then the retention times of molecularly built aptamers at pH 5.5 and pH 7.0 were detected by RP-HPLC according to the procedure mentioned above.

1.2.3 MALDI-TOF-MS Analysis of $mPEG_{14}$ and $mPEG_{24}$

Molecular weights of $mPEG_{14}$ and $mPEG_{24}$ were determined using matrix-assisted laser desorption/ionization time-of-flight mass spectrometry (MALDI-TOF-MS, UltrafleXtreme, Germany). 1.0 μL mPEG samples and 1.0 μL dihydroxybenzoic acid (10 mg/mL) were mixed, spotted onto a metal plate, dried, and analyzed using a Smartbeam-II laser (1000 Hz, Bruker). Then the mass-to-charge ratio (m/z) values and its relative signal intensities were recorded.

1.2.4 Preparation of peptide-MOR

100 μmol GE11 or HW12, 1.0 mmol MOR-COOH, 2.5 mmol 1-(3-dimethylaminopropyl)-3-ethylcarbodiimide (EDC) and 2.5 mmol N-hydroxysuccinimide (NHS) were dissolved in 5 mL ddH_2O and 5 mL DMSO, stirred for 18 hours at room temperature, and purified by reverse-phase HPLC. The mobile phase consisted of ddH_2O and ACN with 0.1% trifluoroacetic acid with gradient elution: 21% acetonitrile and 79% ddH_2O at 1.0 mL/min for 4 min, and then 21% to 46% ACN at 1.0 mL/min within 25 min, followed by 100% ACN at 1.0 mL/min for 5 min. Absorbance at 220 nm was recorded, and collected peptide-MOR were subjected to ESI-MS analysis.

1.2.5 Preparation of folate-MOR

1 mmol folate, 0.5 mmol MOR-NH_2, 2.5 mmol EDC and 2.5 mmol NHS were dissolved in 10 mL DMSO, stirred for 24 hours, and purified by liquid chromatography-mass spectrometry (LC-MS, Shimadzu LCMS-023, Japan). The mobile phase consisted of ddH_2O and acetonitrile with 0.1% formic acid with gradient elution: 5% acetonitrile and 95% ddH_2O at 2.0 mL/min for 0.2 min, and then 5% to 95% ACN at 2.0 mL/min within 1.4 min, followed by 95% ACN at 1.0 mL/min for 0.9 min. The absorbance at 254 nm was recorded, and the collected FA-MOR was subjected to NMR analysis.

1.2.6 Preparation of nanobody-MOR and antibody-MOR

$NB_{PD\text{-}L1}$-(PEG_4-MOR)$_n$ or CTX-(PEG_4-MOR)$_n$ was prepared as previously described.[2] 5 μmol anti-EGFR antibody Cetuximab (CTX) or anti-PD-L1 nanobody ($NB_{PD\text{-}L1}$) and DBCO-PEG_4-NHS (50 μmol or 125 μmol) were mixed in 1 mL PBS at pH 7.4 and gently shaken at room temperature for 24 hours. After dialysis in a 10-cm dialysis bag (MWCO: 3000 Da) for 24 hours to remove unreacted molecules, the purified DBCO-functionalized ligands were incubated with Azide-MOR (50 μmol or 125 μmol) for two additional days before final purification via dialysis.

To determine the number of molecules (n) conjugated, Cy5-azide was used instead of Azide-MOR, and $NB_{PD\text{-}L1}$-(PEG_4-Cy5)$_n$ or CTX-(PEG_4-Cy5)$_n$ were prepared. A calibration curve of fluorescence intensity at 661 nm versus Cy5 concentration (Equation S2) was used to determine n (Equation S3).

$$Y = 441.7X + 13949 \qquad R^2 = 0.9996 \quad (Equation\ S2)$$

$$n = ((Y_n - 13949)/441.7)/C_n \qquad (Equation\ S3)$$

In Equation S2, X was the concentration of Cy5, Y was the fluorescence intensity at 661 nm of Cy5. In Equation S3, C_n was the concentration of CTX, Atz or $NB_{PD\text{-}L1}$, Y_n was the fluorescence intensity at 661 nm of CTX-(PEG_4-MOR)$_n$, Atz-(PEG_4-MOR)$_n$ and $NB_{PD\text{-}L1}$-(PEG_4-MOR)$_n$. Based on the calibration curve, $NB_{PD\text{-}L1}$-(PEG_4-MOR) could be prepared when the ratio of $NB_{PD\text{-}L1}$, DBCO-PEG_4-NHS and Azide-MOR was 1:10:10. The number on CTX-(PEG_4-MOR)$_n$ was related to the ratio between them. When the ratio between CTX, DBCO-PEG_4-NHS and Azide-MOR was 1:10:10, CTX-(PEG_4-MOR)$_5$ could be prepared. When the ratio between CTX, DBCO-PEG_4-NHS and Azide-MOR was 1:25:25, CTX-(PEG_4-MOR)$_{10}$ could be prepared. Similarly, Atz-(PEG_4-MOR)$_5$ could be prepared when the ratio between Atz, DBCO-PEG_4-NHS and Azide-MOR was 1:12:12.

Based on the calibration curve, NB_{PD-L1}-PEG_4, NB_{PD-L1}-MOR, CTX-$(PEG_4)_5$ and CTX-$(PEG_4)_5$ were prepared by changing the ratio between ligands and NHS-PEG_4 or 2,5-dioxopyrrolidin-1-yl morpholine-4-carboxylate (NHS-MOR) for further analysis according to the same procedure.

1.2.7 Western blot (WB) analysis

Cells were seeded in wells of a 6-well plate (1.0×10^5 cells/well) and incubated for 24 h. Subsequently, cells were treated with corresponding molecularly built ligands at various concentrations and times. To assess the effects of molecularly built ligands GE11-MOR and CTX-$(PEG_4$-$MOR)_5$ on EGFR signaling pathway, A549 cells were pretreated with EGF for 2 hours before ligand incubation. After treatment with various molecularly built ligands, cells were washed thrice with ice-cold DPBS, and lysed in radioimmunoprecipitation assay (RIPA) buffer (50 mM Tris-HCl, pH 7.4, 150 mM NaCl, 1% NP-40, 0.5% sodium deoxycholate, 0.1% SDS, 5 mM EDTA, 1 mM EGTA and 1% protease inhibitor cocktail) on ice for 30 minutes. Lysates were collected, centrifugated at 14,000 rpm for 30 minutes, and the protein concentration in the supernatants was determined using a NanoDrop 2000 spectrophotometer (Thermo Fisher Scientific). Protein samples (240 μg per lane) were mixed with 5× loading buffer (1:4 ratio), denatured at 100 °C for 10 minutes, and separated by 8% SDS-PAGE. Proteins were transferred onto polyvinylidene difluoride (PVDF) membranes (0.45 μm pores) using a Mini Trans-Blot system (Bio-Rad), blocked with 5% non-fat dry milk in 1× Tris-buffered saline with Tween-20 (TBST) for 1 hour, and incubated with primary antibodies overnight at 4 °C. After three washes with TBST each for 10 min, membranes were then incubated with HRP-conjugated secondary antibodies for 1 h at room temperature, washed with TBST, and visualized using chemiluminescent HRP substrate with

the ChemiDoc XRS Imaging System (Bio-Rad). Bind intensity was quantified using ImageJ software and normalized to loading controls.

1.2.8 Stability analysis of MBL-LYTAC

To compare the stability of Mj5c-MOR and Mj5cT$_{inv}$-MOR, 0.01 nmol of each ligand was added to 10 μL RPMI 1640 medium containing 10% FBS and incubated at 37 °C for different times. DNA samples (10 μL) were mixed with 2 μL 6× loading buffer, heated at 95 °C for 5 minutes, and separated on 8% polyacrylamide gel. Then the gel was stained with ethidium bromide (EB), and bands were visualized under UV light using the ChemiDoc XRS Imaging System (Bio-Rad).

1.2.9 Fluorescence imaging analysis

Lysosome staining with acridine orange was performed according to the previous report.[3] To assess the effects of nine small molecules used in this study on lysosomes, 1×10^5 H460 cells were seeded in 35-mm glass-bottom dishes and grown for 24 hours. Cells were treated with nine candidate molecules or molecularly built aptamers for 24 hours, followed by incubation with 200 μL acridine orange solution (1 mg/mL) at 37 °C for 10 minutes. Fluorescence signals were captured using a Nikon A1 Plus confocal microscope (Nikon Ti-E+A1 MP, Japan) or DxP Athena flow cytometry system (Cytek Biosciences, USA), with excitation at 488 nm and emission at 500-550nm and 650-720 nm.

To observe the internalization of molecularly built ligands, cells in a 35-mm glass-bottom dishes were treated with FAM-labeled or Cy5-labeled ligands for different times. After washing with DPBS, cells were stained with Lysotracker Red in 1640 medium at 37 °C for 15 minutes, washed, and imaged using a ZEISS LSM880 with Airyscan confocal laser scanning microscope (Carl Zeiss GmbH, Jena, Germany). FAM (FITC), Lysotracker Red and Cy5 were excited at 488

nm, 561 nm, and 640 nm, with emission collected at 500 - 550 nm, 570 - 620 nm and 650 - 720 nm, respectively.

To examine the levels of PD-L1, PTK7 and EGFR on cell surface, immunofluorescence (IF) imaging was performed. Cells in a 35-mm confocal dishes were treated with molecularly built ligands at varying concentrations for 72 h. After washing, cells were fixed with 4% paraformaldehyde for 10 minutes, blocked with 5% bovine serum albumin (BSA) for 1 hour, and incubated overnight at 4 °C with the primary antibodies against PD-L1, PTK7 or EGFR (Rabbit mAb). After washing, cells were incubated with Alexa Fluor 647-labeled Anti-Rabbit IgG (H+L) for 1.5 hours at room temperature, stained with 10 μM Hoechst solution for 10 minutes, and imaged with the Nikon A1 Plus microscope. Hoechst and Alexa Fluor 647 were excited at 405 nm and 640 nm, with emission collected at 425 - 475 nm and 650 - 720 nm, respectively.

To examine the intracellular distribution of membrane proteins, the procedure was similar to the above, except that cells were permeabilized with 0.02% Triton X-100 for 10 minutes post-fixation. Cells were incubated overnight with primary antibodies for PD-L1, EGFR or ALIX (Rabbit mAb) and LAMP1 (Mouse mAb) at 4 °C. After washing, cells were incubated with Alexa Fluor 488-labeled anti-Mouse IgG (H+L) and Alexa Fluor 594-labeled anti-Rabbit IgG (H+L)) for 1.5 hours, followed by Hoechst staining. Imaging was performed using the Nikon A1 Plus microscope. Alexa Fluor 488 and Alexa Fluor 594 were excited at 488 nm and 561nm, with emission collected at 500 - 550 nm and 570 - 620 nm, respectively.

1.2.10 Flow cytometry analysis

To analyze the binding affinity of ligands, cells were detached with DPBS containing 0.02% EDTA, washed with washing buffer (DPBS containing 4.5 g/L glucose and 5 mM $MgCl_2$), and resuspended in binding buffer (DPBS containing 4.5 g/L glucose and 5 mM $MgCl_2$). Cells (3 ×

10^5) were incubated with FAM-labeled or Cy5-labeled ligands at designated concentrations for 30 minutes at 4 °C. After washing, the cells were dispersed in 200 μL washing buffer and analyzed with the DxP Athena flow cytometry system (Cytek Biosciences, USA). Equilibrium dissociation constants (K_ds) were determined by Equation S4.

$$Y = (B_{max} \times X) / (K_d + X) \qquad (Equstion\ S4)$$

In Equation S4, X and Y were aptamer concentration and the corresponding geometric mean fluorescence intensity (GMFI) of cells in flow cytometry analysis, respectively. B_{max} represented the maximum GMFI of cells caused by the specific binding of ligands on cells. Data were processed with GraphPad Prism7.

For competition analysis, 3×10^5 H460 or A549 cells were incubated with Cy5-labeled NB_{PD-L1} or CTX (200 nM) in the presence of increasing concentrations of a-PD-L1 or anti-EGFR, respectively, followed by flow cytometric analysis.

To observe the internalization, cells were incubated with 1 μM Cy5-labeled molecularly built ligands for 1, 8 or 24 hours at 37 °C. After washing three times, cells were detached with trypsin for 5 min to eliminate interference from membrane-bound aptamers, and analyzed using the DxP Athena flow cytometry system.

1.2.11 The effects of various inhibitors on molecularly built ligands

To explore the endocytosis pathway, cells were incubated with 1 μM Cy5-labeled Mj5c-MOR (H460 cells) or 5 μM FITC-labeled GE11-MOR (A549 cells) in the presence of inhibitors (7 μM CPZ, 30 μM genistein, 5 mM AMI or 1 μM Wortmannin) for 8 or 72 hours. After 8 hours, cells were detached with trypsin, washed, and analyzed by flow cytometry. After 72 hours, proteins levels of PD-L1 or EGFR were assessed by WB.

To explore the mechanism of protein degradation, cells were incubated with 1 μM Mj5c-MOR or 5 μM GE11-MOR and degradation inhibitors (100 μM E64, 10 nM CQ, 400 nM BafA1, 5 mM 3-MA or 200 nM MG132) for 72 h at 37 °C, followed by WB analysis.

To verify the role of lysosome-localized molecules in inducing the accumulation of target proteins into lysosomes, H460 cells were co-incubated with 0.5 μM Mj5c-MOR or Mj5c-PEG_3 and different concentrations (0.25, 0.5, 1 μM) of morpholine or $mPEG_3$ for 72 h. And proteins levels of PD-L1 were assessed by WB.

1.2.12 Cell viability assay

To investigate the cytotoxicity, 1.0×10^3 cells were incubated with 1 μM molecules for 72 hours. After removing cell medium containing molecules, cells were then treated with 90 μL fresh cell medium and 10 μL MTT, and absorbance was measured at 490 nm using the BioTek Synergy Neo2 reader (BioTek). Cell viability was calculated by Equation S5.

$$Y = (A_t - A_b)/(A_0 - A_b) \times 100\% \qquad (Equation\ S5)$$

In Equation S5, A_t was the average absorbance value of each experimental group. A_0 is the average absorbance value of DPBS-treated cells. A_b was the average absorbance value of the background wells.

1.2.13 T cell-mediated cancer cell killing

H460 cells were incubated with DPBS, Mj5cT_{inv}-DBCO, Mj5cT_{inv}-MOR, Ctrl-DNA-DBCO or Ctrl-DNA1-MOR for 72 hours, followed by co-incubation with 5.0×10^5 TALL104 cells for 2 or 24 hours in fresh cell medium. After 2 hours, H460 cells were stained with PI for 10 min and analyzed via fluorescence imaging and flow cytometry. After 24 hours, IFN-γ levels in the medium were measured by ELISA.

1.2.14 Quantitative Proteomics Analysis

3×10^6 H460 cells treated with or without 1 μM Mj5c-MOR for 72 h were collected for protein extraction (n = 3). The whole cell lysate (20 μg) was separated by 10% SDS-PAGE and stained with Coomassie G-250 (Sigma-Aldrich, Cat#6104-58-1). Each lane of the SDS-PAGE gel was cut off for subsequent iTRAQ/TMT labeling and enzymatic digestion. HPLC–MS/MS identification and data analysis were performed by Novogene Co., Ltd. (Beijing, China) with Uniprot database.

1.2.15 RT-qPCR Analysis

H460 cells or A549 cells were incubated with without 1 μM molecularly built ligands for various times (1, 8, 24, 48, 72 hours). Total RNA was extracted, and cDNA synthesis was performed using cDNA Synthesis Kit (Vazyme Biotech, Cat. No.: R212-01). Gene expression levels were analyzed using SYBR qPCR Master Mix (Vazyme Biotech, Cat. No.: Q511) on an ABI7500 system (Bio-Rad, USA), and normalized to GAPDH or tubulin. Primer sequences were obtained from the Harvard University PrimerBank (Table S1).

1.3 In vivo experiments

1.3.1 In vivo anti-tumor study

Immunocompetent female BALB/c mice and immunodeficient female BALB/c nude mice (4 weeks) were purchased from SPF Biotechnol-ogy (Changsha, China) and housed in a pathogen-free facility at 25 °C with 40% humidity under a controlled light cycle. Animal care and handling procedures were performed in accordance with the guidelines of the Institutional Animal Care and Use Committee of Hunan University and the guidelines of the Regional Ethics Committee for Animal Experiments. The animal experiments involved in this work have all been approved by the Animal Care and Use Committee of Hunan University (SYXK 2023-0010).

1.3.2 In vivo biosafety analysis

Immunocompetent healthy female BALB/c mice were intravenously injected with different doses of a-PD-L1 or Mj5cT$_{inv}$-MOR (70, 105, 140 μg) through tail vein. Blood samples were collected from eyeballs at the designated times, and serum was obtained by centrifugation at 2500 g for 15 minutes. ELISA kits (Wuhan Servicebio Technology Co., Ltd., Cat. No.: GEM0018) were used to measure serum TNF-α, IL-6 and IFN-β levels. Whole blood analysis included white blood cell (WBC), red blood cell (RBC), lymphocyte (Lymph), neutrophil (Gran), monocyte (Mon), hemoglobin (HGB), hematocrit (HCT) and platelet (PLT). Alanine aminotransferase (ALT), aspartate aminotransferase (AST) and blood urea nitrogen (BUN) in the serum were analyzed as indicators of liver and renal functions.

1.3.3 In vivo circulation half-life

To analyze the circulation half-life of Mj5cT$_{inv}$-MOR, four-week-old healthy BALB/c mice were randomly divided into three groups (n = 3) and injected with 5 nmol Cy5-labeled Mj5c-MOR, Mj5cT$_{inv}$-DBCO, or Mj5cT$_{inv}$-MOR via the tail vein, respectively. To analyze the circulation half-life of CTX-(PEG$_4$-MOR)$_5$, four-week-old healthy BALB/c nude mice were randomly divided into two groups (n = 3) and injected with 80 μg Cy5-labeled CTX or CTX-(PEG$_4$-MOR)$_5$ via the tail vein, respectively. At designated time points post injection, 10 μL of blood was taken from the mouse tail vein at after injection, and diluted in a sampling tube containing 190 μL of saturated EDTA. The fluorescence of Cy5 in the blood was detected by TECAN-spark multifunctional microplate reader (Thermo Fisher Scientific).

To assess the in vivo stability of CTX-(PEG$_4$-MOR)$_5$, CTX-(PEG$_4$-Cy5)$_5$, in which Cy5 substituted MOR, was prepared. 80 μg of CTX-(PEG$_4$-Cy5)$_5$ was administered to healthy BALB/c nude mice via tail vein injection. At designated time points, 10 μL of blood was collected by tail

puncture and anticoagulated with 190 μL of EDTA solution. The total fluorescence intensity of the solution (F_0) was measured. Then, these samples were ultrafiltered using a 3 kDa cutoff filter at 14,000 g for 10 minutes, and the fluorescence intensity of the filtrate (F_t) was recorded. The stability of CTX-(PEG$_4$-Cy5)$_5$ was evaluated by calculating the proportion of Cy5 retained on the antibody. The retention rate of Cy5 on the antibody was then calculated as $(1 - F_t / F_o) \times 100\%$.

1.3.4 In vivo fluorescence imaging of tumor

The 4T1 syngeneic breast tumor model was established by subcutaneous injecting 5×10^6 4T1 cells into the right flank of BALB/c mice. After 14 days, tumor-bearing mice were divided into three groups (n = 3, tumor size ≈200 mm^3) and treated with 5 nmol Cy5-labeled Ctrl-DNA1-MOR, Mj5cT$_{inv}$-DBCO or Mj5cT$_{inv}$-MOR, respectively. A549 xenograft model was established by subcutaneous injecting 5×10^6 A549 cells in the right flank of BALB/c nude mice. After 28 days, tumor-bearing mice were divided into three groups (n = 3, tumor size ≈200 mm^3) and treated with 80 μg Cy5-labeled IgG-(PEG$_4$-MOR)$_5$, CTX, or CTX-(PEG$_4$-MOR)$_5$ via tail vein, respectively. At the designated time points, whole-body in vivo fluorescence imaging of these mice was recorded with IVIS® Lumina II in vivo imaging system (Caliper LifeSicence, USA). After 48 h, the mice were sacrificed, tumors and organs were collected and analyzed by fluorescence imaging.

1.3.5 Therapeutic efficacy of Mj5cTinv-MOR in 4T1 syngeneic breast cancer

The 4T1 syngeneic breast tumor model was established by subcutaneous injecting 5×10^6 4T1 cells into the right flank of BALB/c mice. After 10 days, tumor-bearing mice were divided into eight groups (n = 5, tumor size ≈100 mm^3) and treated with DPBS, Mj5cT$_{inv}$-DBCO, IgG, a-PD-1 (anti-PD-1 antibody), a-PD-L1 (anti-PD-L1 antibody), Mj5cT$_{inv}$-MOR, Mj5cT$_{inv}$-MOR plus IgG, and Mj5cT$_{inv}$-MOR plus a-PD-1, respectively. Single-agent treatment (105 μg) was intravenously administered every two days for 7 times. In combination treatments, each group of Mj5cT$_{inv}$-MOR

(105 μg per animal) and antibody or IgG (105 μg per animal) was intravenously injected on alternating days for 7 cycles. The body weight and the tumor volume of the mice were recorded every two days. The tumor volume was measured every two days using the formula: Volume = (length × width^2)/2. After 21 days, the mice were euthanatized, and tumors and organs were harvested for further analysis. The tumor growth inhibition (TGI) rate was calculated as: TGI = $(1-V_t/V_0) \times 100\%$, where V_0 was the average tumor volume in DPBS-treated mice, and V_t was the average tumor volume in experimental groups. Survival rates were recorded over 45 days, and blood samples were also collected for hematological and biochemical analysis.

1.3.6 Therapeutic efficacy of CTX-(PEG4-MOR)5 in A549 xenograft tumor

A549 xenograft model was established by subcutaneous injecting 5×10^6 A549 cells in the right flank of BALB/c nude mice. After 20 days, tumor-bearing mice were divided into four groups (n = 5, tumor size ≈100 mm^3) and treated with DPBS, 80 μg IgG-$(PEG_4\text{-}MOR)_5$, 80 μg CTX, or 80 μg CTX-$(PEG_4\text{-}MOR)_5$ via tail vein every three days for 5 times. The body weight and the tumor volume of the mice were recorded every two days. At 23 days, the mice were euthanatized, and tumors and organs were harvested for further analysis. The tumor size and the tumor growth inhibition rate were calculated using the same formula as above. Blood samples were collected for routine test and biochemical analysis.

1.3.7 Staining analysis of tissue slides

Tissue processing and staining were performed as previously described.[4] Tumors and organs were fixed in 4% paraformaldehyde overnight, dehydrated in graded alcohol (75%-100%), benzene and xylene, and embedded in paraffin. Samples were sectioned into 4-μm thick slices. Before staining, sections were subjected to deparaffinization and rehydration.

For hematoxylin and eosin (H&E) staining, the rehydrated slices were sequentially treated with hematoxylin solution, hematoxylin differentiation solution and hematoxylin bluing solution (Wuhan Servicebio Technology Co., Ltd., Cat. No.: G1004, 1039, 1040), followed by ethanol immersion and eosin staining (Wuhan Servicebio Technology Co., Ltd., Cat. No.: G1001). After dehydration and sealing, slides were analyzed using Pannoramic MIDI digital slice scanning system (3DHISTECH, Hungary).

For immunohistochemistry (IHC) staining, the rehydrated sections subject to antigen retrieval, followed by treatment with 3% BSA for 30 minutes. Then, the sections were incubated with primary antibody against PD-L1 at 4 °C overnight. After incubation with HRP-labeled secondary antibody for 1 h, sections were stained with diaminobenzidine (DAB), counterstaining with hematoxylin, dehydrated, and analyzed using the Pannoramic MIDI digital slice scanning system (3DHISTECH, Hungary). PD-L1-positive signals appeared brown-yellow, while nuclei stained blue.

For immunofluorescence imaging analysis, after blockade with 3% BSA for 30 minutes, the sections were incubated with primary antibody of EGFR, p-EGFR, AKT and p-AKT (Rabbit mAb) at 4 °C for 24 h, followed by incubation with secondary antibody Alexa Fluor 647-goat anti-rabbit IgG (H&L) at room temperature for 2 h. Next, the sections were stained with 10 μg/mL DAPI solution to stain cell nucleus for 10 min. Finally, sections were sealed with coverslips using ProLong™ Glass Antifade Mountant (Thermo Fisher Scientific) and analyzed by fluorescence imaging.

1.3.8 Flow cytometric analysis of immune cells

The flow cytometric analysis of immune cells was performed according to the previous report.[5,6] After mice were euthanized, tumors and the draining lymph nodes were excised from

mice for preparing single cell suspensions. Briefly, tumors and lymph nodes were cut into small pieces and then incubated with digestion solution (RPMI-1640 medium containing 1 mg/mL collagenase IV, 1 mg/mL collagenase I, 1 mg/mL hyaluronidase and 0.2 mg/mL DNase I). After that, the samples were centrifuged, and the cell pellets were resuspended. Then, cells from the draining lymph nodes were blocked by 0.5% BSA in DPBS for 1 h, then stained with PE-labeled anti-CD11c antibody (excited by 488 nm laser) and PE-Cy7-labeled anti-CD86/CD80 antibody (excited by 561 nm laser) at 4 °C for 30 minutes. To investigate the tumor-infiltrating $CD8^+$ T cells, cells from tumors were blocked by 0.5% BSA (in DPBS) and stained with APC-labeled anti-CD3 antibody (excited by 640 nm laser) and PE-labeled anti-CD8 antibody at 4 °C for 30 minutes. Finally, cells were washed with DPBS twice and analyzed by flow cytometry. The gating strategy for mature dendritic cells was established based on the proportions of $CD80^+$ and $CD86^+$ cells (32.47%) in untreated groups.[6] $CD8^+$ cell populations were gated according to their proportions observed in untreated (26%) and antibody-treated (37%) tumors in previous study.[5]

1.4 Statistical analysis

All statistical data were presented as mean ± SD of multiple independent experiments or replicate samples. Data were proceeded with GraphPad Prism software. The statistical significances were evaluated using one-way ANOVA with a Tukey's post hoc test, and a P value of < 0.05 was considered statistically significant for all results (ns: not significant, *p < 0.05, ***p < 0.01, ***p < 0.001, ****p < 0.0001).

2. Supplementary Tables

Table S1. DNA sequences used in this study.

Name	Sequence (5' → 3')	Purpose of DNA in the study
Mj5c-DBCO	DBCO-TAC AGG TTC TGG GGG GTG GGT GGG GAA CCT GTT	
Mj5cT_{inv}-DBCO	DBCO-TAC AGG TTC TGG GGG GTG GGT GGG GAA CCT GTT $T_{inv}T_{inv}$	For analysis or preparing molecularly built ligand
Mj5c-NH_2	NH_2-C7-TAC AGG TTC TGG GGG GTG GGT GGG GAA CCT GTT	
Ctrl-DNA1-DBCO	DBCO-ATT ACC TCT AAA TCA CTG CTC TGT AAC ATG GTC GCG CTA GG	For analysis or preparing control ligand
Sgc8-DBCO	DBCO-ATC TAA CTG CTG CGC CGC CGG GAA AAT ACT GTA CGG TAG A	For analysis or preparing molecularly built ligand
Ctrl-DNA2-DBCO	DBCO-ATC TAA CTG ATT ATT ATT ATT ATT ATT ATT ATT CGG TTA GA	For analysis or preparing control ligand
PD-L1 forward primer	GGGCGTTTACTATCACGGCT	
PD-L1 reverse primer	AGGGCAGCATTTCCCTTCAA	
GAPDH forward primer	CTACCCCCAATGTGTCCGTC	
GAPDH reverse primer	TGAAGTCGCAGGAGACAACC	For PCR analysis
EGFR forward	CGCAGGTCTCAAACTGAAGC	
EGFR reverse primer	AACGAGGAAACTAACCGCCG	
Tubulin forward primer	ATGAGAGAGTGCATATCGAT	
Tubulin reverse primer	TTCACTGAAGAAGGTGTTGA	

Table S2. Mass identification of molecularly built aptamers.

Molecularly built aptamers	Theoretical mass (Da)	Actual mass (Da)	Mass error (Da)	Error (%)
Mj5c-PYD	11009.4	11009.7	+ 0.3	0.0027
Mj5c-IMZ	10984.5	10984.6	+ 0.1	0.0009
Mj5c-MOR	11018.2	11018.2	0	0
Mj5c-DEA	10961.4	10962.0	+ 0.6	0.0055
Mj5c-PID	11014.2	11016.2	+ 2	0.0182
Mj5c-PEG_3	11036.5	11037.0	+ 0.5	0.0045
Mj5c-PEG_4	11080.5	11081.3	+ 0.8	0.0072
Mj5cT_{inv}-MOR	11625.6	11624.5	- 1.1	0.0095
Mj5c(Amide)-MOR	10684.2	10682.5	- 1.7	0.0159
Ctrl-DNA1-MOR	13199.7	13198.7	- 1	0.0076
Sgc8-MOR	13605.3	13605.5	+ 0.2	0.0015
Ctrl-DNA2-MOR	13246.8	13246.1	- 0.7	0.0053
Mj5c-PEG_{14}	16602.0	16601.0	- 1	0.0060
Mj5c-PEG_{24}	18000.0	18005.0	+ 5	0.0278

Table S3. Peptides and Nanobody sequences used in this study.

Name	Amino acid sequence (N-terminus to C-terminus)
GE11-MOR	MOR-YHWYGYTPQNVI
HW12-MOR	MOR-HYPYAHPTHPSW
NB_{PD-L1}	MKHLWFFLLLVAAPRWVLSQVQLQESGGGLVQPGGSLRLSCAASGK MSSRRCMAWFRQAPGKERERVAKLLTTSGSTYLADSVKGRFTISQNN AKSTVYLQMNSLKPEDTAMYYCAADSFEDPTCTLVTSSGAFQYWGQ GTQVTVSSGSHHHHHH

Table S4. Antibodies used in this study.

Antibody name	Source (identifier)	Purposes
Anti-PD-L1 rabbit antibody	Cell Signaling Technology (13684)	For WB, IF and IHC analysis
Anti-EGFR rabbit antibody	Cell Signaling Technology (4267)	For WB and IF analysis
Anti-PTK7 rabbit antibody	Cell Signaling Technology (25618)	
Anti-phospho-EGFR rabbit antibody	Aifang Biological (AF300274)	
Anti-AKT rabbit antibody	Aifang Biological (AF300966)	
Anti-phospho-AKT rabbit antibody	Aifang Biological (AF300996)	
Anti-LAMP1 mouse antibody	Cell Signaling Technology (15665)	For IF analysis
Anti-ALIX rabbit antibody	Proteintech (67715-1)	
Anti-FRα rabbit antibody	Abcam (ab221543)	For WB analysis
Anti-Tubulin mouse antibody	Absin (abs137976)	
Anti-GAPDH mouse antibody	Absin (abs830030)	
Anti-Actin rabbit antibody	Cell Signaling Technology (4970)	
Atezolizumab (Atz)	Aladdin (A411996)	For in vivo therapeutic analysis
Normal mouse immunoglobulin G (IgG)	Beyotime (A7028)	
Anti-mouse PD-1 antibody	BioXcell (BE0273)	
Anti-mouse PD-L1 antibody	BioXcell (BP0101)	
Cetuximab (CTX)	Aladdin (C302740)	
Alexa Fluor® 488-labeled donkey anti-mouse IgG (H+L)	Abcam (Ab150105)	Secondary antibodies for IF analysis
Alexa Fluor® 594-labeled donkey anti-rabbit IgG (H+L)	Jackson (711-585-152)	
Alexa Fluor® 647-labeled goat anti-rabbit IgG (H+L)	Beyotime (A0468)	
PE-labeled anti-CD11c mouse antibody	Abcam (Ab316176)	For flow cytometric analysis
PE-Cy7-labeled anti-CD86 rabbit antibody	Absin (Abs1850145)	
APC-labeled anti-CD4 rabbit antibody	Abcam (Ab252152)	
PE-labeled anti-CD8 mouse antibody	Absin (Abs182402)	

3. Supplementary Figures

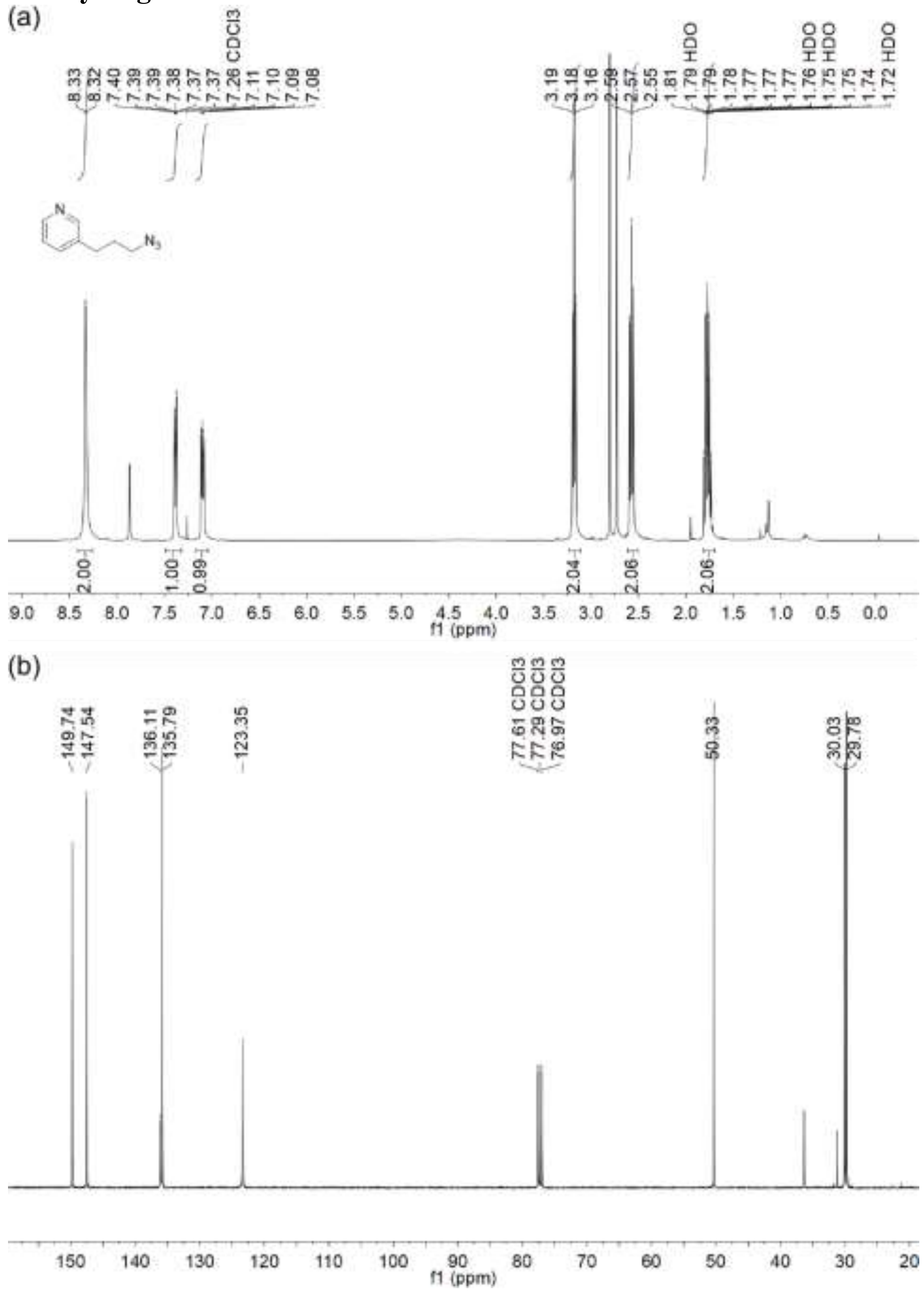

Figure S1. NMR spectrum of Azide-PYD. (**a**) ^{1}H NMR (400 MHz, Chloroform-d) δ 8.33 (d, J = 4.5 Hz, 2H), 7.38 (dt, J = 7.8, 2.0 Hz, 1H), 7.10 (dd, J = 7.9, 4.8 Hz, 1H), 3.18 (t, J = 6.7 Hz, 2H), 2.61 - 2.51 (m, 2H), 1.82 - 1.69 (m, 2H). (**b**) ^{13}C NMR (101 MHz, Chloroform-d) δ 149.94, 147.74, 136.31, 135.99, 123.55, 50.53, 30.23, 29.98.

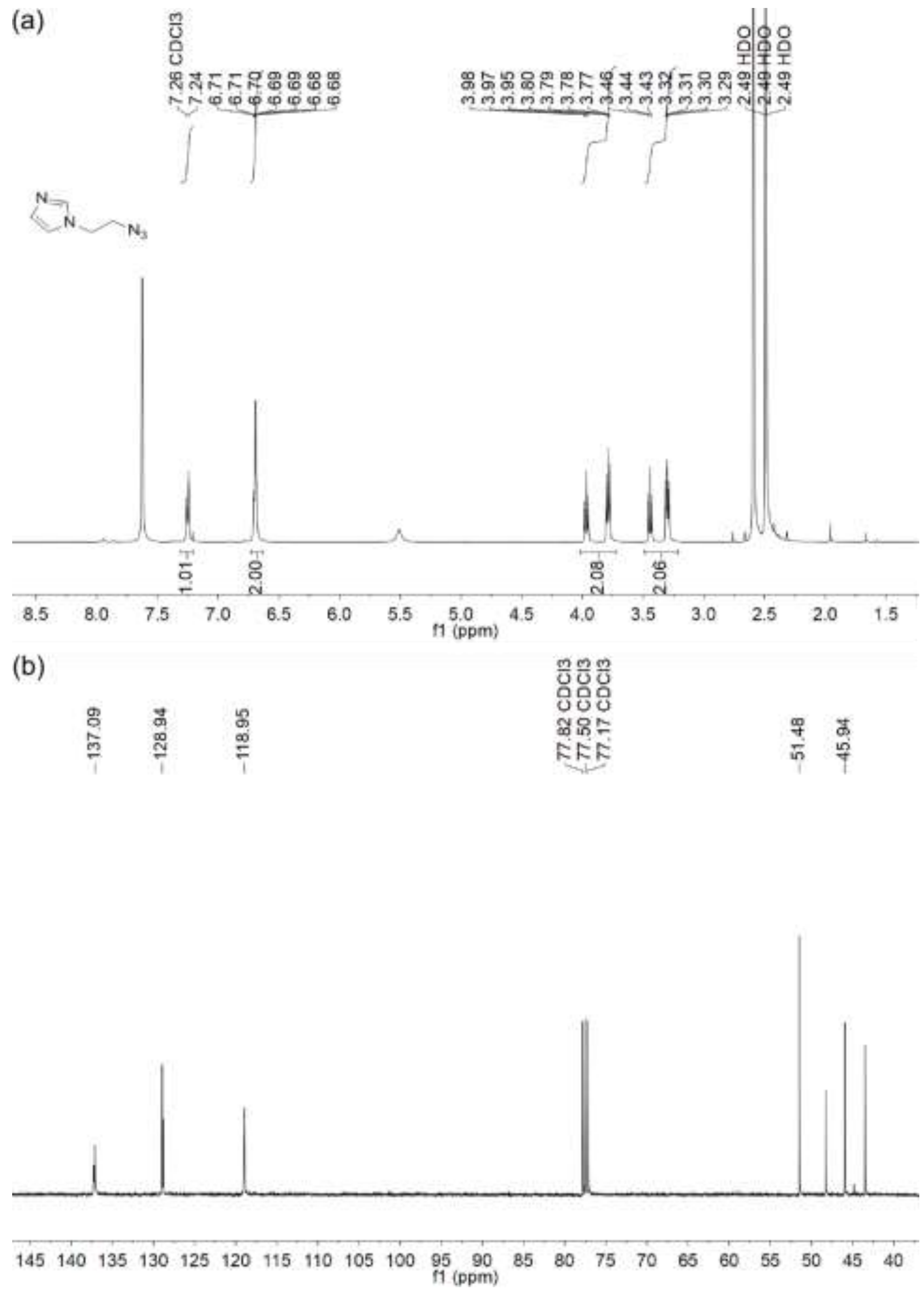

Figure S2. NMR spectrum of Azide-IMZ. (**a**) ^{1}H NMR (400 MHz, Chloroform-d) δ 7.24 (s, 1H), 6.69 (q, J = 3.6, 2.8 Hz, 2H), 3.88 (dt, J = 71.3, 5.8 Hz, 2H), 3.37 (dt, J = 56.1, 5.6 Hz, 2H). (**b**) ^{13}C NMR (101 MHz, Chloroform-d) δ 137.09, 128.94, 118.95, 51.48, 45.94.

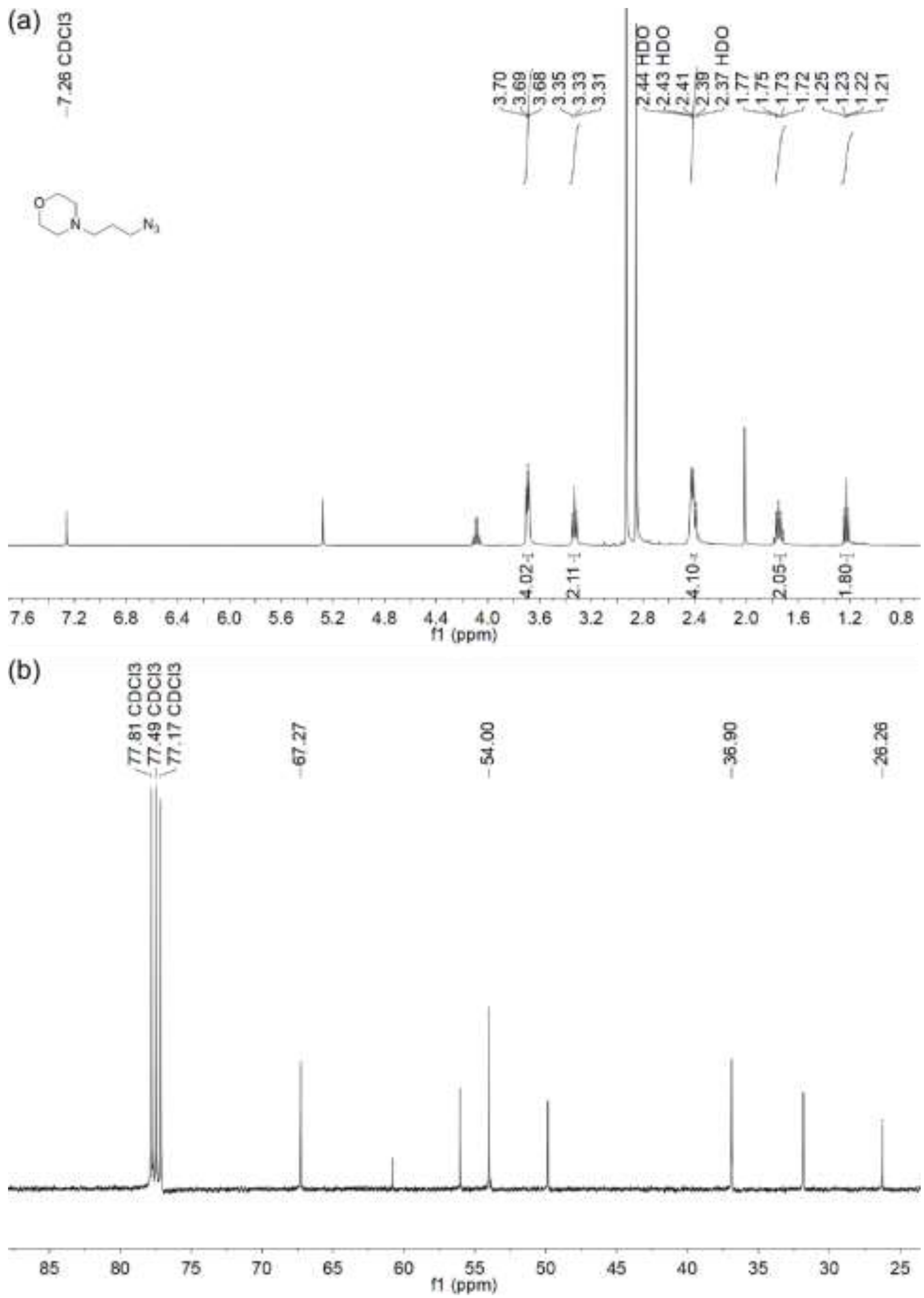

Figure S3. NMR spectrum of Azide-MOR. (**a**) ^{1}H NMR (400 MHz, Chloroform-d) δ 3.69 (t, J = 4.7 Hz, 4H), 3.33 (t, J = 6.7 Hz, 2H), 2.40 (d, J = 7.5 Hz, 4H), 1.74 (q, J = 6.9 Hz, 2H), 1.23 (t, J = 7.2 Hz, 2H). (**b**) ^{13}C NMR (101 MHz, Chloroform-d) δ 67.27, 54.00, 36.90, 26.26.

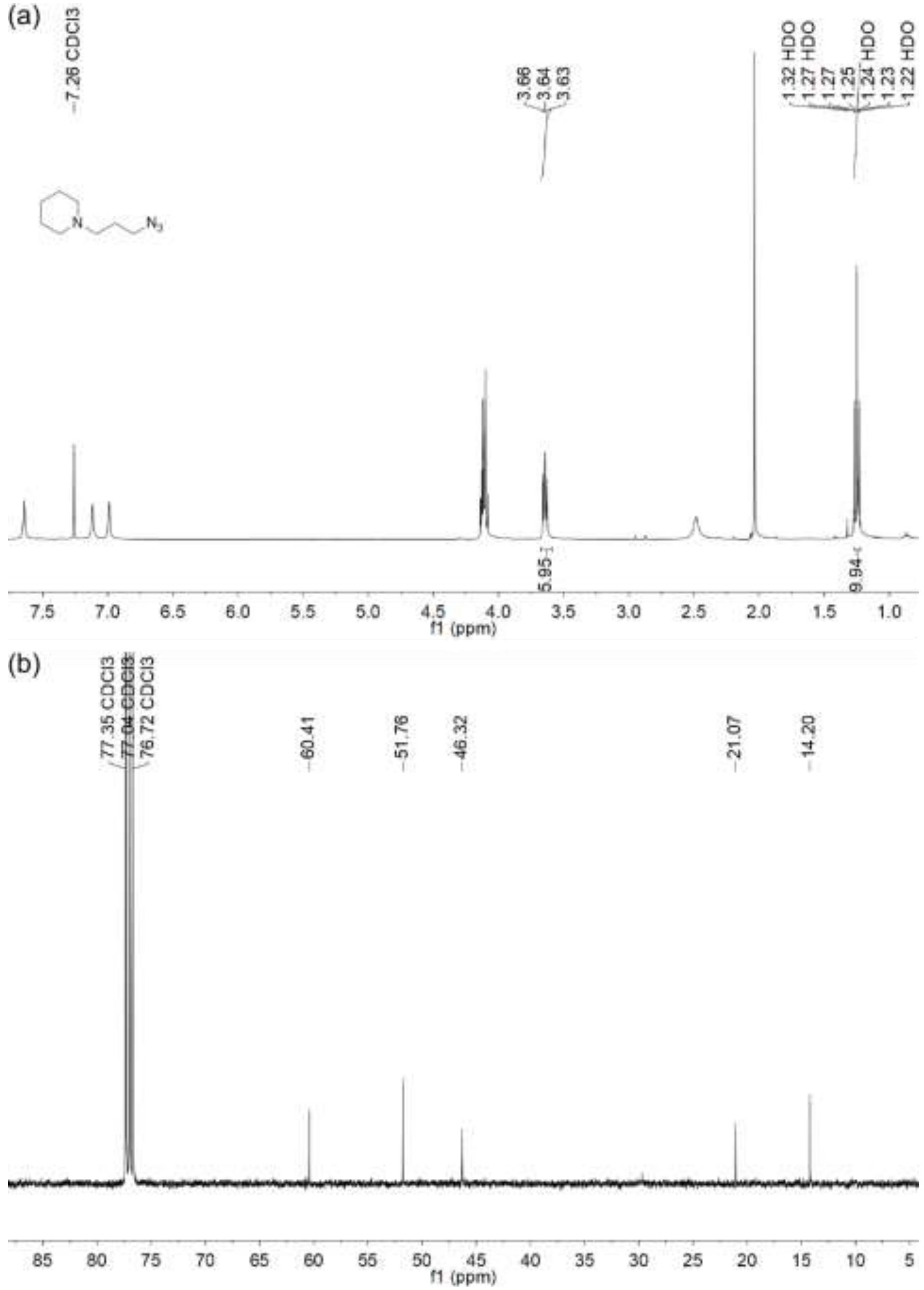

Figure S4. NMR spectrum of Azide-PID. (**a**) ^{1}H NMR (400 MHz, Chloroform-d) δ 3.64 (t, J = 5.7 Hz, 6H), 1.25 (t, J = 7.1 Hz, 10H). (**b**) ^{13}C NMR (101 MHz, Chloroform-d) δ 60.41, 51.76, 46.32, 21.07, 14.20.

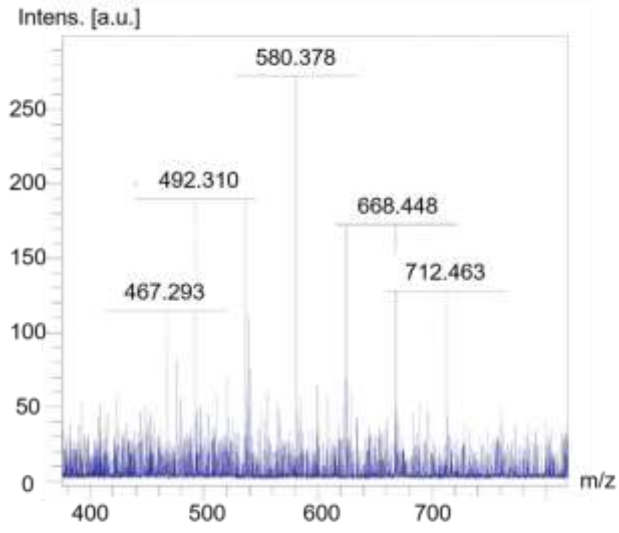

Figure S5. MALDI-TOF-MS analysis of molecular weight of Azide-PEG_{14}.

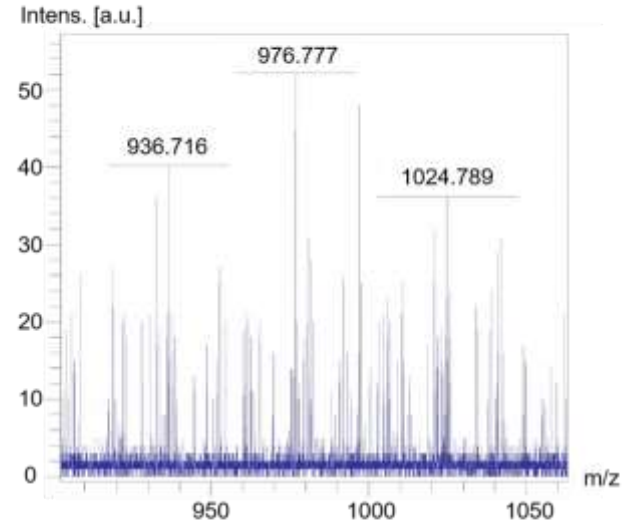

Figure S6. MALDI-TOF-MS analysis of molecular weight of Azide-PEG_{24}.

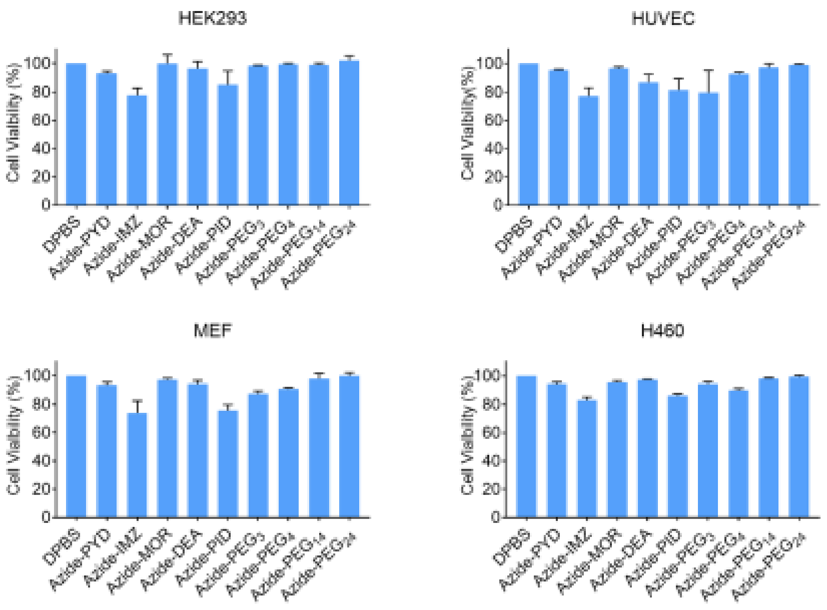

Figure S7. Cell viability assay of cells treated with 1 μM molecules for 72 h. All data were presented as mean ± SD, n = 3.

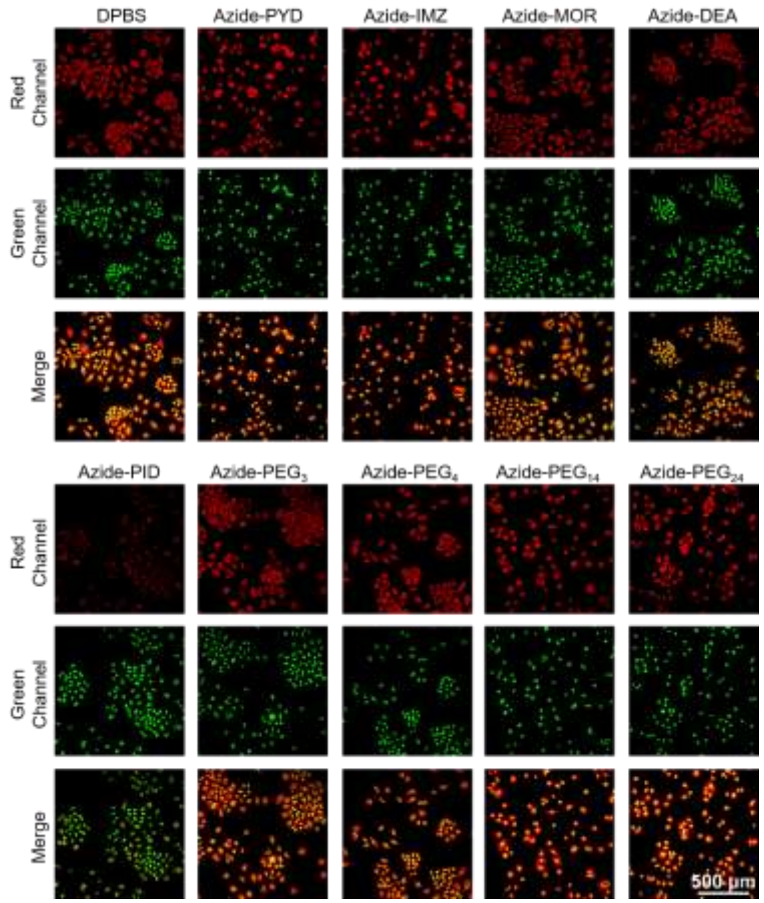

Figure S8. Fluorescence imaging of H460 cells stained with acridine orange after treatment with different azide-functionalized compounds. The concentration of the azide-functionalized compounds was 1 μM and the incubation time was 24 h. Scale bar = 500 μm.

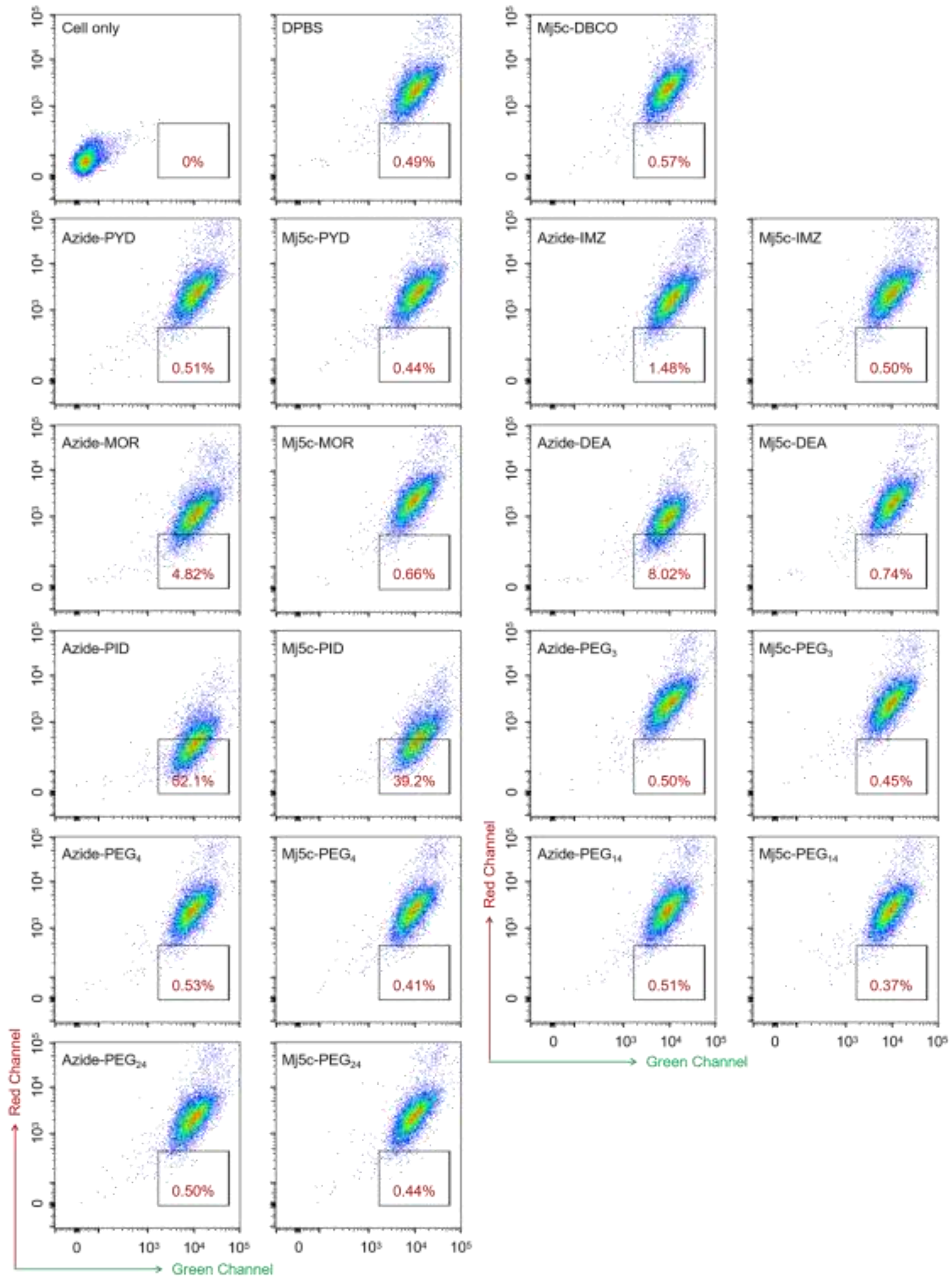

Figure S9. Flow cytometry analysis of H460 cells stained with acridine orange after treatment with different compounds. The concentration of all compounds was 1 μM and the incubation time was 24 h.

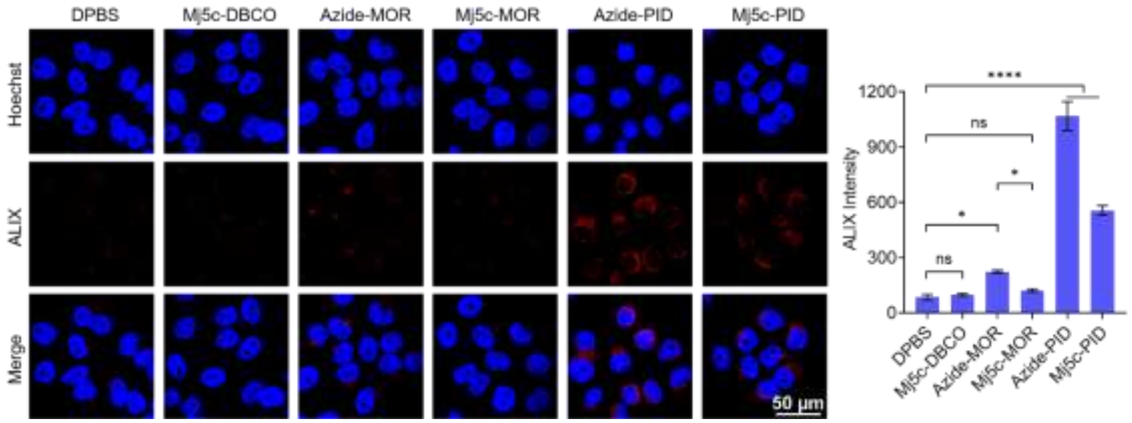

Figure S10. Visualization and quantification of ALIX in H460 cells treated with 1 μM Mj5c-DBCO, Azide-MOR, Mj5c-MOR, Azide-PID or Mj5c-PID for 24 h. Scale bar = 50 μm. All data were presented as mean ± SD of three separate images from confocal microscopy. Statistical significance was determined by one-way ANOVA with a Tukey post hoc test. ns: no significance, *P < 0.05, ****P < 0.0001.

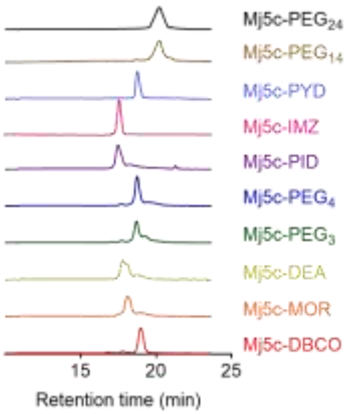

Figure S11. HPLC purification profiles of molecularly built aptamers.

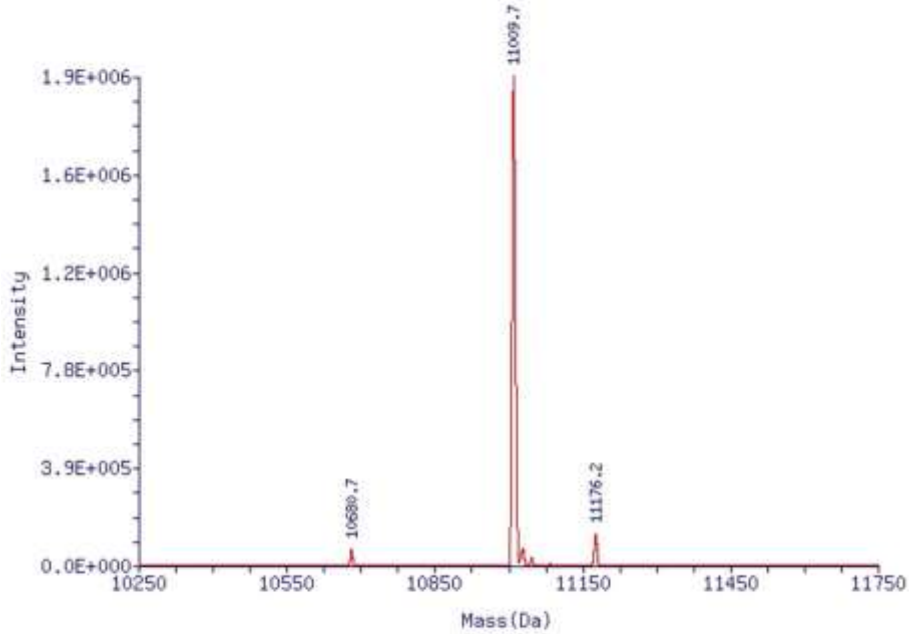

Figure S12. ESI-MS analysis of molecular weight of Mj5c-PYD. Calculated: 11009.4 Da. Found: 11009.7 Da.

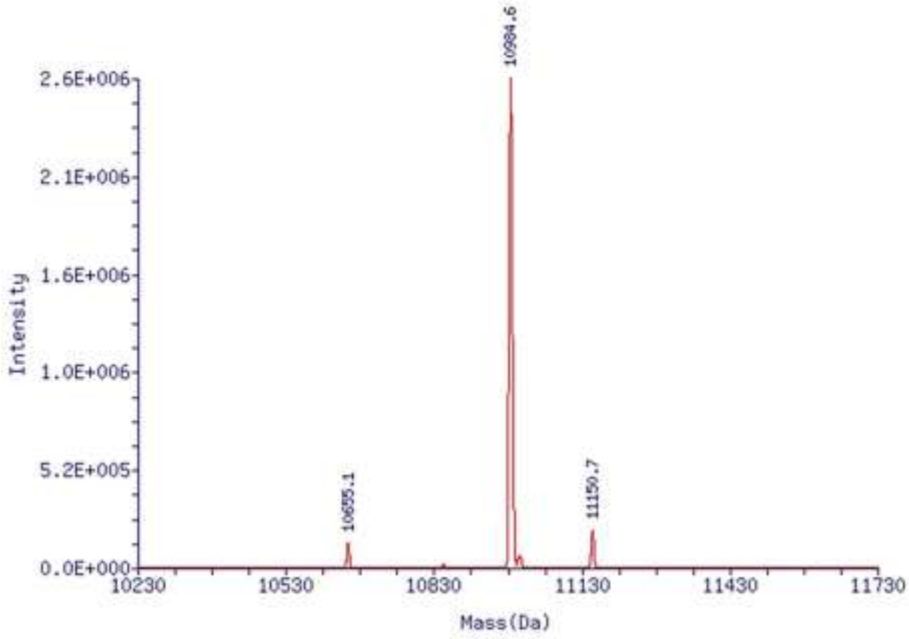

Figure S13. ESI-MS analysis of molecular weight of Mj5c-IMZ. Calculated: 10984.5 Da. Found: 10984.6 Da.

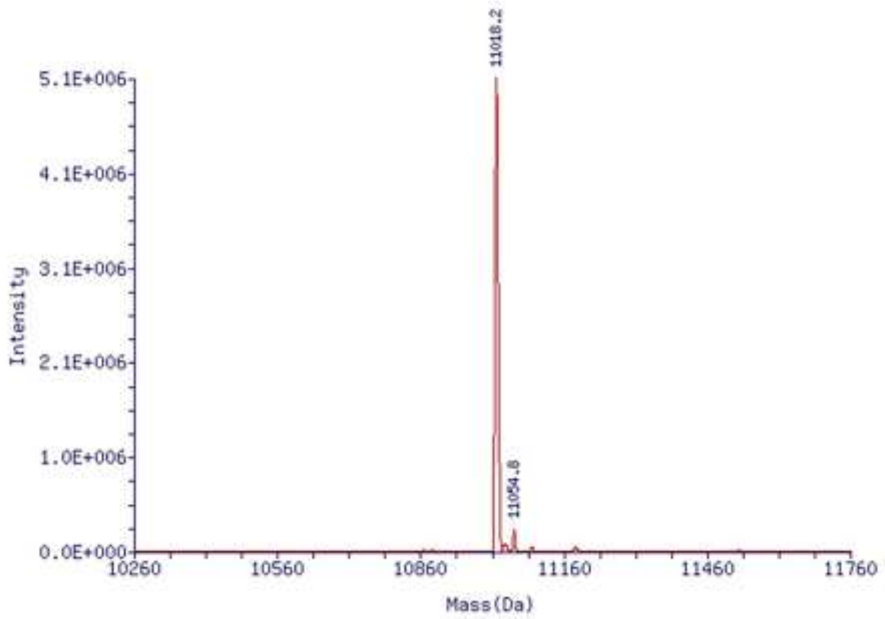

Figure S14. ESI-MS analysis of molecular weight of Mj5c-MOR. Calculated: 11018.2 Da. Found: 11018.2 Da.

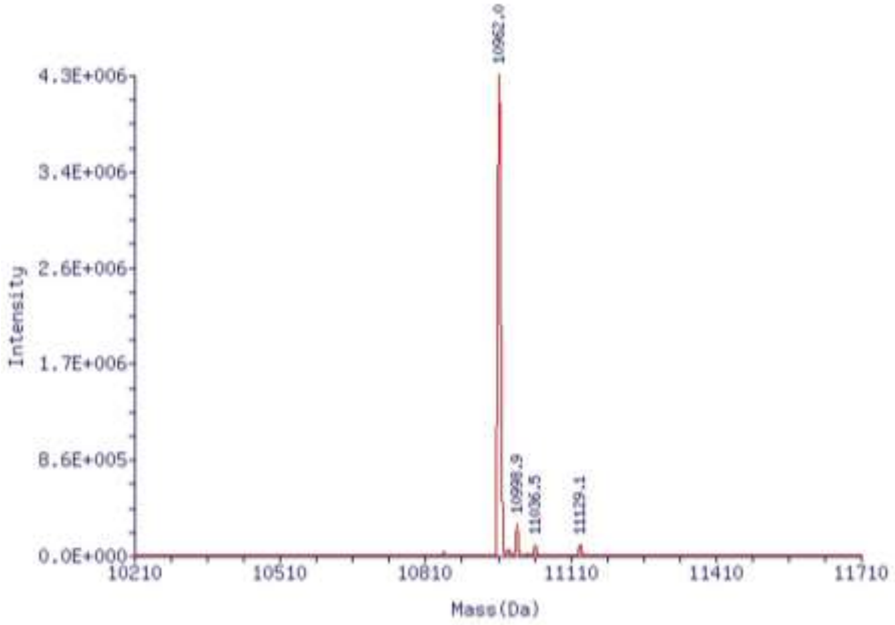

Figure S15. ESI-MS analysis of molecular weight of Mj5c-DEA. Calculated: 10961.4 Da. Found: 10962.0 Da.

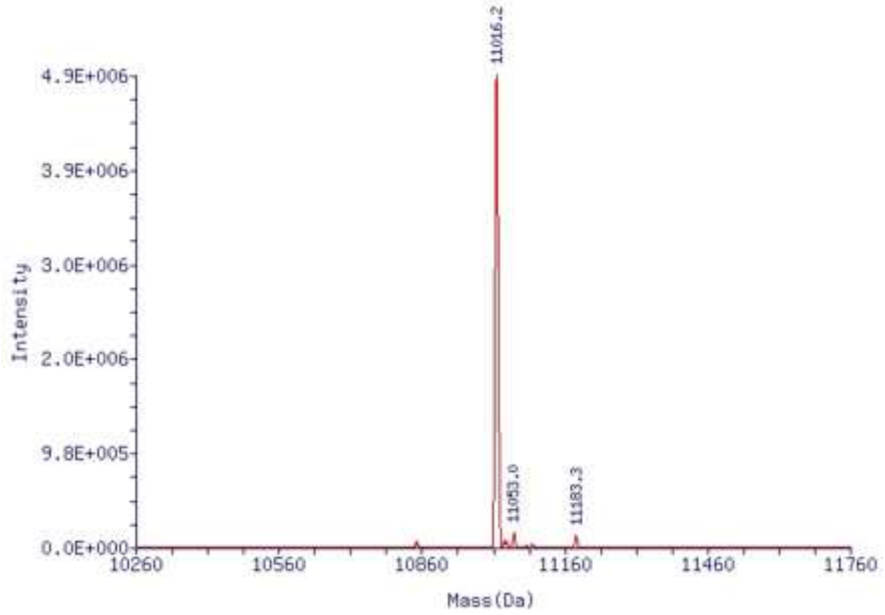

Figure S16. ESI-MS analysis of molecular weight of Mj5c-PID. Calculated: 11014.2 Da. Found: 11016.2 Da.

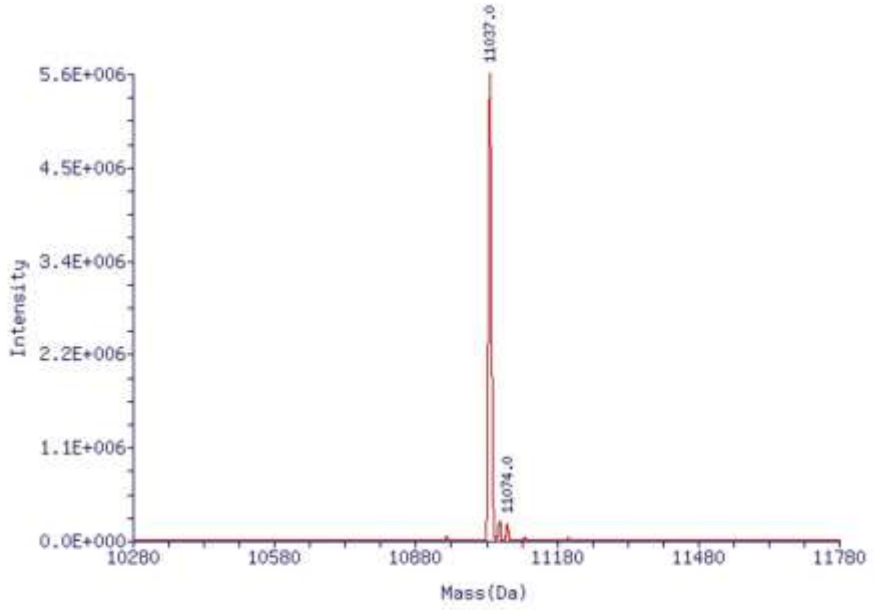

Figure S17. ESI-MS analysis of molecular weight of Mj5c-PEG_3. Calculated: 11036.5 Da. Found: 11037.0 Da.

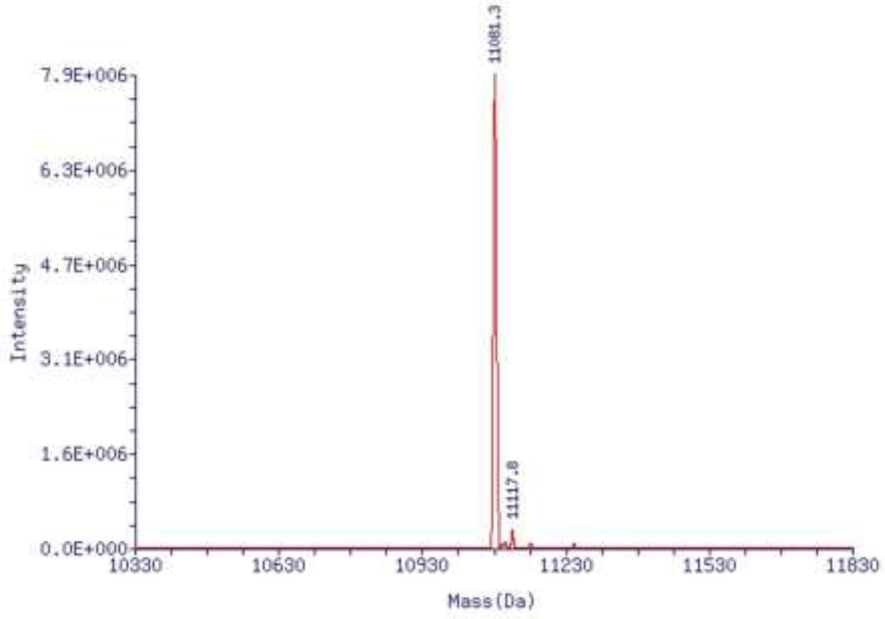

Figure S18. ESI-MS analysis of molecular weight of Mj5c-PEG$_4$. Calculated: 11080.5 Da. Found: 11081.3 Da.

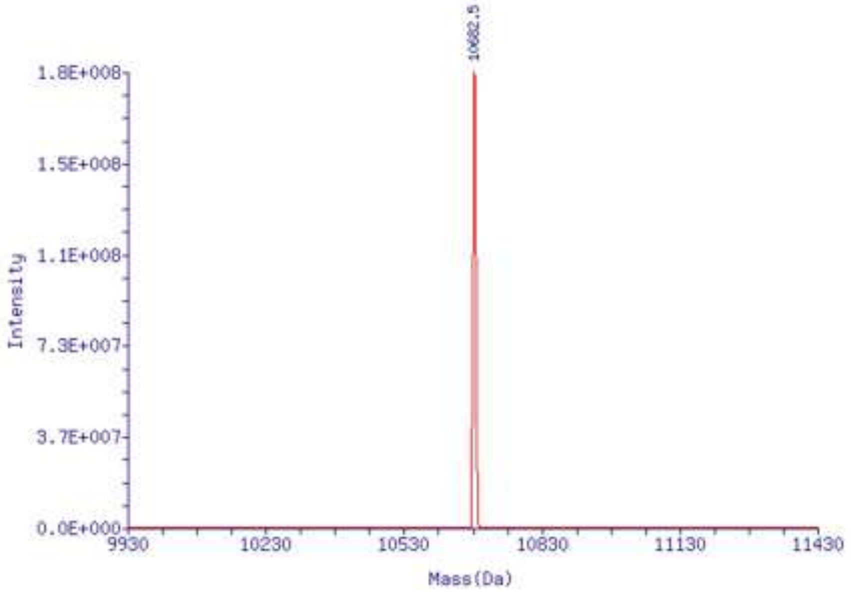

Figure S19. ESI-MS analysis of molecular weight of Mj5c(Amide)-MOR. Calculated: 10684.2 Da. Found: 10682.5 Da.

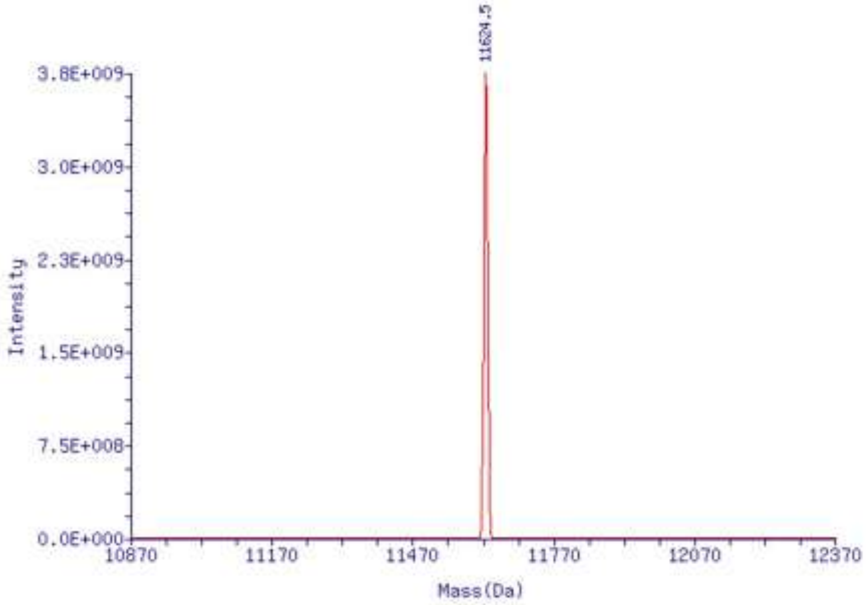

Figure S20. ESI-MS analysis of molecular weight of Mj5cT$_{inv}$-MOR. Calculated: 11625.6 Da. Found: 11624.5 Da.

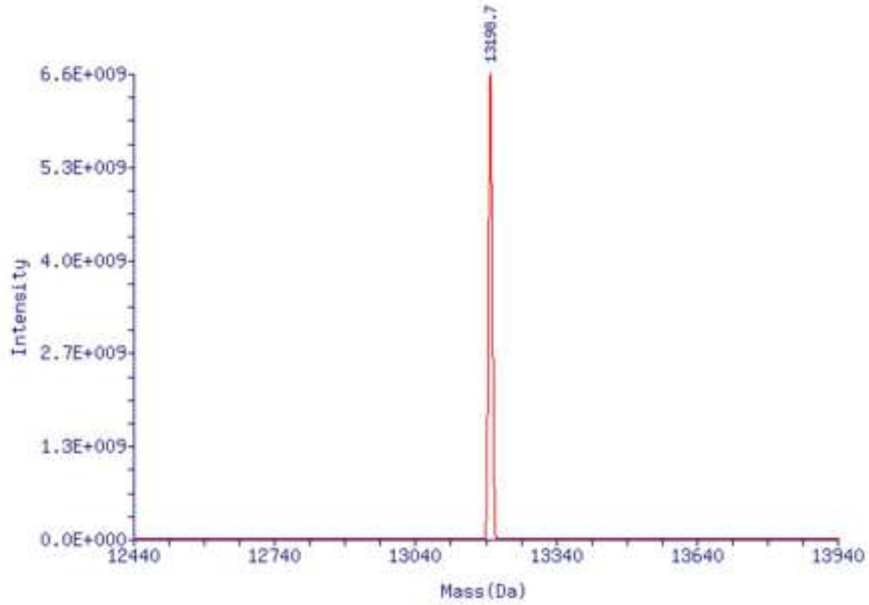

Figure S21. ESI-MS analysis of molecular weight of Ctrl-DNA1-MOR. Calculated: 13199.7 Da. Found: 13198.7 Da.

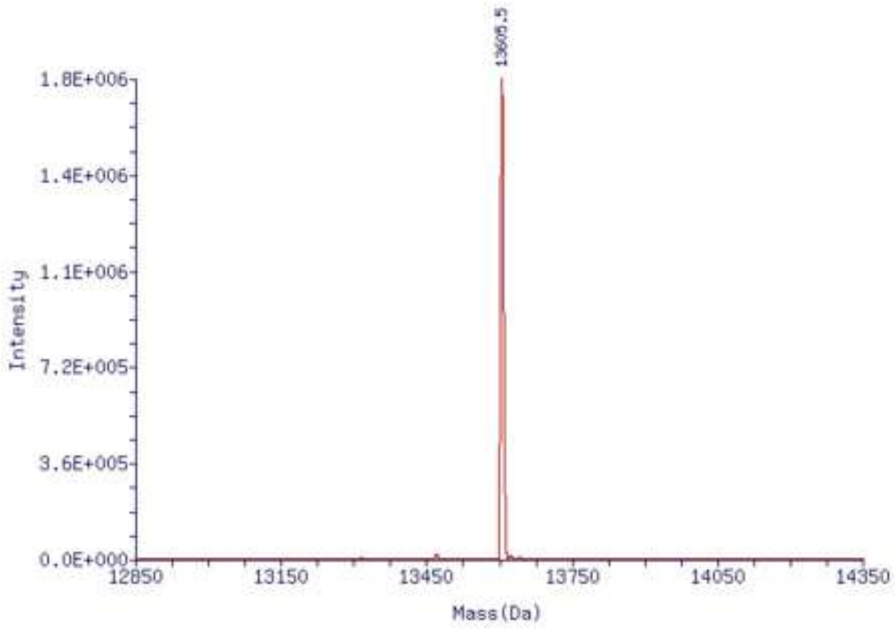

Figure S22. ESI-MS analysis of molecular weight of Sgc8-MOR. Calculated: 13605.3 Da. Found: 13605.5 Da.

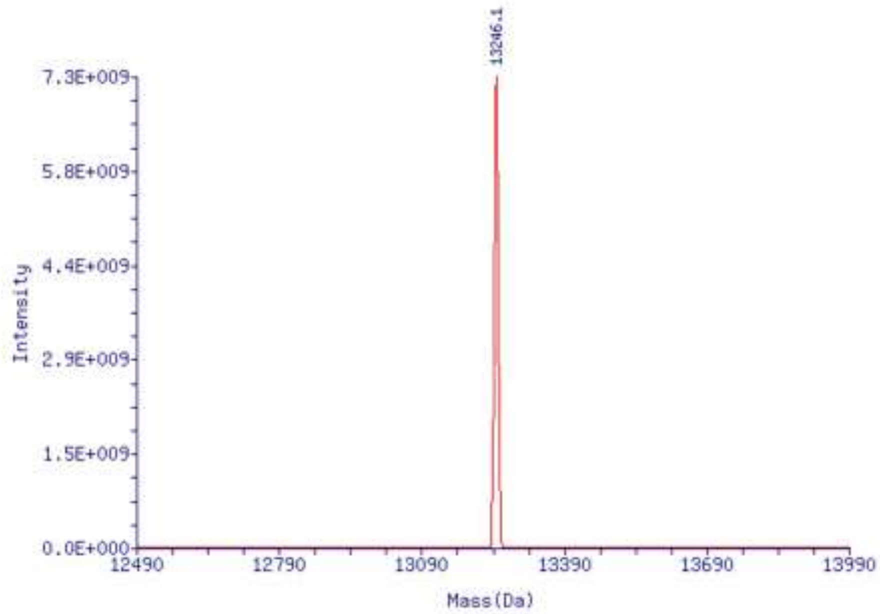

Figure S23. ESI-MS analysis of molecular weight of Ctrl-DNA2-MOR. Calculated: 13246.8 Da. Found: 13246.1 Da.

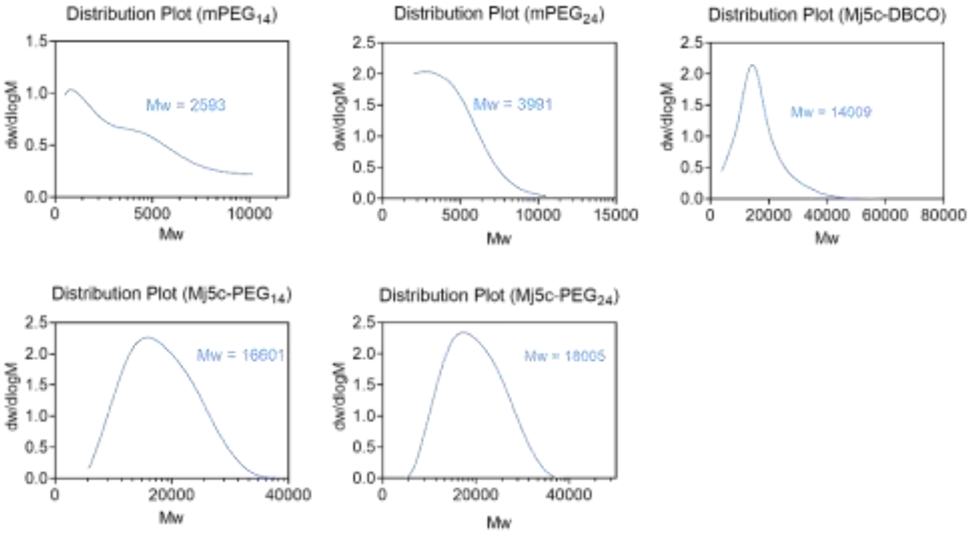

Figure S24. The molecular weight analysis of mPEG$_{14}$, mPEG$_{24}$, Mj5c-DBCO, Mj5c-PEG$_{14}$ and Mj5c-PEG$_{24}$, as determined by gel permeation chromatography.

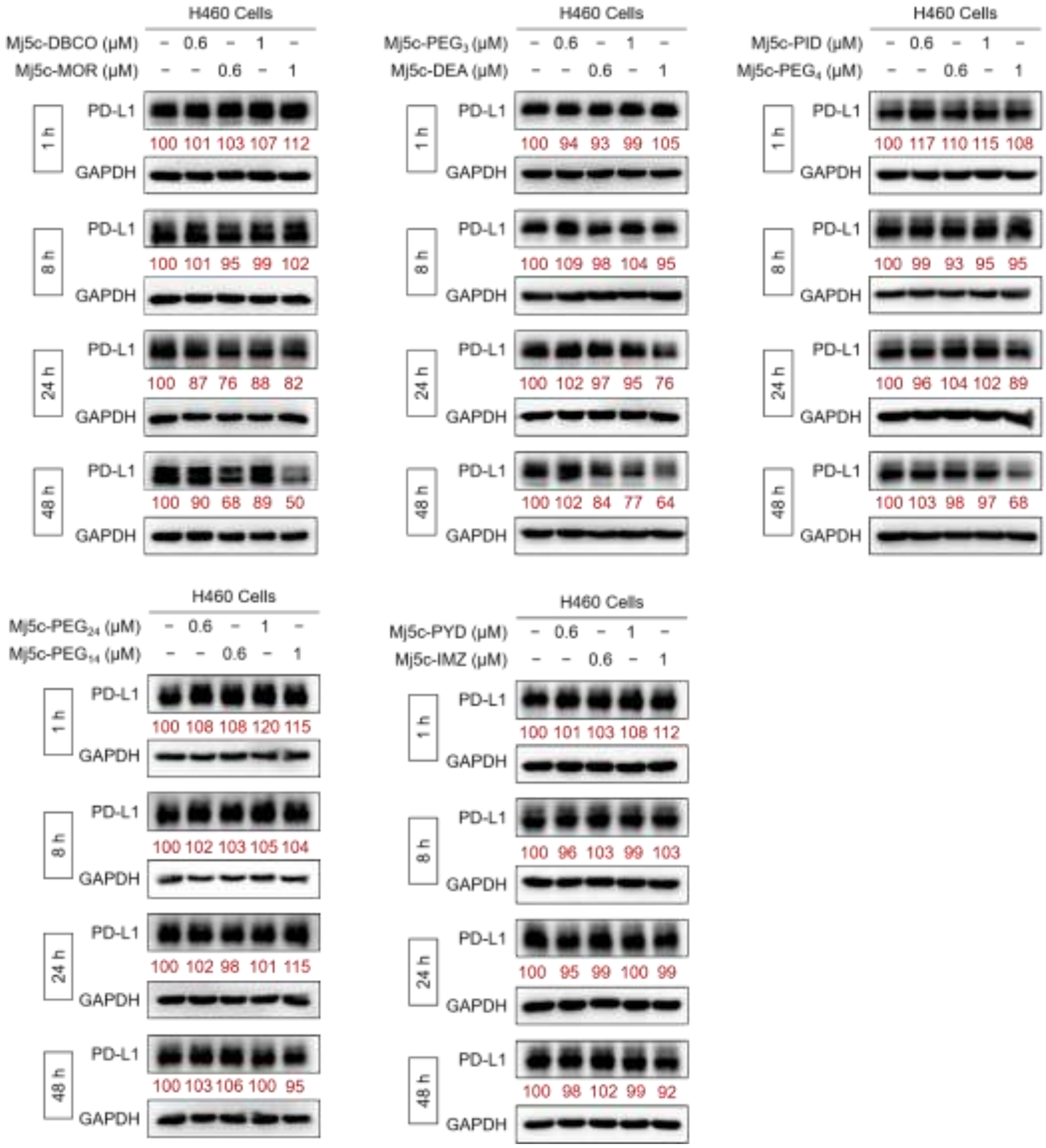

Figure S25. WB analysis of PD-L1 levels in H460 cells after cells were treated with 0.6 μM or 1 μM Mj5c-DBCO or various molecularly built aptamers for 1 h, 8 h, 24 h and 48 h. Relative PD-L1 expression was determined via densitometry and normalized to the loading control.

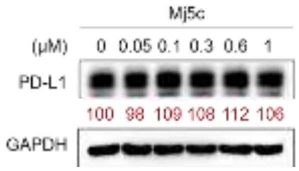

Figure S26. WB analysis of PD-L1 levels in H460 cells after cells were treated with Mj5c at different concentrations for 72 h. Relative PD-L1 expression was determined via densitometry and normalized to the loading control.

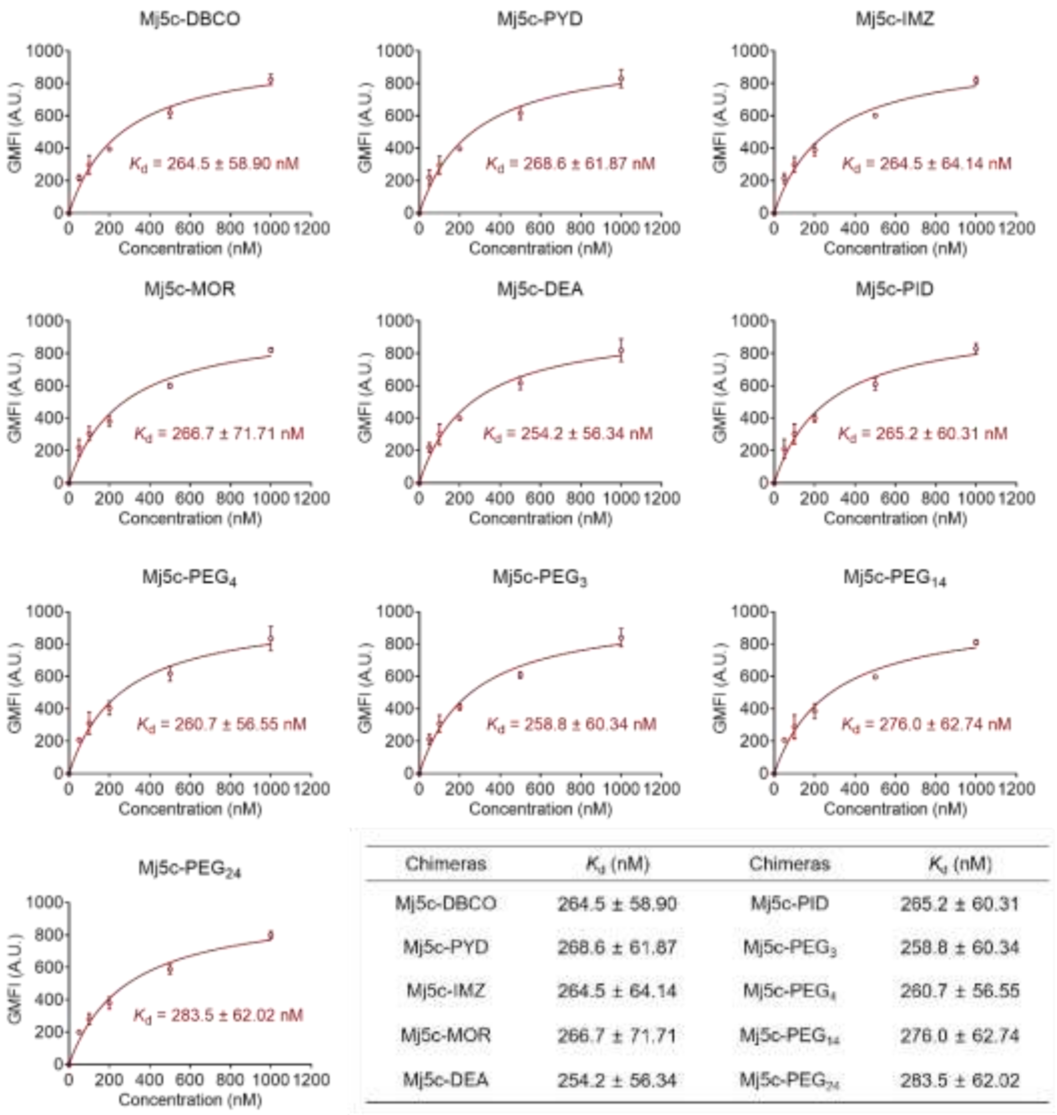

Chimeras	K_d (nM)	Chimeras	K_d (nM)
Mj5c-DBCO	264.5 ± 58.90	Mj5c-PID	265.2 ± 60.31
Mj5c-PYD	268.6 ± 61.87	Mj5c-PEG_3	258.8 ± 60.34
Mj5c-IMZ	264.5 ± 64.14	Mj5c-PEG_4	260.7 ± 56.55
Mj5c-MOR	266.7 ± 71.71	Mj5c-PEG_{14}	276.0 ± 62.74
Mj5c-DEA	254.2 ± 56.34	Mj5c-PEG_{24}	283.5 ± 62.02

Figure S27. Apparent equilibrium dissociation constants (K_ds) of Cy5-labeled Mj5c-DBCO and nine molecularly built aptamers on H460 cells, as determined by flow cytometry. Incubation temperature: 4 °C. Incubation time: 30 min. GMFI indicated the geometric mean fluorescence intensity of cells. K_ds were presented as mean ± SD (n = 3).

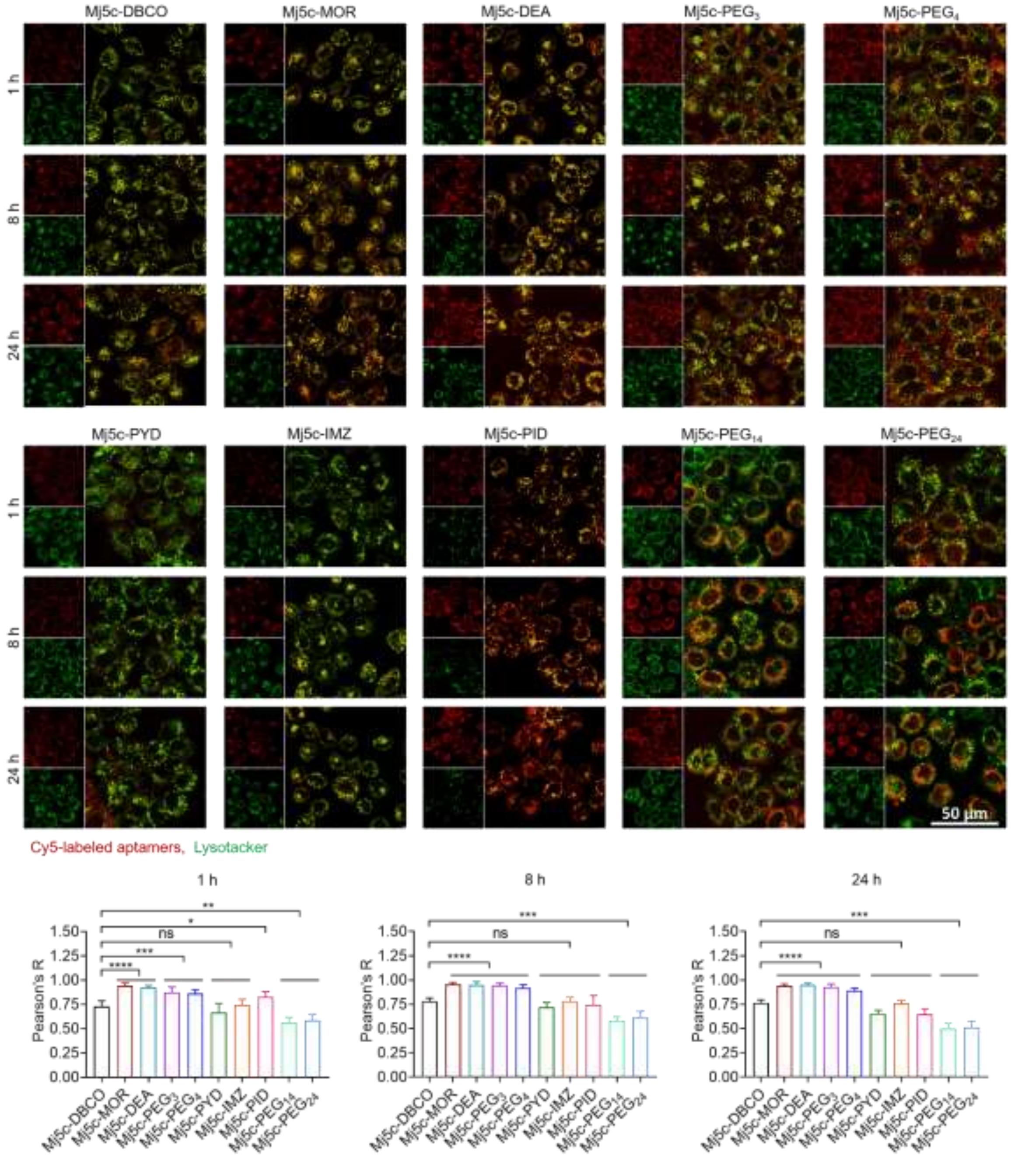

Figure S28. Colocalization analysis between molecularly built aptamers and lysosomes after H460 cells were treated with 1 μM Cy5-labeled molecularly built aptamers (red) for 1 h, 8 h and 24 h. Lysosomes were stained with lysotracker (green). Scale bar = 50 μm. The statistical results of colocalization factor (Pearson's R value) were shown on the bottom panel. All data were presented as mean ± SD (n = 6), calculated from two randomly selected regions per image from three images obtained in three independent experiments. Statistical significance was determined by one-way

ANOVA with a Tukey post hoc test. ns: no significance, *P < 0.05, **P < 0.01, ***P < 0.001, ****P < 0.0001.

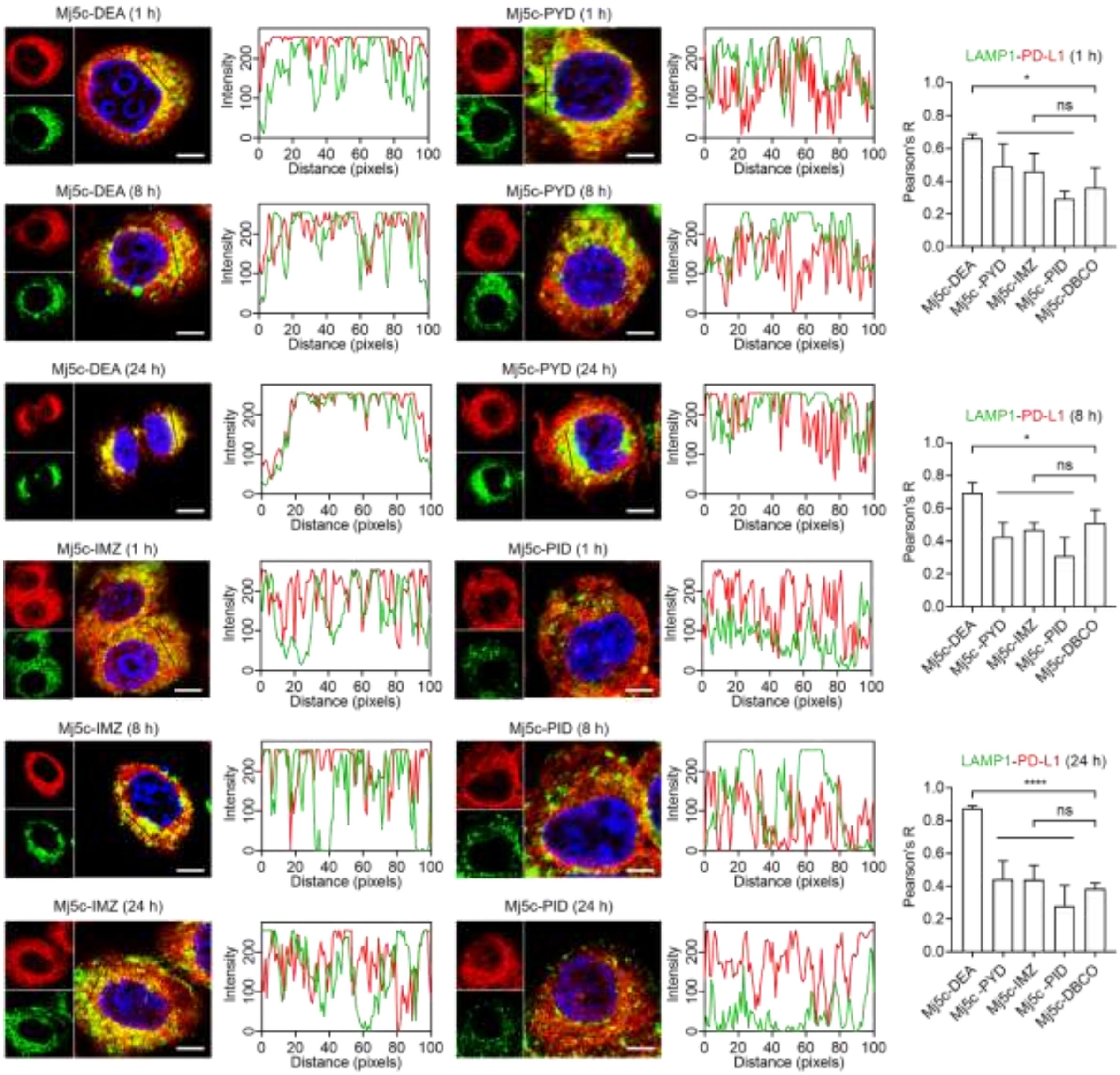

Figure S29. Colocalization analysis of PD-L1 and LAMP1 in H460 cells after treatment with 1 μM different aptameric ligands, which were prepared with azide-DEA, azide-PYD, azide-IMZ, or azide-PID, for 1 h, 8 h, and 24 h. Nuclei were stained by Hoechst 33258 (blue), PD-L1 was detected using an Alexa Fluor594-labeled secondary antibody (red), and LAMP1 was detected using an Alexa Fluor 488-labeled secondary antibody (green). Scale bar = 10 μm. Intensity profiles were generated along the black line, displaying the fluorescence intensity of PD-L1 (red) and LAMP1 (green). The overlay of red and green fluorescence indicated the colocalization of PD-L1

and LAMP1. The right panels showed the statistical analysis of colocalization factor, represented by Pearson's correlation coefficient (R). All data were presented as mean ± SD, based on three independent experimental replicates. Statistical significance was determined by one-way ANOVA with a Tukey post hoc test. ns: no significance, *P < 0.05, ****P < 0.0001.

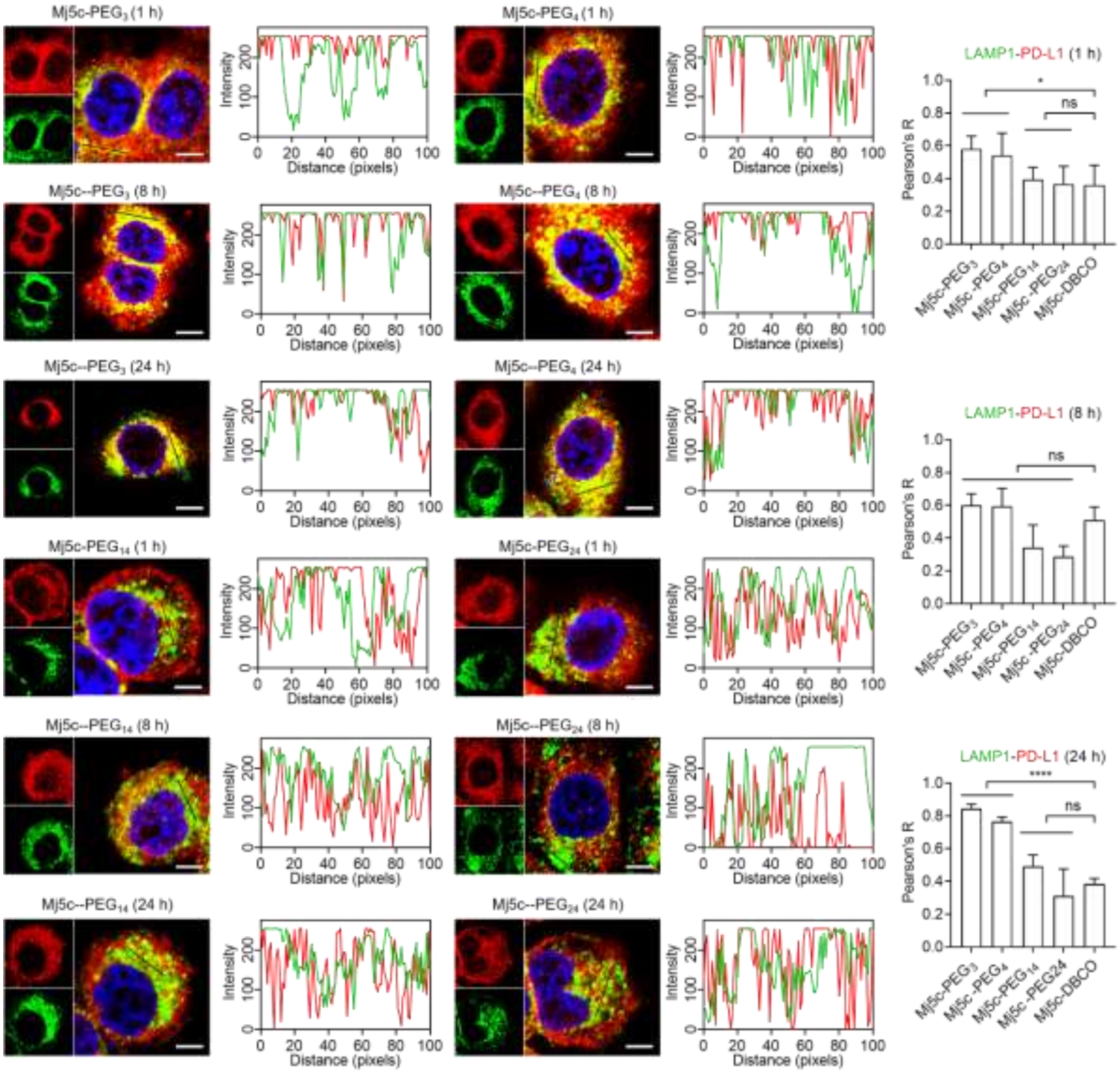

Figure S30. Colocalization analysis of PD-L1 and LAMP1 in H460 cells after treatment with 1 μM different aptameric ligands, which were prepared with azide-PEG_3, azide-PEG_4, azide-PEG_{14}, or azide-PEG_{24}, for 1 h, 8 h, and 24 h. Nuclei were stained by Hoechst 33258 (blue), PD-L1 was detected using an Alexa Fluor594-labeled secondary antibody (red), and LAMP1 was detected using an Alexa Fluor 488-labeled secondary antibody (green). Scale bar = 10 μm. Intensity profiles

were generated along the black line, displaying the fluorescence intensity of PD-L1 (red) and LAMP1 (green). The overlay of red and green fluorescence indicated the colocalization of PD-L1 and LAMP1. The right panels showed the statistical analysis of colocalization factor, represented by Pearson's correlation coefficient (R). All data were presented as mean ± SD, n = 3, three independent images from confocal microscopy. Statistical significance was determined by one-way ANOVA with Tukey's post hoc test. ns: no significance, *P < 0.05, ****P < 0.0001.

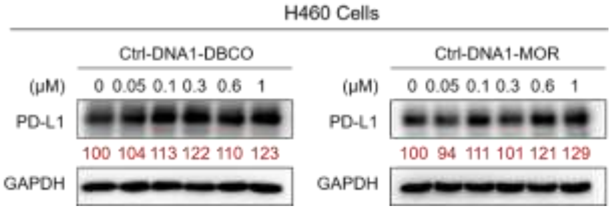

Figure S31. WB analysis of PD-L1 levels in H460 cells after cells were treated with Ctrl-DNA1-DBCO and Ctrl-DNA1-MOR at different concentrations for 72 h. Relative PD-L1 expression was determined via densitometry and normalized to the loading control.

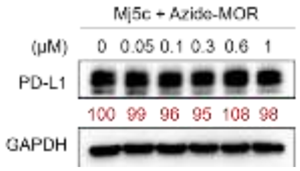

Figure S32. WB analysis of PD-L1 levels in H460 cells after cells were treated with mixture of Mj5c and Azide-MOR with a mole rate of 1:1 at different concentrations for 72 h. Relative PD-L1 expression was determined via densitometry and normalized to the loading control.

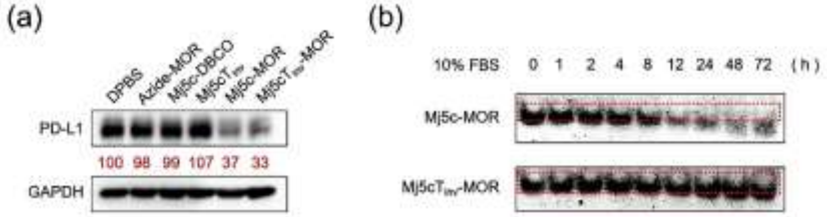

Figure S33. (**a**) WB analysis of PD-L1 levels in H460 cells after cells were treated with 1 μM Azide-MOR, Mj5c-DBCO, Mj5cT$_{inv}$, Mj5c-MOR or Mj5cT$_{inv}$-MOR for 72 h. Relative PD-L1 expression was determined via densitometry and normalized to the loading control. (**b**) Stability

analysis of Mj5c-MOR and Mj5cT$_{inv}$-MOR in 10% FBS, as determined by 8% polyacrylamide gel electrophoresis (PAGE).

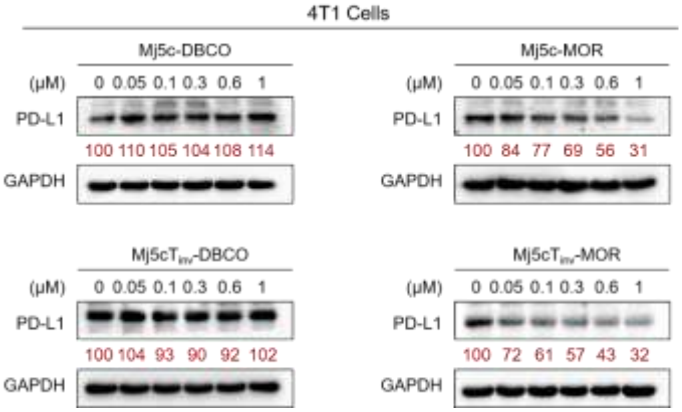

Figure S34. WB analysis of PD-L1 levels in 4T1 cells after cells were treated with Mj5c-DBCO, Mj5c-MOR, Mj5cT$_{inv}$, Mj5cT$_{inv}$-MOR at different concentrations for 48 h. Relative PD-L1 expression was determined via densitometry and normalized to the loading control.

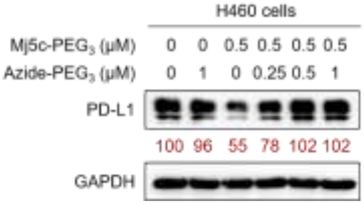

Figure S35. WB analysis of PD-L1 levels in H460 cells after cells were incubated with 0.5 μM Mj5c-PEG$_3$ for 72 h in the absence or presence of azide-PEG$_3$ (0.25, 0.5, 1 μM). Relative PD-L1 expression was determined via densitometry and normalized to the loading control.

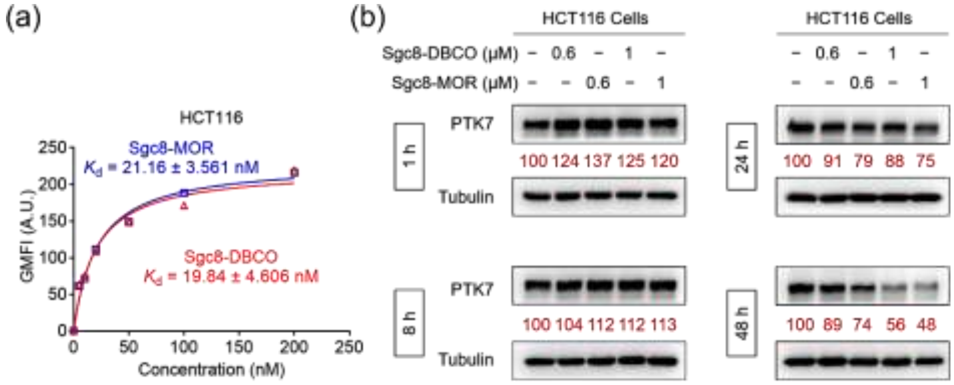

Figure S36. (**a**) Apparent equilibrium dissociation constants (K_ds) of FAM-labeled Sgc8-DBCO and Sgc8-MOR on HCT116 cells, as determined by flow cytometry. Incubation temperature: 4 °C. Incubation time: 30 min. GMFI indicated the geometric mean fluorescence intensity of cells. (**b**) WB analysis of PTK7 levels in HCT116 cells after cells were treated with 0.6 μM or 1 μM Sgc8-DBCO and Sgc8-MOR for 1 h, 8 h, 24 h and 48 h. Relative PTK7 expression was determined via densitometry and normalized to the loading control.

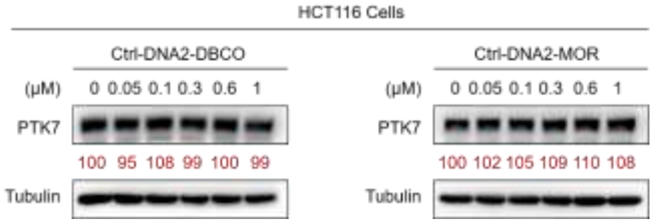

Figure S37. WB analysis of PTK7 levels in HCT116 cells after cells were treated with Ctrl-DNA2-DBCO and Ctrl-DNA2-MOR at different concentrations for 72 h. Relative PTK7 expression was determined via densitometry and normalized to the loading control.

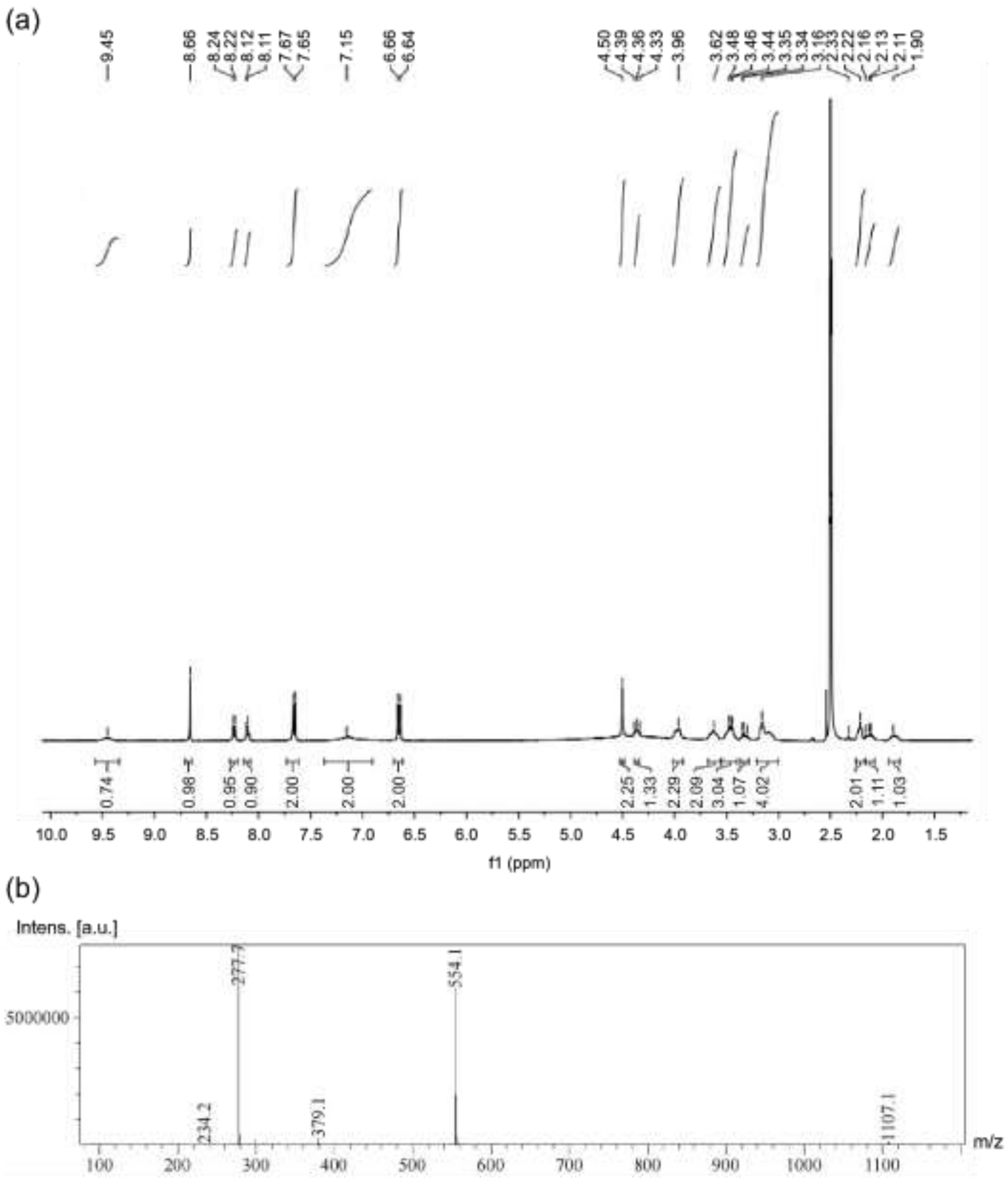

Figure S38. (**a**) NMR spectrum of FA-MOR. ^{1}H NMR (400 MHz, DMSO) δ 9.45 (s, 1H), 8.66 (s, 1H), 8.23 (d, J = 8.0 Hz, 1H), 8.11 (t, J = 5.8 Hz, 1H), 7.66 (d, J = 8.8 Hz, 2H), 7.15 (s, 2H), 6.65 (d, J = 8.8 Hz, 2H), 4.50 (s, 2H), 4.36 (s, 1H), 3.96 (s, 2H), 3.62 (s, 2H), 3.53 – 3.40 (m, 3H), 3.37 – 3.29 (m, 1H), 3.16 (s, 4H), 2.22 (s, 2H), 2.12 (d, J = 7.1 Hz, 1H), 1.90 (s, 1H). (**b**) LC-MS analysis of the molecular weight of FA-MOR.

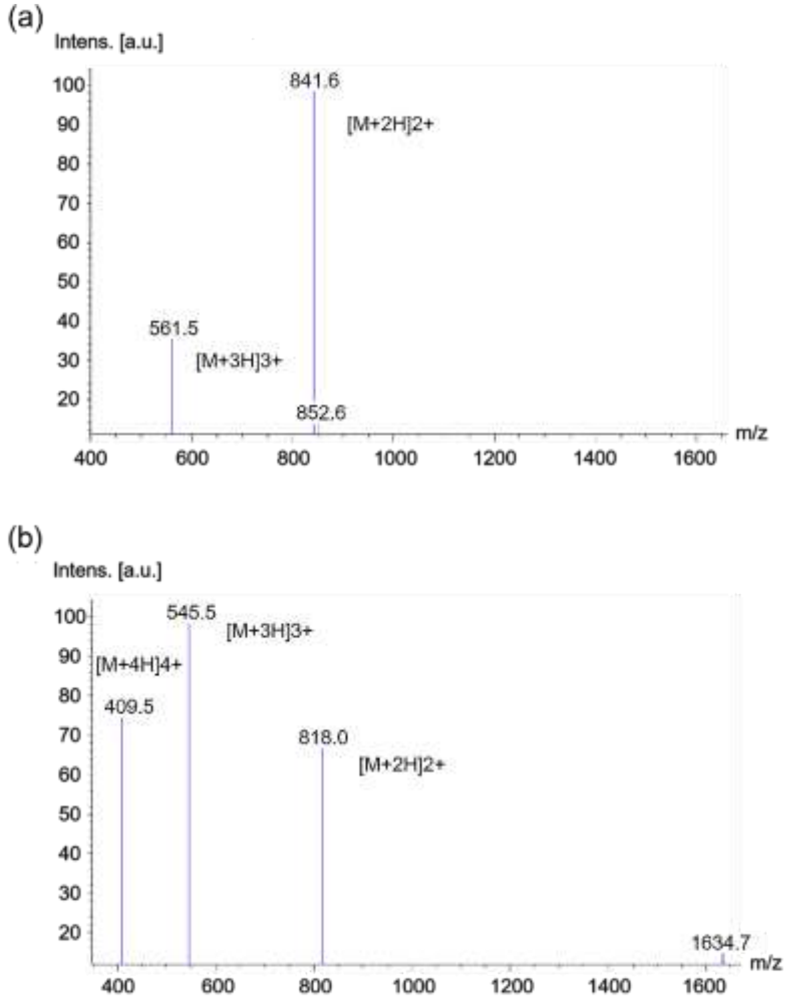

Figure S39. (**a**) Mass spectrometry analysis of molecular weight of GE11-MOR (MW = 1681.85). (**b**) Mass spectrometry analysis of molecular weight of HW12-MOR (MW = 1633.92).

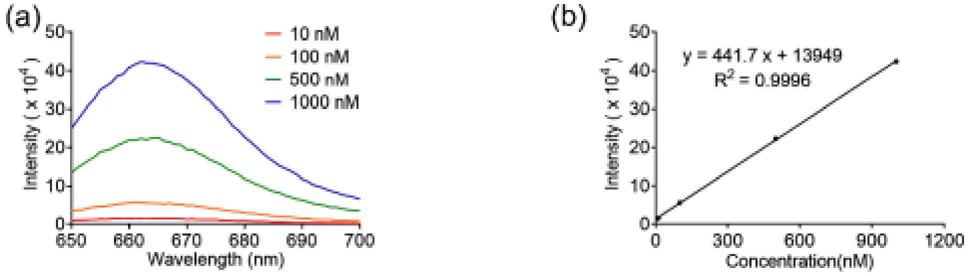

Figure S40. Identification of the number of modified molecules on the CTX. (**a**) Fluorescence emission spectra at wavelengths 650 nm - 700 nm of Cy5 molecules at different concentrations. (**b**) Standard calibration curve for determining the number of Cy5 on $NB_{PD\text{-}L1}$ and CTX.

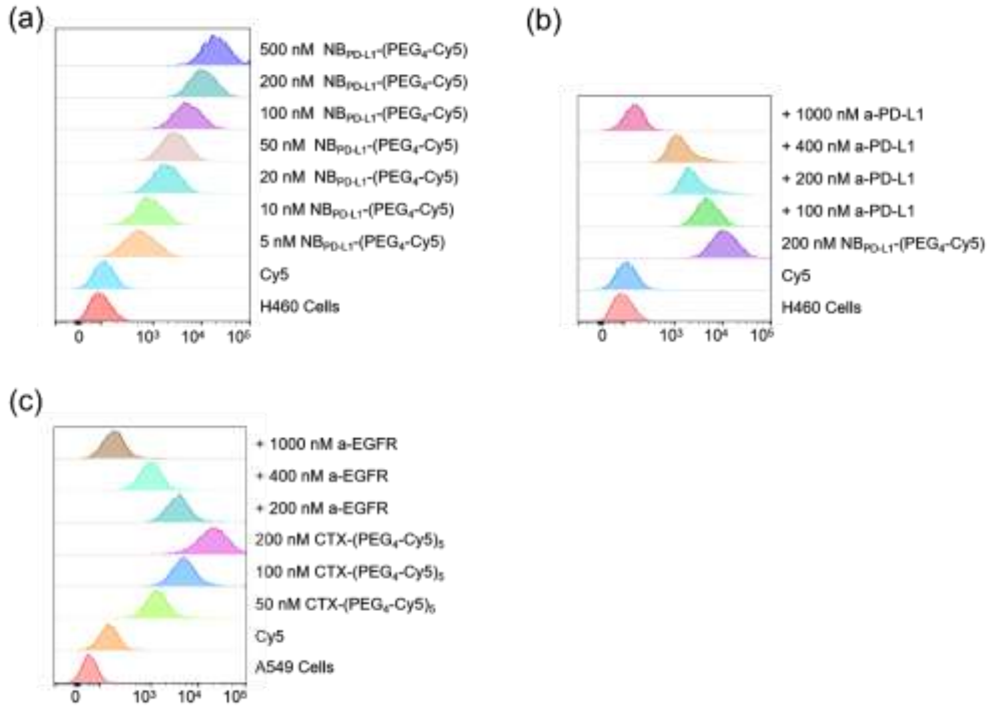

Figure S41. (**a**) Analysis of the binding ability of NB$_{PD-L1}$-(PEG$_4$-Cy5) on H460 cells by flow cytometry. (**b**) Competition analysis between NB$_{PD-L1}$-(PEG$_4$-Cy5) and a-PD-L1 on H460 cells. (**c**) Competition analysis between CTX-(PEG$_4$-Cy5)$_5$ and anti-EGFR antibody (a-EGFR) on A549 cells. Incubation temperature: 4°C. Incubation time: 30 min.

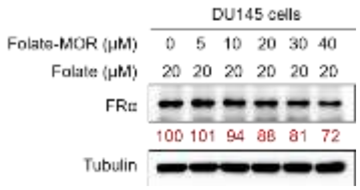

Figure S42. WB analysis of FRα levels in DU145 cells after cells were treated with Folate-MOR at different concentrations in the presence of 20 μM Folate for 72 h. Relative FRα expression was determined via densitometry and normalized to the loading control.

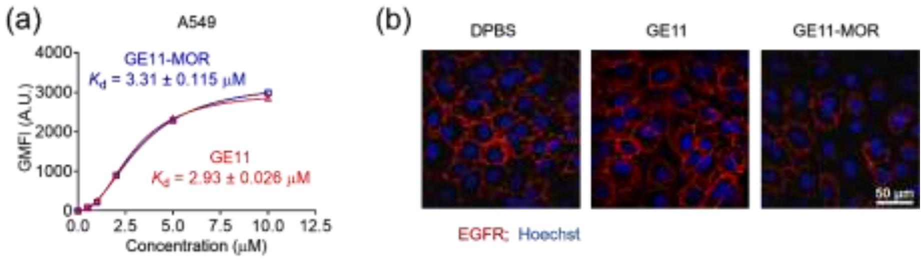

Figure S43. (**a**) Apparent equilibrium dissociation constants (K_ds) of FITC-labeled GE11 and GE11-MOR on A549 cells, as determined by flow cytometry. Incubation temperature: 4 °C. Incubation time: 30 min. GMFI indicated the geometric mean fluorescence intensity of cells. (**b**) Immunofluorescence confocal imaging of EGFR (Anti-Rabbit IgG Alexa Fluor 647, red) after A549 cells treated with 5 μM GE11 or GE11-MOR for 72 h. Nuclei was stained by Hoechst 33258 (blue). Scale bar = 50 μm.

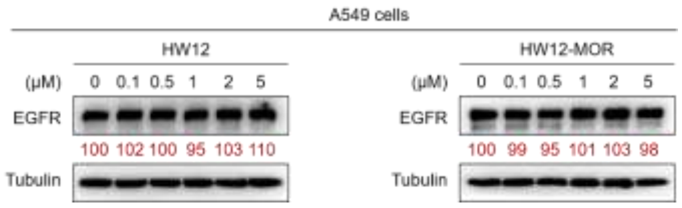

Figure S44. WB analysis of EGFR levels in A549 cells after cells were treated with control peptide HW12 and HW12-MOR at different concentrations for 72 h. Relative EGFR expression was determined via densitometry and normalized to the loading control.

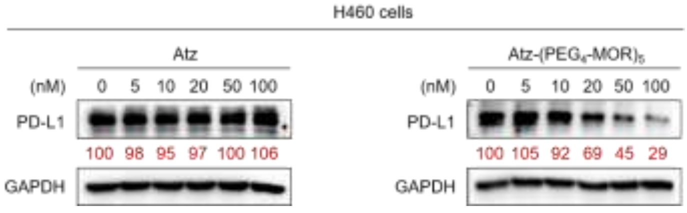

Figure S45. WB analysis of PD-L1 levels in H460 and 4T1 cells after H460 cells were treated with Atz and Atz-(PEG_4-MOR)$_5$ at different concentrations for 48 h. Relative PD-L1 expression was determined via densitometry and normalized to the loading control.

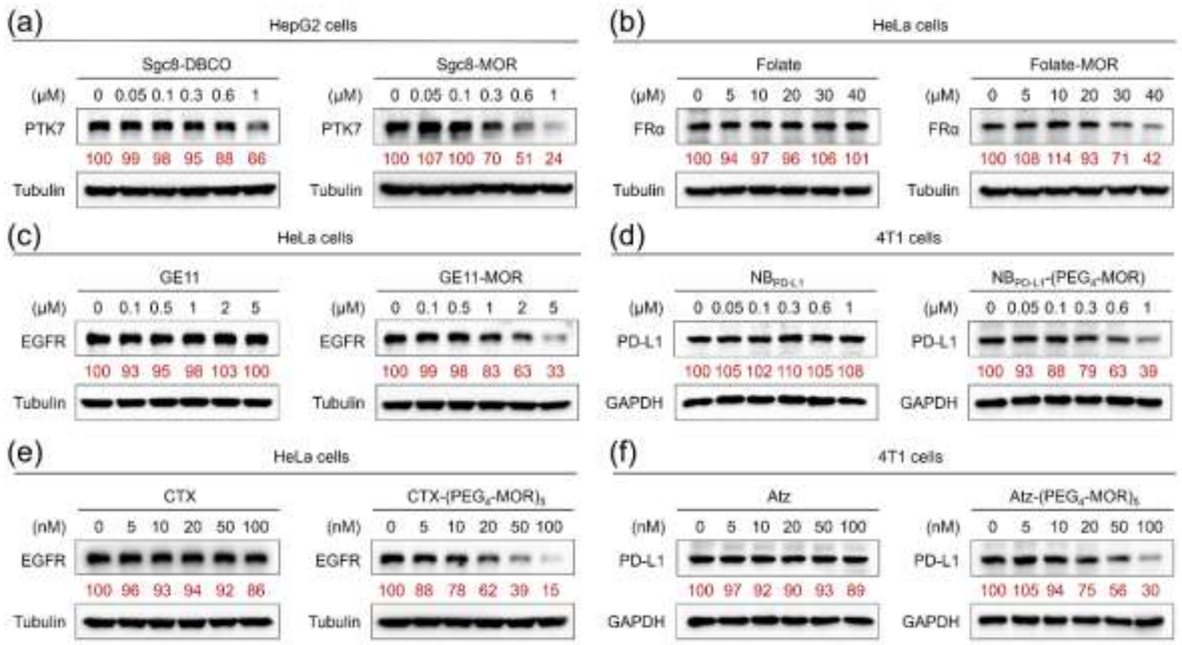

Figure S46. (**a**) WB analysis of PTK7 levels in HepG2 cells after cells were treated with Sgc8 and Sgc8-MOR at different concentrations for 72 h. (**b**) WB analysis of FRα levels in HeLa cells after cells were treated with Folate and Folate -MOR at different concentrations for 72 h. (**c**) WB analysis of EGFR levels in HeLa cells after cells were treated with GE11 and GE11-MOR at different concentrations for 72 h. (**d**) WB analysis of PD-L1 levels in 4T1 cells after cells were treated with NB$_{PD\text{-}L1}$ and NB$_{PD\text{-}L1}$-(PEG$_4$-MOR) at different concentrations for 72 h. (**e**) WB analysis of EGFR levels in HeLa cells after cells were treated with CTX and CTX-(PEG$_4$-MOR)$_5$ at different concentrations for 48 h. (**f**) WB analysis of PD-L1 levels in 4T1 cells after cells were treated with Atz and Atz-(PEG$_4$-MOR)$_5$ at different concentrations for 48 h. Relative target protein expression was determined via densitometry and normalized to the loading control.

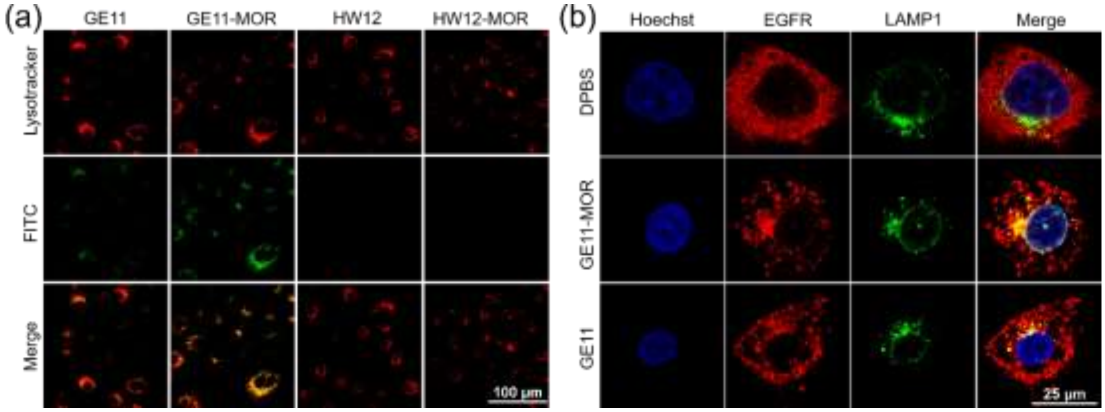

Figure S47. (**a**) Colocalization analysis of FITC-labeled peptides and lysosomes after A549 cells were treated with 5 μM FITC-labeled GE11, GE11-MOR, HW12 and HW12-MOR (green) for 24 h. Lysosomes were stained with lysotracker (red). Scale bar = 100 μm. (**b**) Colocalization analysis between EGFR (Anti-Rabbit IgG Alexa Fluor 594, red) and LAMP1 (Anti-Mouse IgG Alexa Fluor 488, green) after A549 cells were treated with 5 μM GE11 or GE11-MOR for 24 h. Nuclei was stained by Hoechst 33258 (blue). Scale bar = 25 μm.

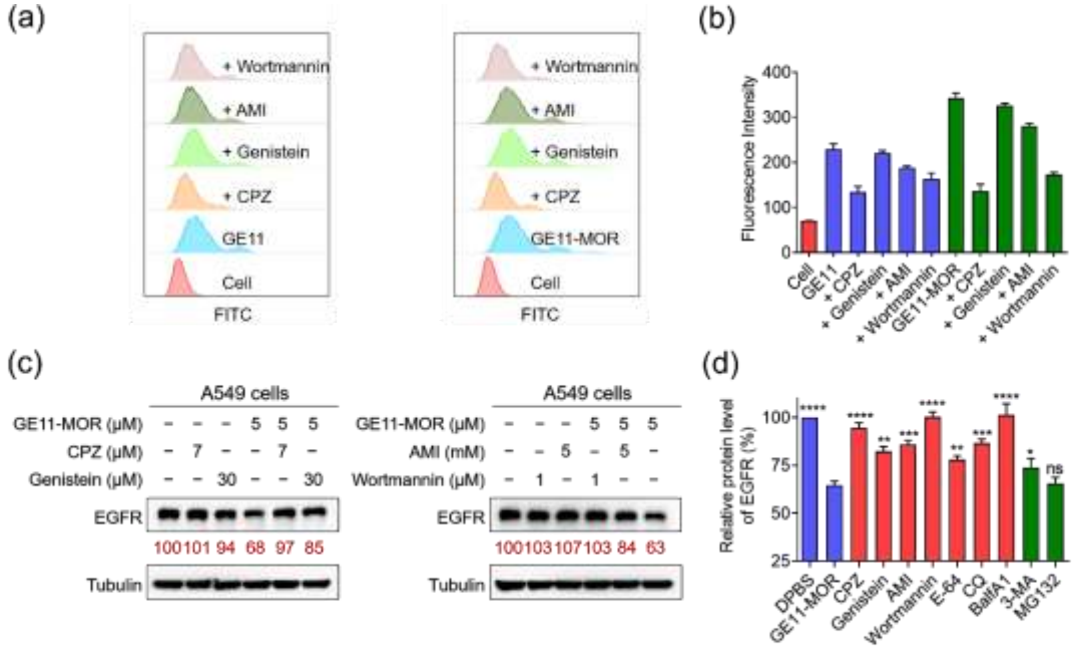

Figure S48. (**a**) Flow cytometry analysis of the endocytosis of FITC-labeled GE11 or GE11-MOR in A549 cells after cells were incubated 5 μM peptides for 8 h in the absence or presence various endocytosis inhibitors. Incubation time: 8 h, temperature: 37 °C, chlorpromazine (CPZ): 7 μM, genistein: 30 μM, amiloride (AMI): 5 mM, Wortmannin: 1 μM. (**b**) Geometric mean fluorescence intensity (GMFI) statistics in (**a**). (**c**) WB analysis of EGFR levels in A549 cells after cells were incubated with 5 μM GE11-MOR for 72 h in the absence or presence of various inhibitors. Relative EGFR expression was determined via densitometry and normalized to the loading control. (**d**) Relative value statistics in (**c**). All data were presented as mean ± SD, n = 3. Statistical significance was determined by one-way ANOVA with a Tukey post hoc test. ns: no significance, *P < 0.05, **P < 0.01, ***P < 0.001, ****P < 0.0001.

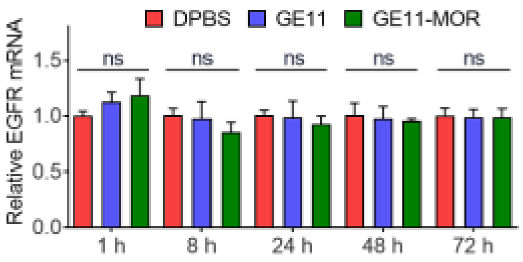

Figure S49. RT-qPCR analysis of EGFR gene levels in A549 cells treated with DPBS, 5 μM GE11 or 5 μM GE11-MOR for 1 h, 8 h, 24 h, 48 h or 72 h. All data were presented as mean ± SD, n = 4. Statistical significance was determined by one-way ANOVA with a Tukey post hoc test. ns: no significance.

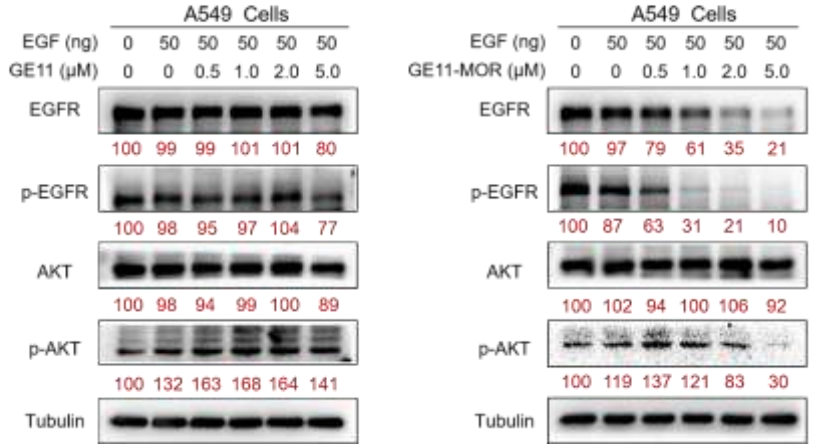

Figure S50. WB analysis of the levels of EGFR, p-EGFR, AKT and p-AKT in A549 cells after A549 cells were stimulated with 50 ng EGF for 2 h, followed by incubation with GE11 or GE11-MOR at different concentrations for 72 h. Relative target protein expression was determined via densitometry and normalized to the loading control.

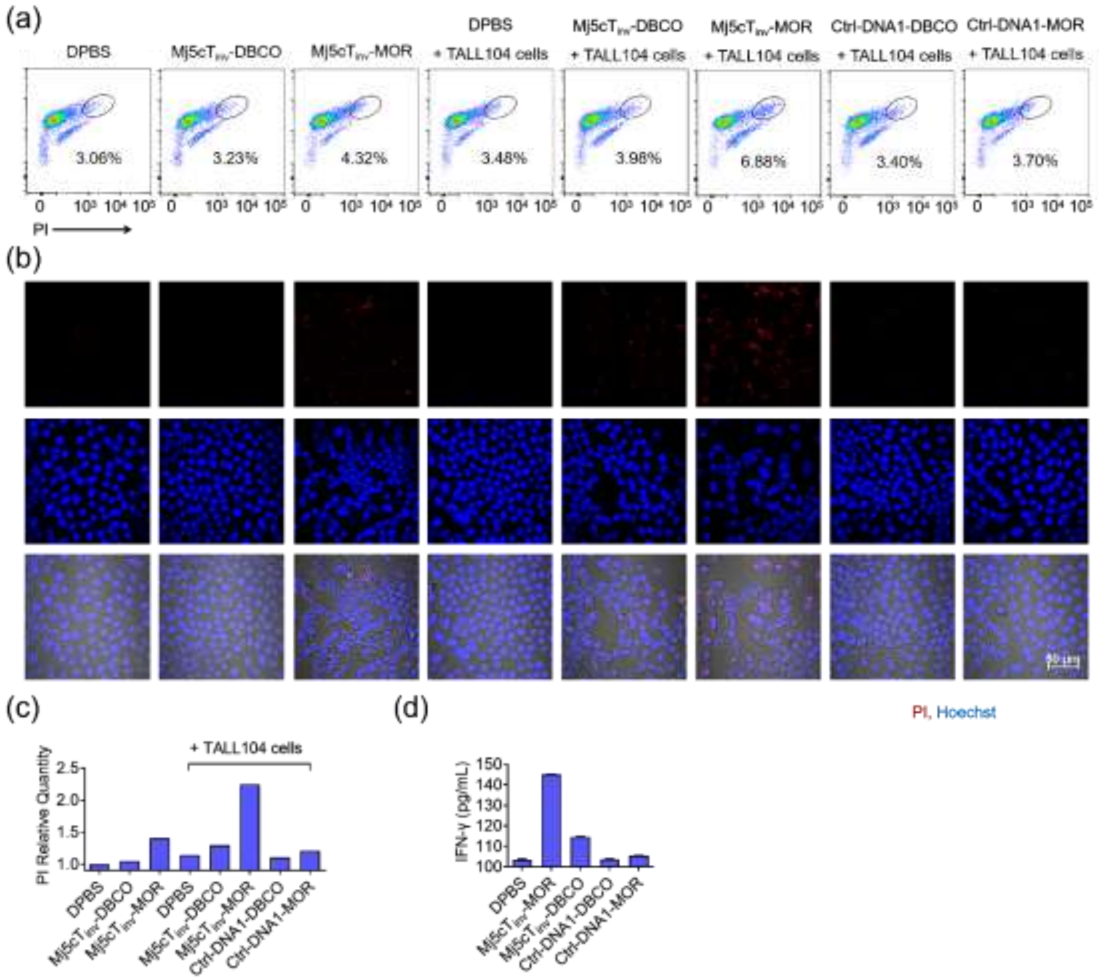

Figure S51. (**a**) Flow cytometric of H460 cells stained with propidium iodide (PI) after cells were treated with or without 1 μM molecularly built aptamers for 72 h, followed by 2-h incubation with TALL104 cells. (**b**) Fluorescence imaging analysis of H460 cells stained with PI (red) in (**a**). Nuclei were stained by Hoechst 33258 (blue). Scale bar = 50 μm. (**c**) Statistical data of the relative quantity of PI-positive cells in (**b**). (**d**) IFN-γ levels in the culture supernatant after the incubation time with TALL104 cells was extended to 24 h in (**a**), as determined by ELISA. All data were presented as mean ± SD, n = 3.

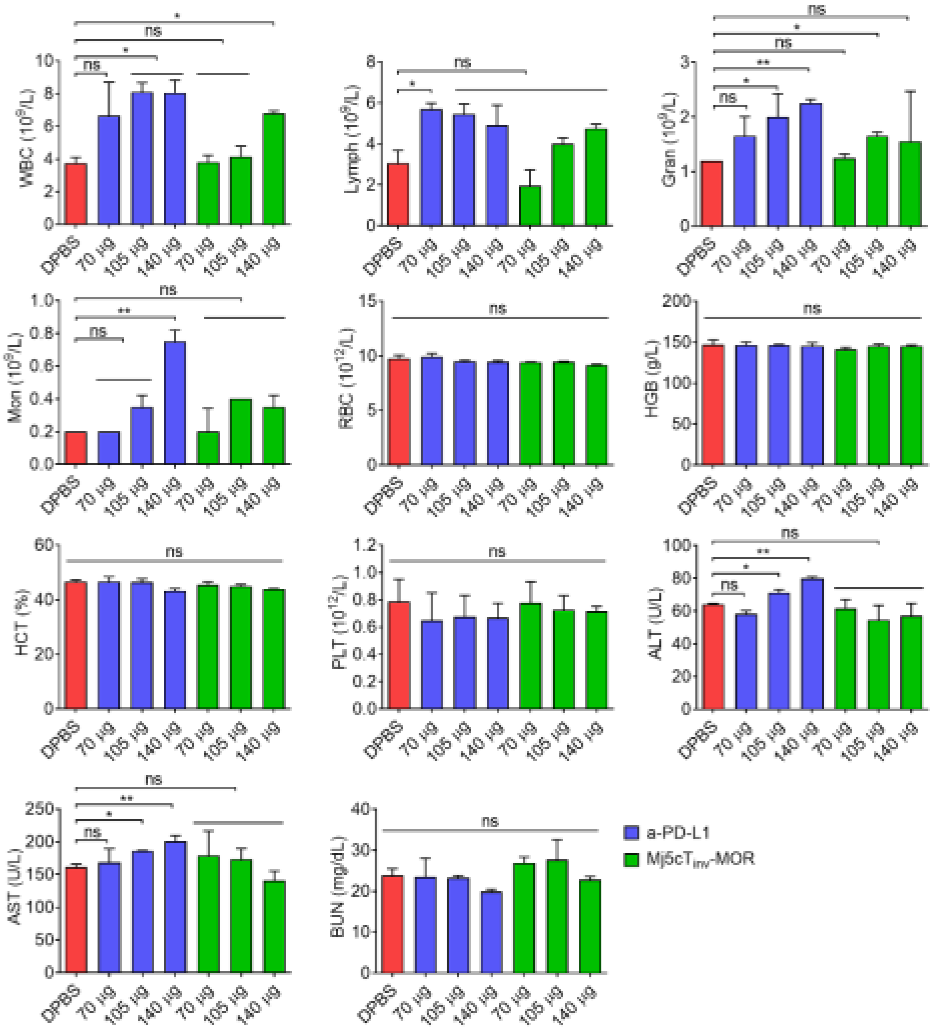

Figure S52. Blood routine test and blood biochemical analysis of healthy and immunocompetent BALB/c female mice at 24 h post injection of Mj5cT$_{inv}$-MOR or a-PD-L1. WBC: white blood cell, RBC: red blood cell, Lymph: lymphocyte, Gran: neutrophil, Mon: monocyte, HGB: hemoglobin, HCT: hematocrit, PLT: platelet, ALT: alanine aminotransferase, AST: aspartate aminotransferase, BUN: blood urea nitrogen. All data were presented as mean ± SD, n = 3. Statistical significance was determined by one-way ANOVA with a Tukey post hoc test. ns: no significance, *$P < 0.05$, **$P < 0.01$.

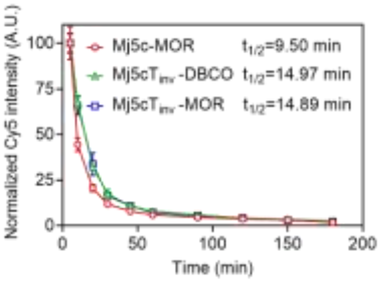

Figure S53. In vivo circulation half-life of Mj5c-MOR, Mj5cT$_{inv}$-DBCO and Mj5cT$_{inv}$-MOR in healthy BALB/c female mice. The injection dosage was 5 nmol. All data were presented as mean ± SD, n = 3.

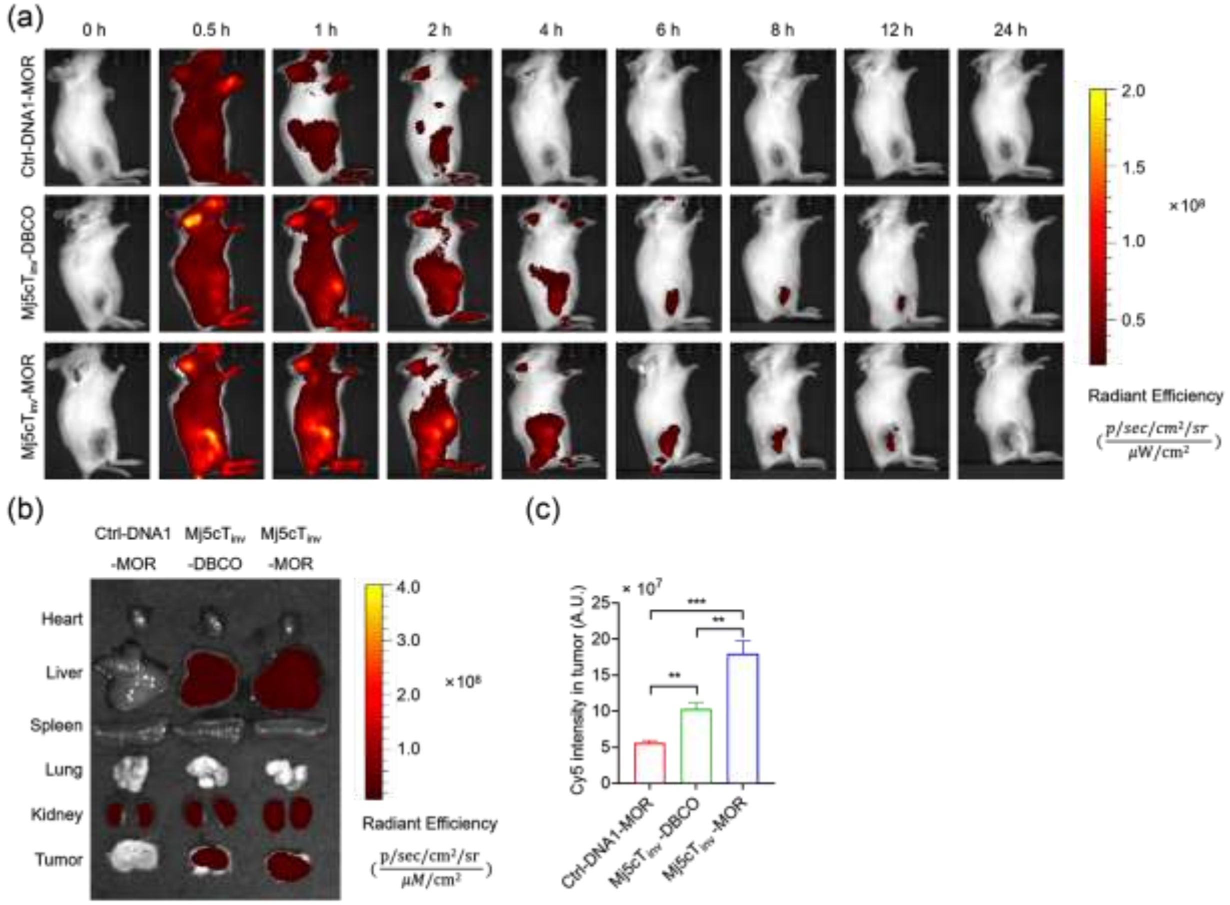

Figure S54. (**a**) Whole-body fluorescence imaging of 4T1 tumor-bearing mice at different time points post injection. The injection dose of Cy5-labeled DNA was 5 nmol. (**b**) The fluorescence imaging of various organs at 48 h post injection. (**c**) Quantitative statistics of fluorescence intensity in tumors in each group. All data were presented as mean ± SD, n = 3. Statistical significance was

determined by one-way ANOVA with a Tukey post hoc test. ns: no significance, *P < 0.05, **P < 0.01, ***P < 0.001, ****P < 0.0001.

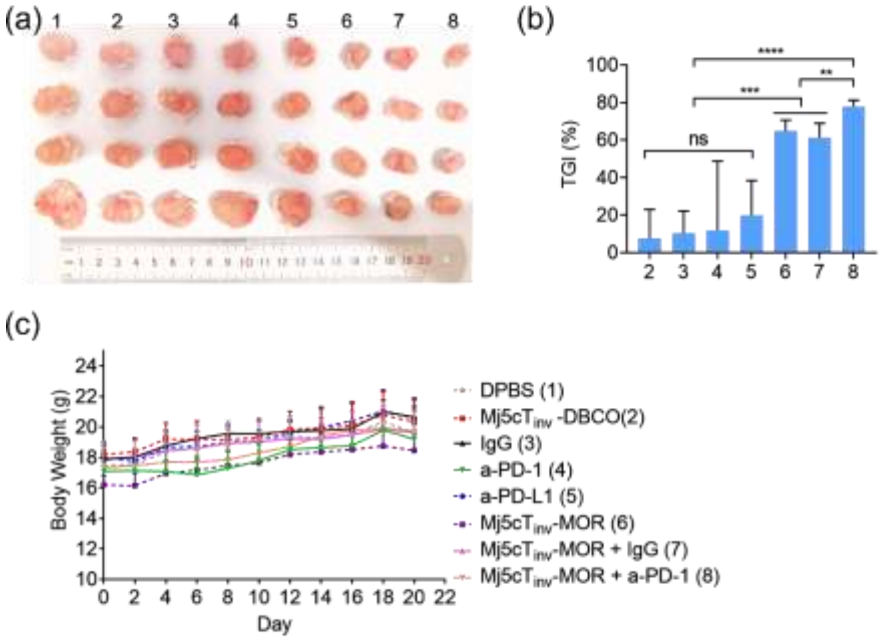

Figure S55. (**a**) Photograph of 4T1 tumors after mice were received different treatments. Group 1: DPBS, 2: Mj5cT$_{inv}$-DBCO, 3: IgG, 4: a-PD-1, 5: a-PD-L1, 6: Mj5cT$_{inv}$-MOR, 7: Mj5cT$_{inv}$-MOR plus IgG, 8: Mj5cT$_{inv}$-MOR plus a-PD-1. (**b**) Tumor growth inhibition rate of each group relative to the DPBS-treated mice. Statistical significance was determined by one-way ANOVA with a Tukey post hoc test. ns: no significance, **P < 0.01, ***P < 0.001, ****P < 0.0001. (**c**) Body weight curves of 4T1 tumor-bearing BALB/c female mice during the 21-day study period, in which mice were received different treatments. All data were presented as mean ± SD, n = 5.

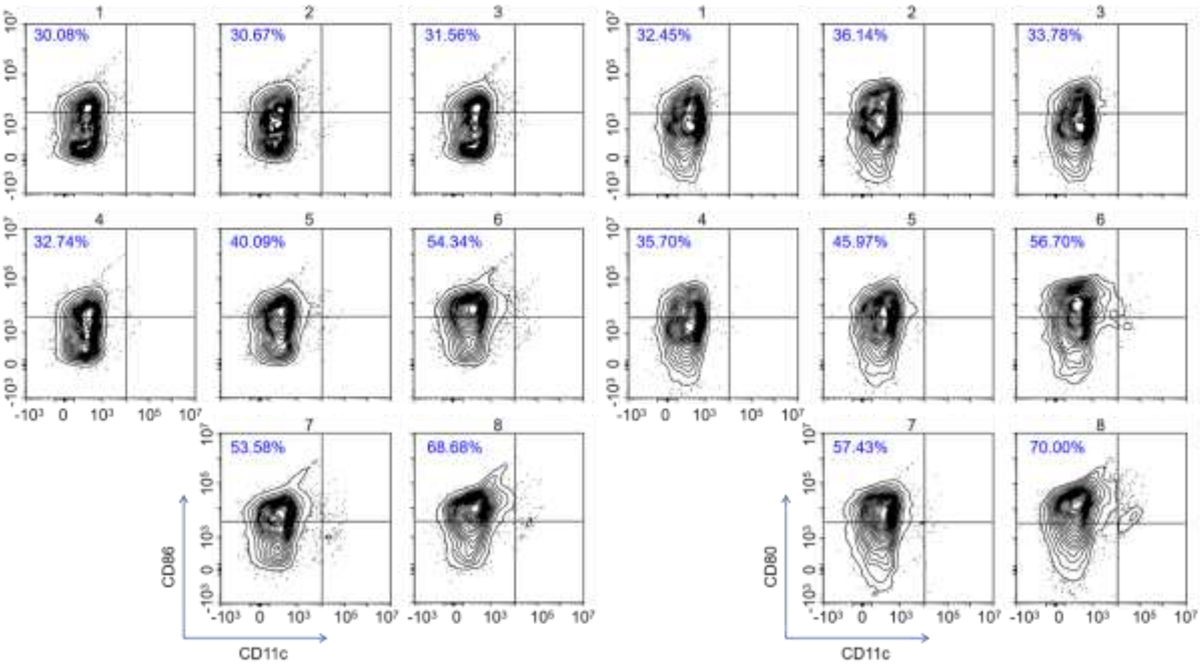

Figure S56. Flow cytometric assay of the mature DCs from the draining lymph nodes. Cells were stained with PE-labeled anti-CD11c antibody and PE-Cy7-labeled anti-CD86/CD80 antibody.

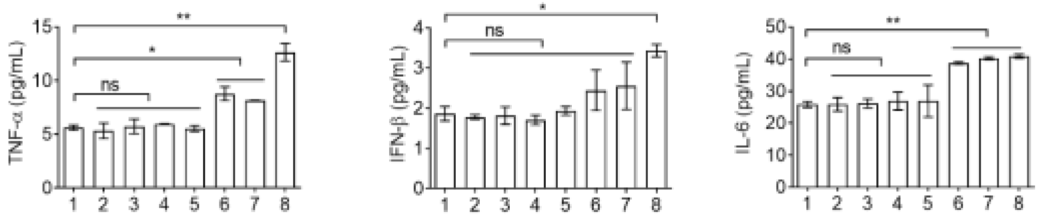

Figure S57. The immune factors in serum of 4T1 tumor-bearing mice after receiving different treatments. TNF-α: tumor necrosis factor α, IFN-β: interferon-β, IL-6: interleukin-6. All data were presented as mean ± SD, n = 3. Statistical significance was determined by one-way ANOVA with a Tukey post hoc test. ns: no significance, *P < 0.05, **P < 0.01.

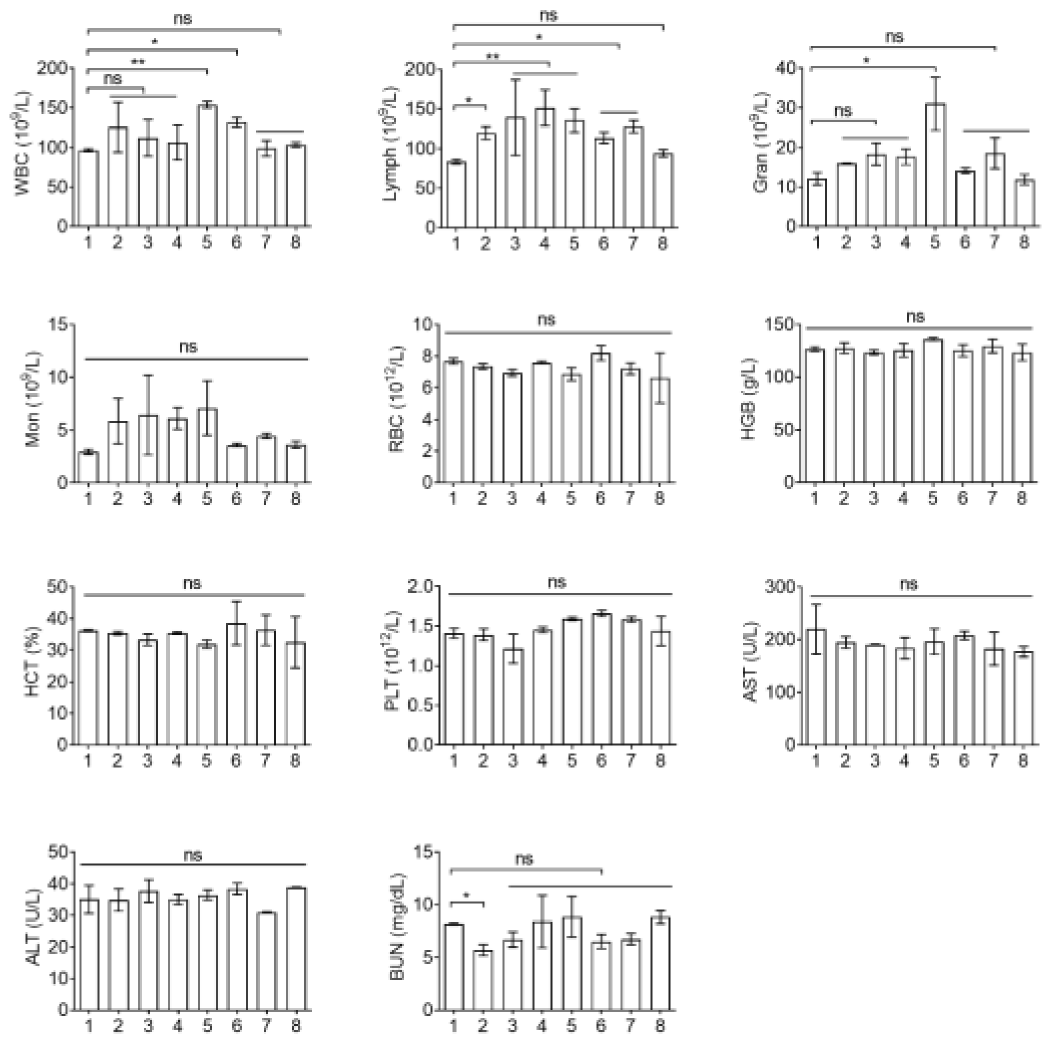

Figure S58. The blood routine test and blood biochemical analysis of 4T1 tumor-bearing mice after receiving different treatments. WBC: white blood cell, Lymph: lymphocyte, Gran: neutrophil, Mon: monocyte, RBC: red blood cell, HGB: hemoglobin, HCT: hematocrit, PLT: platelet, AST: aspartate aminotransferase, ALT: alanine aminotransferase, BUN: blood urea nitrogen. All data were presented as mean ± SD, n = 3. Statistical significance was determined by one-way ANOVA with a Tukey post hoc test. ns: no significance, *P < 0.05, **P < 0.01. The stability of CTX in 10% FBS and in mouse circulation was analyzed.

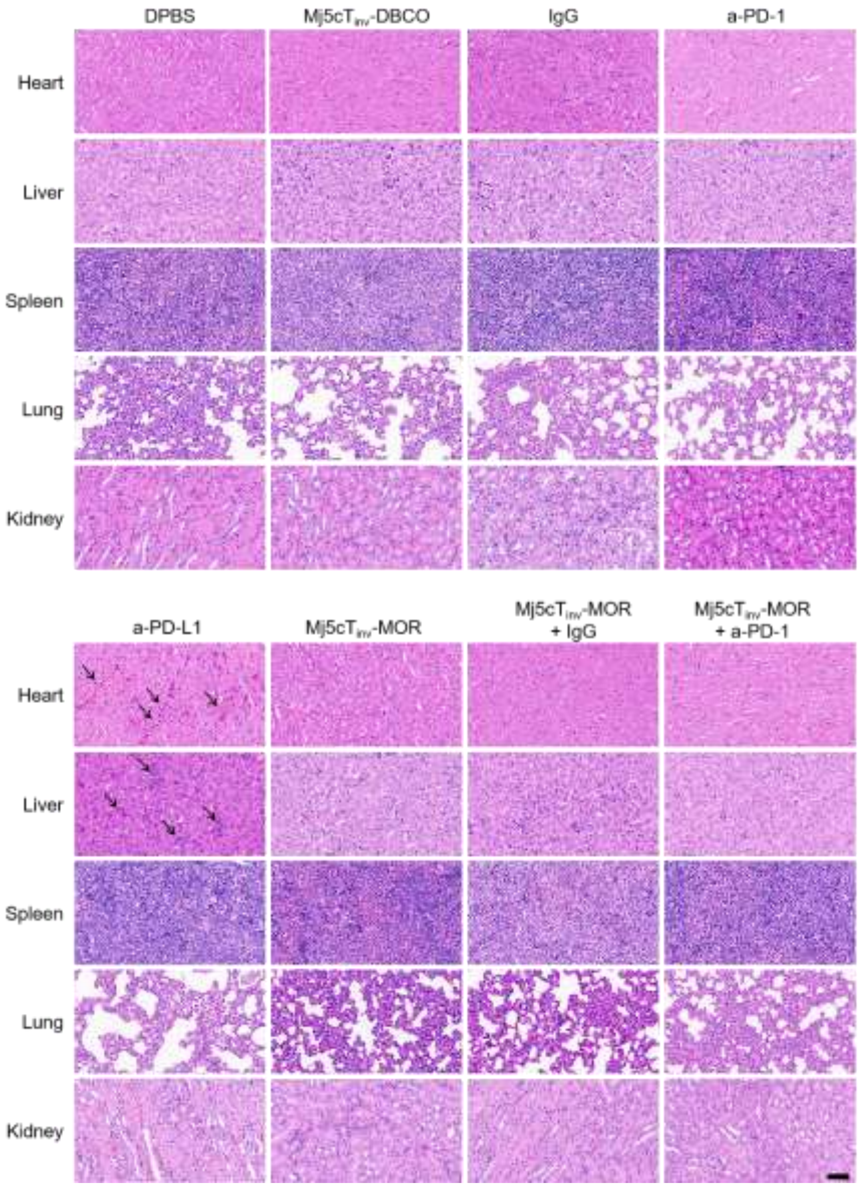

Figure S59. H&E staining analysis of heart, liver, spleen, lung and kidney of 4T1 tumor-bearing mice after receiving different treatments. The arrow points to the organ damage of anti-PD-L1 antibody. Scale bar = 50 μm.

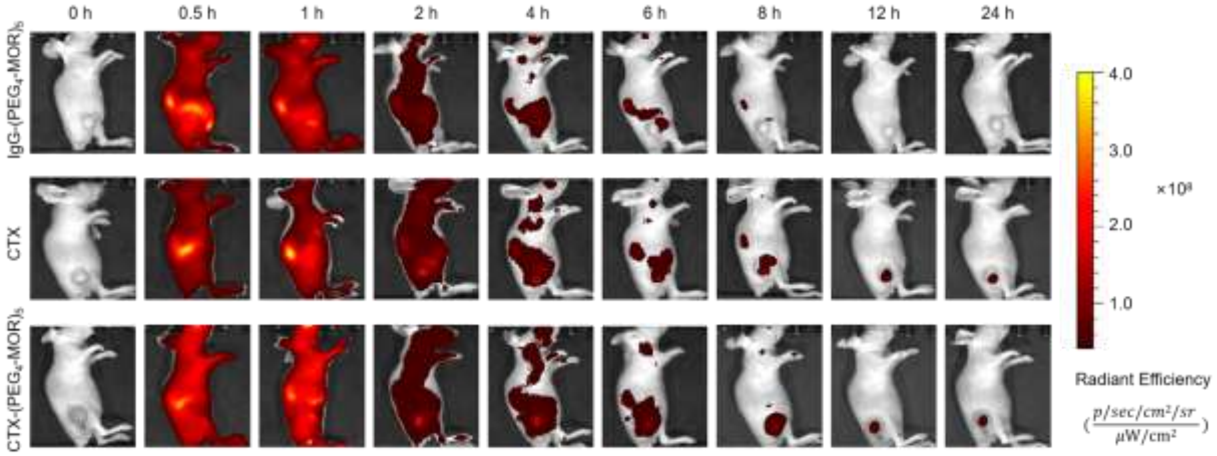

Figure S60. Whole-body fluorescence imaging of A549 tumor-bearing mice at different time points post injection. The injection dose of Cy5-labeled antibody was 80 μg.

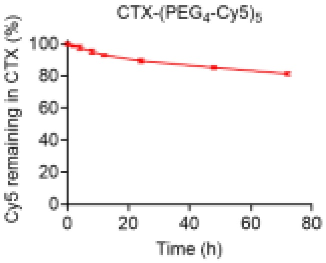

Figure S61. Analysis of the stability of CTX-(PEG_4-MOR)$_5$ in healthy BALB/c female nude mice at different time points post injection. The injection dosage was 80 μg.

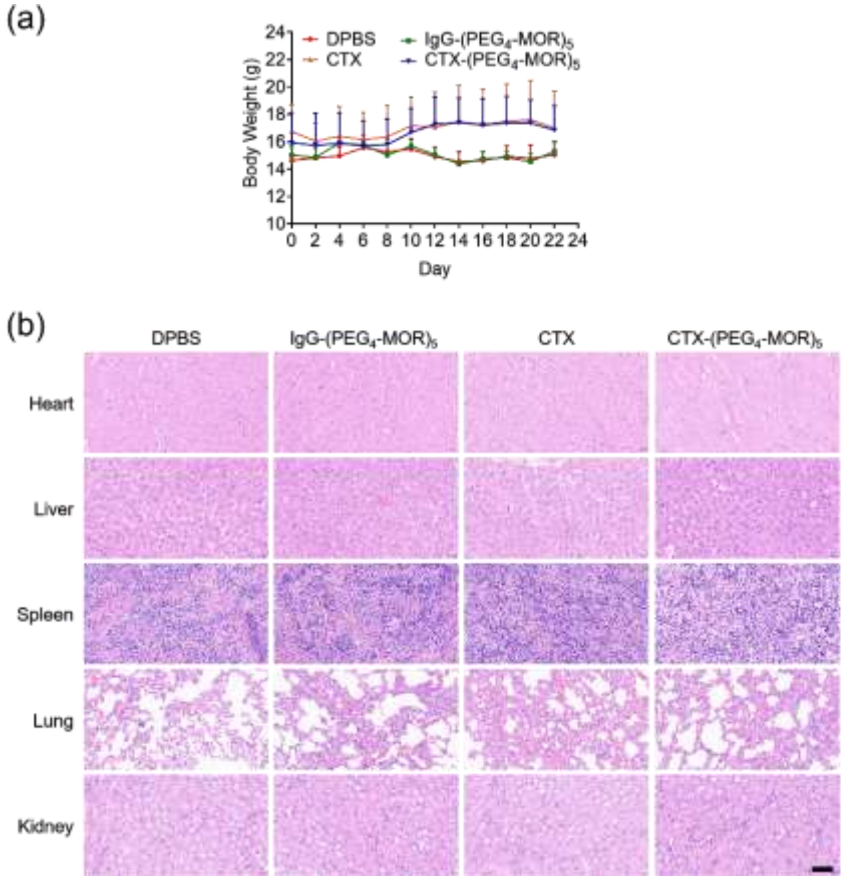

Figure S62. (**a**) Body weight curves of A549 tumor-bearing BALB/c nude mice during the 23-day study period, in which mice were received different treatments. All data were presented as mean ± SD, n = 5. (**b**) Representative H&E staining images of heart, liver, spleen, lung and kidney in each group receiving different treatments. Scale bar = 50 μm.

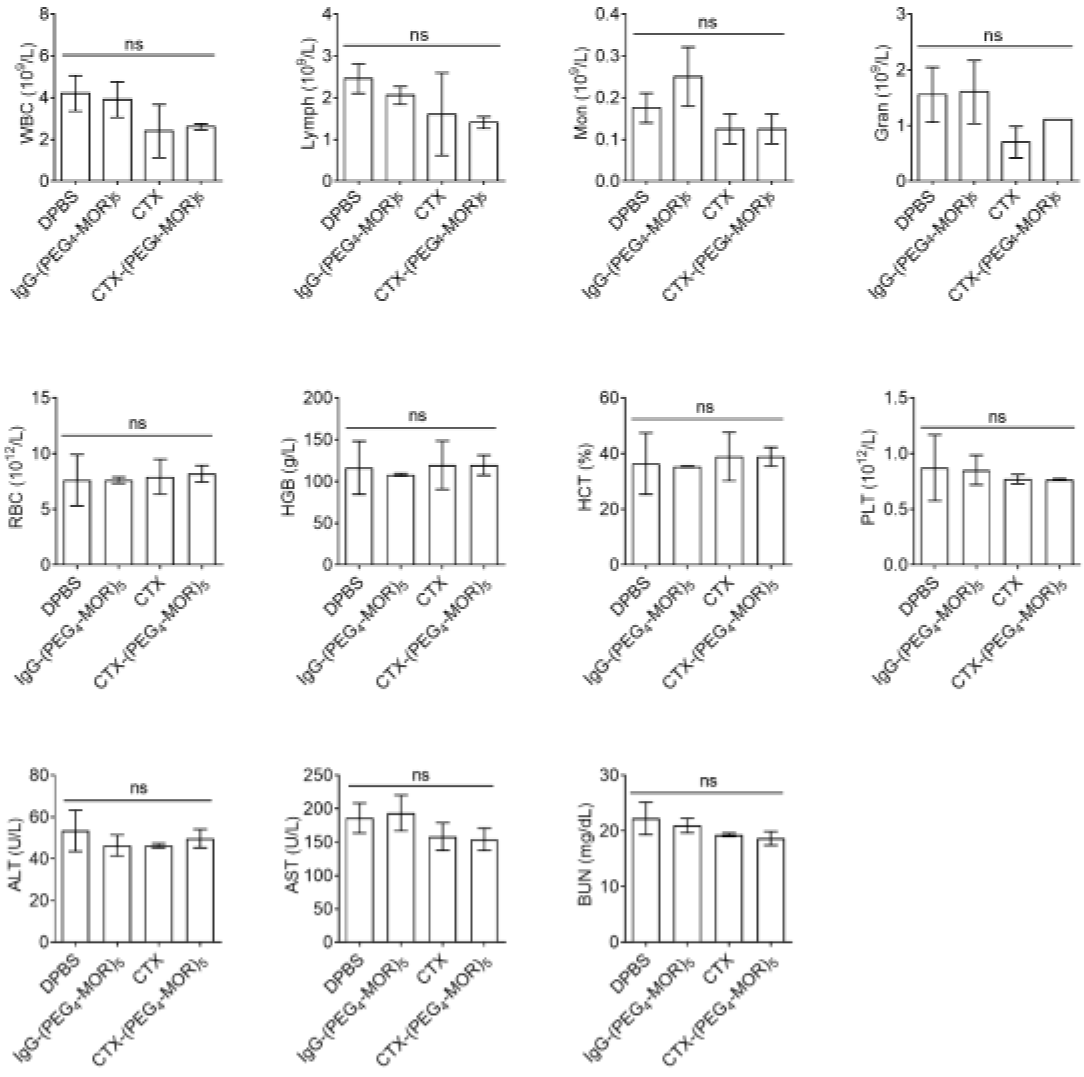

Figure S63. Blood routine test and blood biochemical analysis after A549 tumor-bearing mice were treated with DPBS, IgG-(PEG$_4$-MOR)$_5$, CTX and CTX-(PEG$_4$-MOR)$_5$. WBC: white blood cell, Lymph: lymphocyte, Gran: neutrophil, Mon: monocyte, RBC: red blood cell, HGB: hemoglobin, HCT: hematocrit, PLT: platelet, ALT: alanine aminotransferase, AST: aspartate aminotransferase, BUN: blood urea nitrogen. All data were presented as mean ± SD, n = 3. Statistical significance was determined by one-way ANOVA with a Tukey post hoc test. ns: no significance.

4. Reference

(1) Xia, Y.; Li, J.; Wang, L.; Luo, X.; Xie, Y.; Liu, Y. Spatially Confined Intervention of Cellular Senescence by a Lysosomal Metabolism Targeting Molecular Prodrug for Broad-Spectrum Senotherapy. *Angew. Chem. Int. Ed.* **2022**, *61* (12), e202115764. https://doi.org/10.1002/anie.202115764.

(2) Li, Z.; Theile, C. S.; Chen, G.; Bilate, A. M.; Duarte, J. N.; Avalos, A. M.; Fang, T.; Barberena, R.; Sato, S.; Ploegh, H. L. Fluorophore-Conjugated Holliday Junctions for Generating Super-Bright Antibodies and Antibody Fragments. *Angew. Chem. Int. Ed.* **2015**, *54* (40), 11706–11710. https://doi.org/10.1002/anie.201505277.

(3) Eriksson, I.; Vainikka, L.; Persson, H. L.; Öllinger, K. Real-Time Monitoring of Lysosomal Membrane Permeabilization Using Acridine Orange. *MPs* **2023**, *6* (4), 72. https://doi.org/10.3390/mps6040072.

(4) Chen, K.; Cai, J.; Wang, S.; Li, Y.; Yang, C.; Fu, T.; Zhao, Z.; Zhang, X.; Tan, W. Aptamer Inhibits Tumor Growth by Leveraging Cellular Proteasomal Degradation System to Degrade c-Met in Mice. *Angew. Chem. Int. Ed.* **2023**, 62 (2), e202208451. https://doi.org/10.1002/anie.202208451.

(5) Li, Y.; Liu, X.; Yu, L.; Huang, X.; Wang, X.; Han, D.; Yang, Y.; Liu, Z. Covalent LYTAC Enabled by DNA Aptamers for Immune Checkpoint Degradation Therapy. *J. Am. Chem. Soc.* **2023**, 145 (45), 24506–24521. https://doi.org/10.1021/jacs.3c03899.

(6) Xiao, P.; Wang, J.; Zhao, Z.; Liu, X.; Sun, X.; Wang, D.; Li, Y. Engineering Nanoscale Artificial Antigen-Presenting Cells by Metabolic Dendritic Cell Labeling to Potentiate Cancer Immunotherapy. *Nano Lett.* **2021**, *21* (5), 2094–2103. https://doi.org/10.1021/acs.nanolett.0c04783.
